# Economic burden of chronic obstructive pulmonary disease and post-tuberculosis sequelae in low- and middle-income countries: a database compiled from a systematic review and meta-analysis

**DOI:** 10.1136/bmjph-2023-000441

**Published:** 2024-07-30

**Authors:** Yuling Lin, Alexandra Walker, Marguerite Batta, Sierra Ottilie-Kovelman, Anna Duchenko, Curdin Brugger, Olivia Keiser, Robert S Wallis, Klaus Reither, Fabrizio Tediosi, Marina Antillon

**Affiliations:** 1 Institute of Global Health, University of Geneva, Geneva, Switzerland; 2 Swiss Tropical and Public Health Institute, Allschwil, Switzerland; 3 University of Basel, Basel, Switzerland; 4 Health Policy and Management, Yale University School of Public Health, New Haven, Connecticut, USA; 5 The Aurum Institute, Parktown, South Africa

**Keywords:** economics, Public Health, Endemic Diseases, Comorbidity

## Abstract

**Background:**

Chronic obstructive pulmonary disease (COPD) and tuberculosis (TB) impose a substantial economic burden globally. This systematic review summarised the evidence on the costs of COPD, including post-TB diseases in low- and middle-income countries.

**Methods:**

A systematic review was conducted and studies published between 1 January 2013 and 28 March 2022 (the date of the search) were identified using various electronic databases without language restrictions. Titles, abstracts and full texts were screened in duplicate and data were extracted and verified by reviewers. Eligible studies were categorised as cost analysis and/or economic burden studies, and costs were converted to 2021 United State dollar. Meta-analysis was conducted on the costs of hospitalisations, medication and outpatient visits.

**Results:**

128 cost studies and 65 economic burden studies were included in this review. The data collected are presented in the Cost Database of COPD and Post-TB (CD-CPTB). The majority of studies were from Asia, Eastern Europe and Latin America, with a few from other middle-income countries. There were extremely limited data on the costs of post-TB sequelae and only a few studies were from Africa (n=6) and low-income (n=5) countries. The direct medical costs of COPD ranged from $26 per hospitalisation in India to $2694 per year per patient with severe disease in Mexico, while the costs of acute exacerbation of COPD ranged from $137 to $4207 per exacerbation with both the minimum and maximum costs occurring in Turkey. The costs were lower in lower middle-income countries compared with upper middle-income countries. Finally, considerable economic burden was attributable to smoking and air pollution.

**Conclusion:**

The review and the CD-CPTB database give a thorough snapshot of the current evidence of the costs and economic burden of COPD and post-TB diseases. Future research is needed to investigate the economic impact after TB treatment and should be prioritised in Africa and low-income countries where there has been a lack of data collection.

**Protocol registration number:**

CRD42022326609.

**WHAT IS ALREADY KNOWN ON THIS TOPIC**

- Chronic obstructive pulmonary disease (COPD) imposes a substantial burden of disease in low- and middle-income countries. Understanding the costs across settings and the drivers of these costs may help in designing policy to reduce the financial burden of COPD.
- We searched databases including PubMed, Excerpta Medica Database (EMBASE), EconLit, Cochrane Central Register of Controlled Trials (CENTRAL), NHS Economic Evaluation Database (NHS EED), Web of Science and Global Index Medicus for studies of any design published between 1 January 2013 and 28 March 2022.
- We included studies that estimated the costs of disease to patients or that simulated the costs of disease in subnational, national and regional basis. Our terms were extensive, grouped into two domains, that is, COPD/chronic bronchitis/pulmonary emphysema/post-tuberculosis (TB) and cost/economic burden. Studies had to show either original data or original methods to estimate costs.

**WHAT THIS STUDY ADDS**

- The number of published studies on the costs of COPD, chronic bronchitis and emphysema in low- and middle-income countries has grown substantially since the most comprehensive review published in 2015, which surveyed the literature only until 2013. However, the costs of chronic diseases due to post-TB sequelae have been studied in two publications only.
- To our knowledge, this is the broadest systematic review and meta-analysis of COPD, chronic bronchitis or emphysema in low- and middle-income countries and the first to scope the contribution of TB to COPD-like illness to costs.
- The costs of COPD vary substantially between studies, countries and regions of the world.

**HOW THIS STUDY MIGHT AFFECT RESEARCH, PRACTICE OR POLICY**

- We believe that our findings could facilitate policymaking and further economic evaluations into certain components of healthcare around COPD.
- The costs of hospitalisations, medications and outpatient visits are best documented, while the aggregated costs, such as direct medical, direct non-medical and indirect costs are poorly understood.
- There is a dearth of data from low-income countries as well as countries in Sub-Saharan Africa. Additionally, this study can serve to set research priorities into the costs of COPD.

## Introduction

 Chronic obstructive pulmonary disease (COPD) is characterised by persistent respiratory symptoms and airflow obstruction due to airway and/or lung abnormalities.^1^ Two commonly related conditions involved with COPD are chronic bronchitis (CB), an inflammation of the airways, and emphysema, the destruction of the formations that exchange gases in the lungs.^2^ Tobacco smoking is a well-established risk factor of COPD,^3 4^ while exposure to indoor and outdoor air pollutants, such as biomass smoke and particulate matter, and occupational exposure to dust are also associated with the disease.^1 5^

Some studies have shown evidence that pulmonary tuberculosis (TB) would be an important contributor to COPD.^6^,^9^ Half of those who have completed TB treatment have pulmonary dysfunction, including small abnormalities, airflow obstruction or lung function loss.^10^ A systematic review by Byrne *et al* found that previous TB history is strongly associated with the presence of COPD or bronchiectasis, with a pooled OR of 3.05 (95% CI 2.42 to 3.85; p<0.0001) among people with a history of TB compared with those without TB episode.^11^

Ignoring post-TB sequelae would greatly underestimate the disease burden of TB.^12^ The pooled standardised all-cause mortality rate among people with TB was 2.91 times higher compared with the general population.^13^ After considering the morbidity and mortality of COPD attributable to TB, the estimated burden of TB in India increases by 54%, resulting in an increase of 6.1 million disability-adjusted life years (DALYs) in 2018.^14^ Similarly, the economic impact of TB could also be underestimated by not considering post-TB sequelae, such as COPD.^15 16^ The economic loss due to TB deaths from 2020 to 2050 was estimated at $17.5 trillion.^17^

COPD is the third leading cause of death globally.^18^ COPD was responsible for 3.28 million deaths and 74.43 million DALYs worldwide in 2019, of which approximately 84% of deaths and DALYs occurred in low- and middle-income countries (LMICs).^19 20^ There was a growing trend of COPD prevalence in LMICs from 1990 to 2019. It was predicted that COPD cases will continue to increase in LMICs and will be more than double those in high-income countries (HICs) by 2050.^21^

The economic burden of COPD is considerable across the world. A population-based survey across 12 countries showed that, on a per-patient basis, the annual societal costs, including direct costs—hospitalisations, outpatient visits, emergency room visits, medications and home oxygen—and indirect costs due to income and productivity loss ranged from $1721 in Russia to $30 826 in the USA.^22^ In a lower middle-income country (lower MIC) such as Vietnam, the costs of COPD treatment per year were estimated at $3.3 million, or 0.2% of the gross domestic product (GDP) and 3.8% of the total healthcare expenditure.^23^ Therefore, it is of significance to take COPD into consideration to better understand the lifetime economic burden of TB.

There have been systematic reviews analysing the costs of COPD. A comprehensive review by Srivastava and colleagues was published in 2015, which surveyed the literature until 2013,^24^ along with five more recent reviews. However, these recent reviews face some limitations such as generalisability to LMICs, where TB remains a substantial burden. Some reviews only included studies from HICs,^25^ or from specific regions, such as the Asia-Pacific region^26^ or Europe,^27^ while one review conducted in 2019 was limited to HICs and five MICs and included only direct costs of COPD management.^28^ The most recent review used simple search terms, searched in only a few literature databases, and only included studies published between 2015 and 2020.^29^

This systematic review aimed to summarise the evidence on the costs of COPD, including CB and pulmonary emphysema in LMICs. We subgrouped the costs of patients following TB disease to better understand the lifetime costs of TB.

## Methods

This systematic review was conducted in accordance with the Preferred Reporting Items for Systematic Reviews and Meta-Analyses guidelines.^30^ The review protocol was registered in PROSPERO (registration number CRD42022326609).

### Information source and search strategy

We searched literature published between 1 January 2013 and 28 March 2022 (the date of the search) in various databases, including PubMed, EMBASE, EconLit, Cochrane Central Register of Controlled Trials, NHS Economic Evaluation Database, Web of Science and Global Index Medicus. We performed the search by combining different terms of the two components, that is, the disease component (eg, COPD, CB, pulmonary emphysema, and post-TB) and the cost component (eg, cost, out-of-pocket, expenditure, expense, economic, payment and affordability). We used truncation to capture variations in words. Detailed search strings are shown in online supplemental material 1, Section 1.1. The search was not restricted to any study design or language, as long as studies reported costs of COPD or post-TB in LMICs.

### Study selection

We uploaded the retrieved records to Rayyan, a web application designed to facilitate collaboration and double-blind screening on systematic reviews.^31^ We screened titles, abstracts and full texts in duplicate, based on the eligibility criteria (online supplemental table S1.2). Studies in languages other than English were screened by native speakers, or by reviewers who can read the languages comprehensively, or when necessary, after translating with Google Translate.

Authors were contacted when there was indication that costs of COPD or post-TB could be disaggregated. Studies were included if authors replied with informative data, whereas studies were excluded if authors did not reply 1 week after sending a reminder email.

### Data extraction

One reviewer extracted data in an Excel spreadsheet, while a partner reviewer verified the data. We extracted data on the study characteristics (country, region and year of study, study design, treatment setting and study size), participant characteristics (mean or median age and age range, body mass index and percentage of men/women), diseases (diagnosis, disease severity and length of hospital stay) and costs (source of cost data, method of estimation, direct medical and non-medical costs, indirect costs and total costs). Where available, we also extracted the following data: comorbidities, smoking status, history of TB, exposure to air pollutants and specific cost items (medication, diagnostic service, emergency department visit, outpatient consultation, inpatient hospitalisation, etc).

The primary outcomes are the total, direct medical and non-medical and indirect costs. Direct medical costs include costs from diagnostic services, laboratory services, medications, emergency room visits, outpatient visits and hospitalisations and intensive care unit stays, while direct non-medical costs are related to accessing treatment, for example, costs due to travel, accommodation and meals. Direct costs include direct medical and non-medical costs. Lost earnings and productivity are considered indirect costs. Total costs are the sum of direct and indirect costs.

### Quality assessment

Paired reviewers assessed the quality of each study independently using criteria adapted from the Joanna Briggs Institute (JBI) checklist for economic evaluations^32^ and the Consensus on Health Economic Criteria (CHEC),^33^ together with consideration of Global Health Cost Consortium (GHCC) Reference Case for Estimating the Costs of Global Health Services and Interventions^34^ and Consolidated Health Economic Evaluation Reporting Standards (CHEERS).^35^ The adapted quality assessment tool is presented in online supplemental material 1, Section 1.3. Possible answers for each criterion are ‘yes’, ‘no’, ‘unclear’, and ‘not applicable’. The descriptive statistics of the quality assessment results were presented in the results section, but because there is no established threshold of quality, studies were not categorised based on the quality.

### Data synthesis

Included studies are generally categorised as cost analysis studies and economic burden (over a population) studies. The costs were converted to 2021 United State dollars (USD). Economic burden estimates were compared with national health expenditure (HE) of the same year^36^ or GDP per capita and population of that country in the same year, while HE and GDP per capita were converted to 2021 values.^37^

Study characteristics were presented in descriptive tables, and study data were quantitatively synthesised if we had at least three studies measuring costs. For meta-analysis, we used an inverse-variance weighted log-transformed model to estimate the costs of COPD due to the skewness of the data and we used a random effects model to account for unmeasured heterogeneity between studies, which is quantified using the *I^2^* (i-squared) statistic, and tested with a τ^2^ statistic.^38^ To investigate factors that contribute to heterogeneity of cost estimates across studies, we performed subgroup analyses by World Bank income group,^39^ continent and country where applicable. Between-subgroup differences were tested with a χ^2^ statistic on the random-effects model.

Analyses were done with the R package ‘meta’ using a function to calculate overall means from studies reporting means on a single sample.^40^ The package can also estimate approximate means and SDs when medians, IQRs or ranges, and sample sizes are reported.^41^ The cost data are presented in the form of a database and available to the public on GitHub for future use or analysis.

### Patient and public involvement

It was not appropriate or possible to involve patients or the public in the design, or conduct, or reporting, or dissemination plans of our research.

## Results

### Literature search

**Figure 1:**
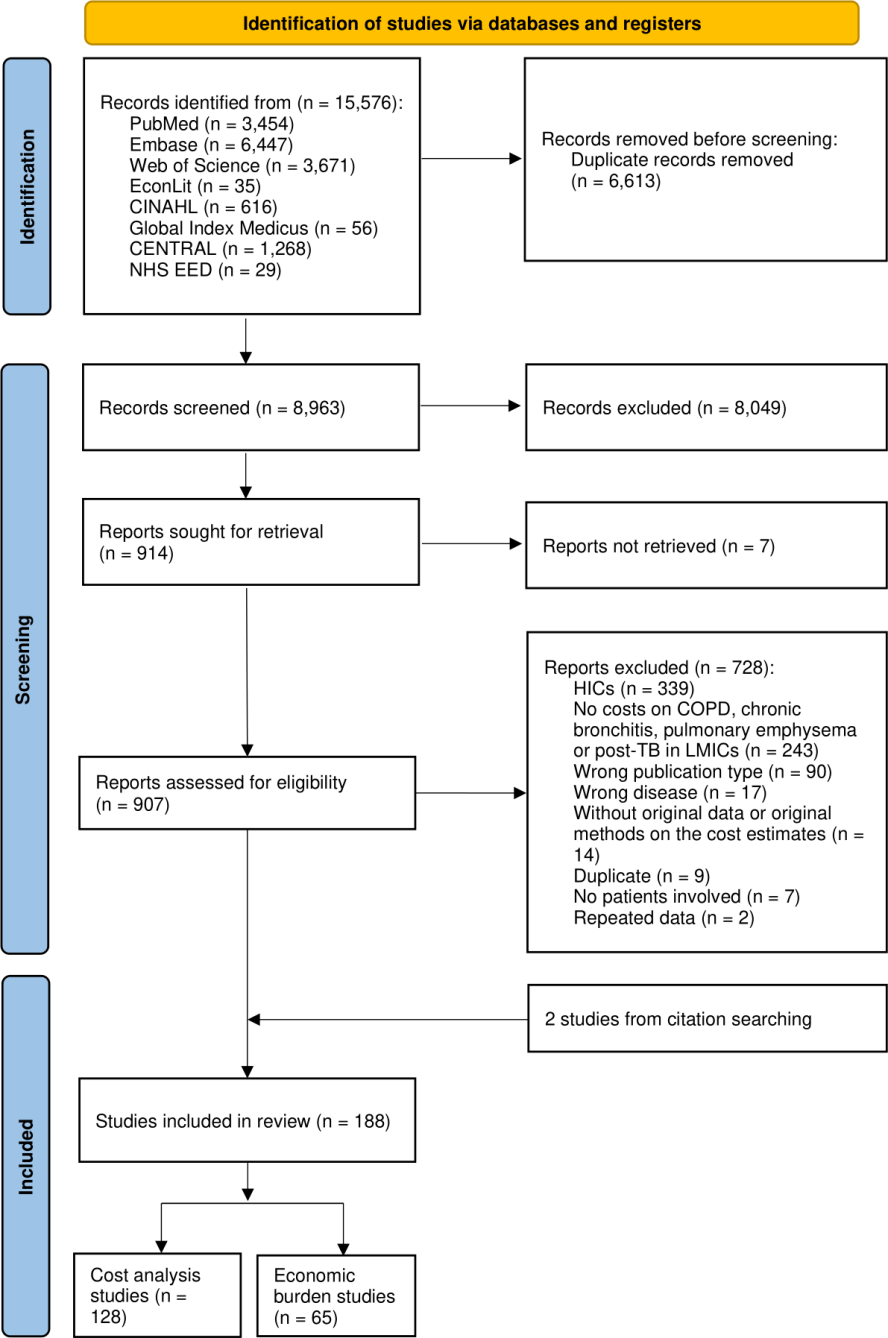
Flowchart of the identification of studies. Adapted from The PRISMA 2020 statement: an updated guideline for reporting systematic reviews.^69^ COPD, chronic obstructive pulmonary disease; HICs, high-income countries; LMICs, low- and middle-income countries; PRISMA, Preferred Reporting Items for Systematic Reviews and Meta-Analyses; TB, tuberculosis.

Identification, exclusion and inclusion of the studies were reported in a flowchart (figure 1). After removing duplicates, 8963 records were identified from databases. Of 8049 records were excluded during title and abstract screening and seven full texts were not retrieved due to a lack of access via University of Geneva, University of Basel and Yale University. Of the 907 studies that went through full-text review, 728 studies were excluded at this stage. Together with two studies included from citation searching, there were 188 eligible studies for review. There were 128 cost analysis studies and 65 economic burden studies, five of which reported both on cost and economic burden. Online supplemental material 1, Section 1.10, lists all the included studies.

### Study characteristics

#### Cost analysis studies

**Figure 2:**
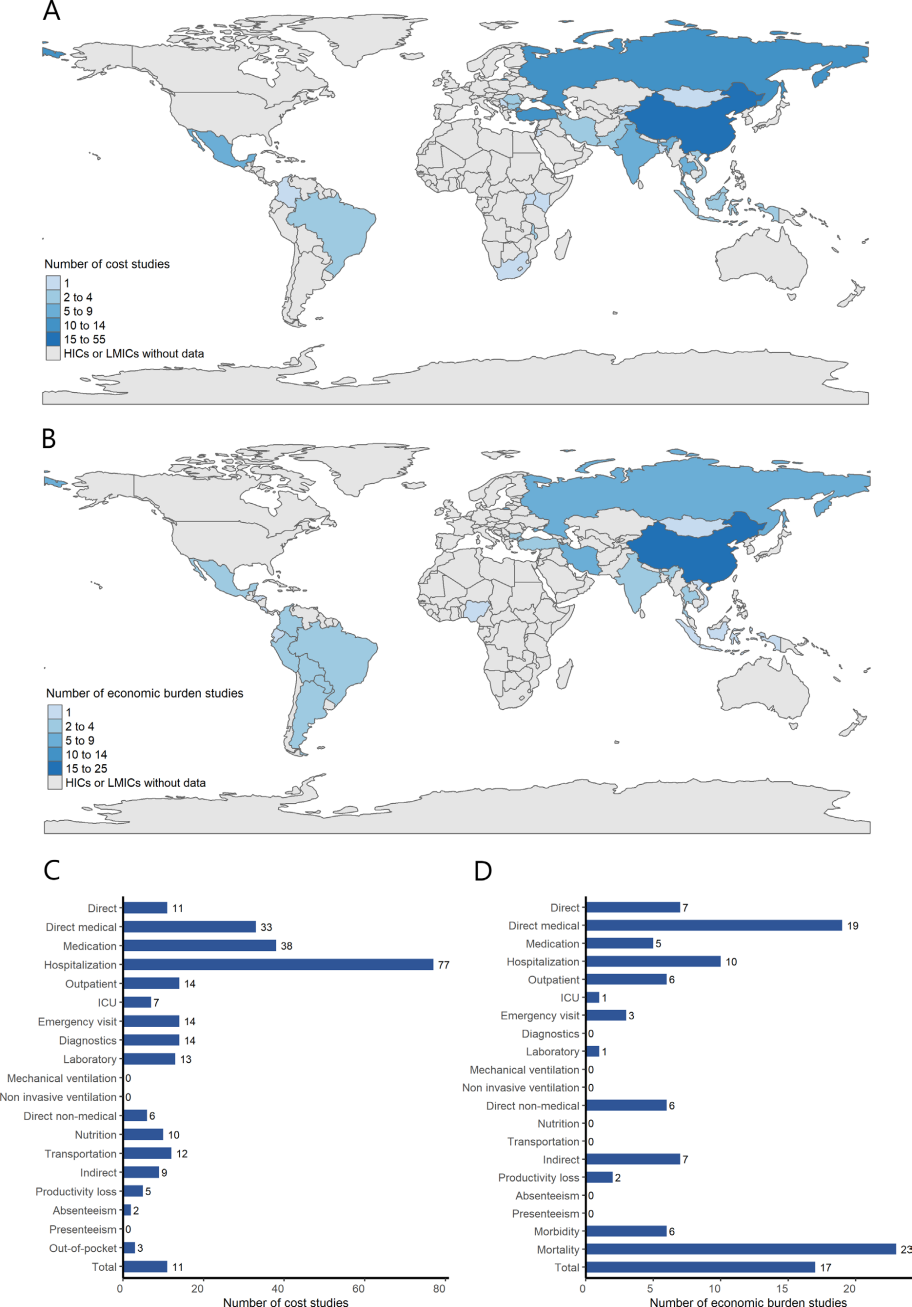
Countries of studies and number of studies reporting data on cost outcomes (**A**) countries of cost studies; (**B**) countries of economic burden studies; (**C**) number of cost studies reporting data on cost outcomes; (**D**) number of economic burden studies reporting data on costs outcomes. Countries of studies that aggregated estimates across countries were not presented on the map. Direct costs include direct medical and non-medical costs. ICU, intensive care unit.

The eligible cost analysis studies span over 9 years between January 2013 and March 2022 (table 1). The single year with the largest number of studies (19.5%, n=25) was 2021. Overall, 78.1% of the studies (n=100) were conducted in upper MICs, 19.5% (n=21) in lower MICs, and only three studies (2.3%) were in low-income countries. As shown in table 1 and figure 2A, most studies (91.4%, n=117) were from Asia and Eastern Europe and only five and seven studies were from Africa and Latin America, respectively. The most common countries among the studies were China (43.0%, n=55), Turkey (9.4%, n=12), Russia (8.6%, n=11), India (7.0%, n=9), Thailand (5.5%, n=7), Vietnam (3.1%, n=4) and Iran (2.3%, n=3).

**Table 1:** Characteristics of cost analysis studies

Literature characteristics	Category	Number of studies*	Percentage
Publication year	2013	7	5.5
	2014	12	9.4
	2015	10	7.8
	2016	16	12.5
	2017	10	7.8
	2018	16	12.5
	2019	14	10.9
	2020	15	11.7
	2021	25	19.5
	2022	3	2.3
Geographical region	Africa	5	3.9
	Asia	96	75.0
	Eastern Europe	21	16.4
	Latin America	7	5.5
World Bank income group	Low income	3	2.3
	Lower middle income	25	19.5
	Upper middle income	100	78.1
Country	China	55	43.0
	Turkey	12	9.4
	Russia	11	8.6
	India	9	7.0
	Thailand	7	5.5
	Vietnam	4	3.1
	Iran	3	2.3
	Other	28	21.9
Disease	COPD	75	58.6
	AECOPD	48	37.5
	COPD/AECOPD	1	0.8
	Asthma-COPD overlap	1	0.8
	COPD/asthma	1	0.8
	Chronic bronchitis	3	2.3
	Post-TB sequelae	1	0.8
With intervention	No	86	67.2
	Yes	42	32.8

* The total number of cost analysis studies is 128, which is the denominator for percentages. One study^22^ collected costs of COPD across 3three countries and 2two geographic regions. Two studies^70 71^ reported costs for 2two diseases. Therefore, the sum of the number of the studies across regions, countries and diseases is more than 128.

AECOPDacute exacerbation of COPDCOPDchronic obstructive pulmonary diseaseTBtuberculosis

As figure 2C demonstrates, total direct costs were collected in 11 studies, total direct medical costs in 33 studies and total indirect costs in 9 publications. Otherwise, studies presented components of direct or indirect costs but not always the total: 77 studies reported hospitalisation costs, 38 studies reported medication costs and 14 studies reported the costs of emergency room visits.

#### Economic burden studies

The included economic burden studies cover more than 9 years from January 2013 to March 2022, and most were published in the years from 2016 to 2021 (online supplemental table S1.4). The majority of the studies were conducted in Asia (61.5%, n=40), Eastern Europe (23.1%, n=15) and Latin America (10.8%, n=7), whereas only one study was conducted in Africa (as shown in online supplemental table S1.4 and figure 2B). Similarly, only two publications reported data targeting low-income countries, while the rest were in lower or upper MICs. More than one quarter of the studies (36.9%, n=24) were conducted in China, 9 (13.8%) in Russia and 6 (9.2%) in Iran.

Figure 2D illustrates that seven economic burden studies estimated the direct cost burden, 19 studies estimated direct medical cost burden, 7 studies reported indirect burden and 17 studies reported total societal burden. There were 23 publications estimating mortality-related economic burden and six estimating morbidity-related burden.

### Study quality

Quality assessment results (online supplemental table S1.5) indicate that the study population was generally well described in most of the cost analysis (76.6%, n=98) and economic burden (66.2%, n=43) studies, whereas only 28.1% (n=36) of costing studies and 40.0% (n=26) of economic burden studies indicated the perspective of costs. Among 128 costing studies, 60.2% (n=77) and 51.6% (n=66) clearly described the disease diagnosis and measures of costs, and 68.0% (n=87) clearly indicated the year of costs collected, while 30.8% (n=20), 75.4% (n=49) and 76.9% (n=50) economic burden studies clearly defined the disease, measures of costs and the year of costs, respectively. There were 41 publications of economic burden that estimated the burden in the future, of which, 19 were observed to be adjusted for differential timing, or the adjustment of economic costs in the future to present-day values was conducted through discounting.

The quality assessment results of each study can be accessed in an online GitHub repository.

### Database

The data are presented as a Cost Database of COPD and Post-TB (CD-CPTB) for any further analysis of interested researchers. The database is stored in the open access online GitHub repository.

### Economic burden of post-TB

There was only one costing study investigating post-TB healthcare costs.^42^ One year after TB treatment, 66.8% patients in urban Blantyre, Malawi had ≥1 outpatient visits with median costs of $1.34, and 6.3% patients had ≥1 inpatient admissions (27 in total) with median costs of $25.0 and median length of stay (LOS) of 4 days. In addition, one-fifth of participants with paid or unpaid work before diagnosis had no work 1 year after TB treatment, with 47.3% of them losing work during TB and its treatment and 52.7% losing work during the year after treatment completion. The study also indicated the impact of post-TB on households. The authors observed that 9.5% of households affected with TB had interruption of the children’s schooling, and 16.9% of patients reported that they suffered severe financial hardship from TB 1 year after treatment completion.

There was one study estimating the economic burden among patients with TB diagnosis.^43^ Patanavanich and colleagues estimated that in Thailand from 2007 to 2014, the economic burden due to hospital admissions of COPD patients with TB diagnosis was $31.8 million, representing 2.3% of the total costs among all COPD patients. The same study also presented that the burden due to hospital admissions among bronchitis and emphysema patients with TB diagnosis was $13.6 million, representing 41% of the total costs among all bronchitis and emphysema patients. Per admission, the costs for COPD patients in general were $1319.00, whereas the costs for COPD patients with TB diagnosis were $875.50. Among patients with bronchitis and emphysema, the costs in general were $2183.20 per admission and the costs for those patients who were also diagnosed with TB were $1455.40 (correspondence with authors).

### Costs of COPD

Online supplemental tables S2.1 and S2.2 display costs of COPD among non-intervention studies by World Bank country income group and continent. The hospitalisation costs per year per patient of COPD ranged from $59 in Vietnam to $3240 with moderate COPD and $5775 with severe COPD in Mexico. Medication costs varied both in magnitude and unit of reporting: from $2 per hospitalised patient with Global Initiative for Chronic Obstructive Lung Disease (GOLD) II disease level in India, $9 per prescription in South Africa, to $1281 per year per patient in Thailand.

Among lower MICs, the minimum direct medical costs were $26 per hospitalisation among patients with GOLD II disease level in India and the maximum costs were $1720 per patient in private facilities in Kenya. In upper MICs, direct medical costs ranged from $428 per year per patient in Thailand to $2694 per year per patient with severe COPD in Mexico. Direct non-medical costs ranged from $3 per hospitalisation to $132 per hospitalisation in India. The highest indirect cost reported was $147 per patient in Vietnam.

### Costs of AECOPD

As shown in online supplemental table S2.3, among lower MICs, hospitalisation costs of acute exacerbation of COPD (AECOPD) ranged from $299 per GOLD I patient in Vietnam to $985 per year per patient and $1942 per patient with LOS over 9 days in Iran, and medication costs ranged from $49 per exacerbation in Pakistan to $759 per patient with LOS over 9 days in Iran. The minimum direct medical costs were $631 per patient with LOS of less than 9 days in Iran and the maximum direct medical costs were $3232 per patient with LOS over 9 days in Iran.

Among upper MICs, hospitalisation costs ranged from $46 per exacerbation among patients with no comorbidities in Turkey to $5732 per year per patient in China, while it could reach $15 442 per patient in a group of patients for whom non-invasive positive pressure ventilation failed in China. Medication costs ranged from $70 per exacerbation among patients with no comorbidities in Turkey to $1898 per hospitalisation in China, and direct medical costs differed from $137 per exacerbation without comorbidities in Turkey to $4207 per exacerbation requiring intubation in Turkey.

### Costs of CB

There were three intervention studies reporting costs of CB (online supplemental table S2.5). Xuan and colleagues estimated the direct medical costs at $146 and $121 per acute exacerbation in China using Broncho-Vaxom in managing respiratory tract infections and using standard care therapy, respectively.^44^ Zhu *et al* reported average direct medical costs of $1829 and $2233 in China among patients receiving a combined treatment of cephalosporin with herba houttuyniae versus patients receiving cephalosporin only.^45^ A study by Ignatova *et al* reported average indirect costs of $15 and $46 per year per patient in Russia for those with and without vaccination with pneumococcal 13-valent conjugate vaccine, respectively.^46^

### Data from additional cost studies

The costs of studies that did not present data stratified by disease (COPD, AECOPD and CB) but instead presented data for combinations of groups of patients are described in online supplemental material 1, Section 1.6.1. The costs from intervention studies are described in described in online supplemental material 1, Section 1.6.2.

### Economic burden of COPD

Online supplemental table S3.1 shows the economic burden of COPD by country. The direct medical cost burden of COPD was estimated as $97.7 million from the perspective of the healthcare system in Russia (0.12% of HE) $108.1 million in Paraguay (4.8% of HE), $285.4 million in Turkey (1.1% of HE) and $3040.9 million in China (0.43% of HE), while the costs attributable to smoking ranged from $15.5 million in Honduras (0.86% of HE) to 4004.2 million in Brazil (2.9% of HE)). Moreover, the direct cost burden was $103.4 million in Russia (0.13% of HE) and $294.3 million in Peru (3.1% of HE) and the burden attributable to smoking was $10.5 million in Russia (0.13% of HE), $226.1 million in Peru (2.4% of HE) and $401.9 million in Vietnam (4.3% of HE).

There were some studies estimating the economic burden of premature mortality due to COPD. It was $40.8 million in Iran (where the GDP per capita was $12 816 and the population was 80 million) and $2705.2 million in Russia (where the GDP per capita was $10 139 and the population was 144.3 million); the burden attributable to PM₂.₅ pollution was $717.3 million in Nigeria (where the GDP per capita was $3204 and the population was 184 million) and $6903.9 million in Thailand (where the GDP per capita was $6487 and the population was 70.6 million); the burden attributable to smoking ranged from $102.4 million in Vietnam (where the GDP per capita was $2379 and the population was 88.3 million) to $515.7 million in Russia (where the GDP per capita was $10 139 and the population was 144 million).

Economic burden studies were further summarised and described in online supplemental material 1, Section 1.7.

### Meta-analysis

We performed meta-analyses on the costs of hospitalisations, medications and outpatient visits due to the availability of data on these cost components. No meta-analysis was conducted on the total direct medical and non-medical and indirect costs because the measure of the overall costs varied across studies and data were usually not available.

#### Hospitalisation costs

**Figure 3:**
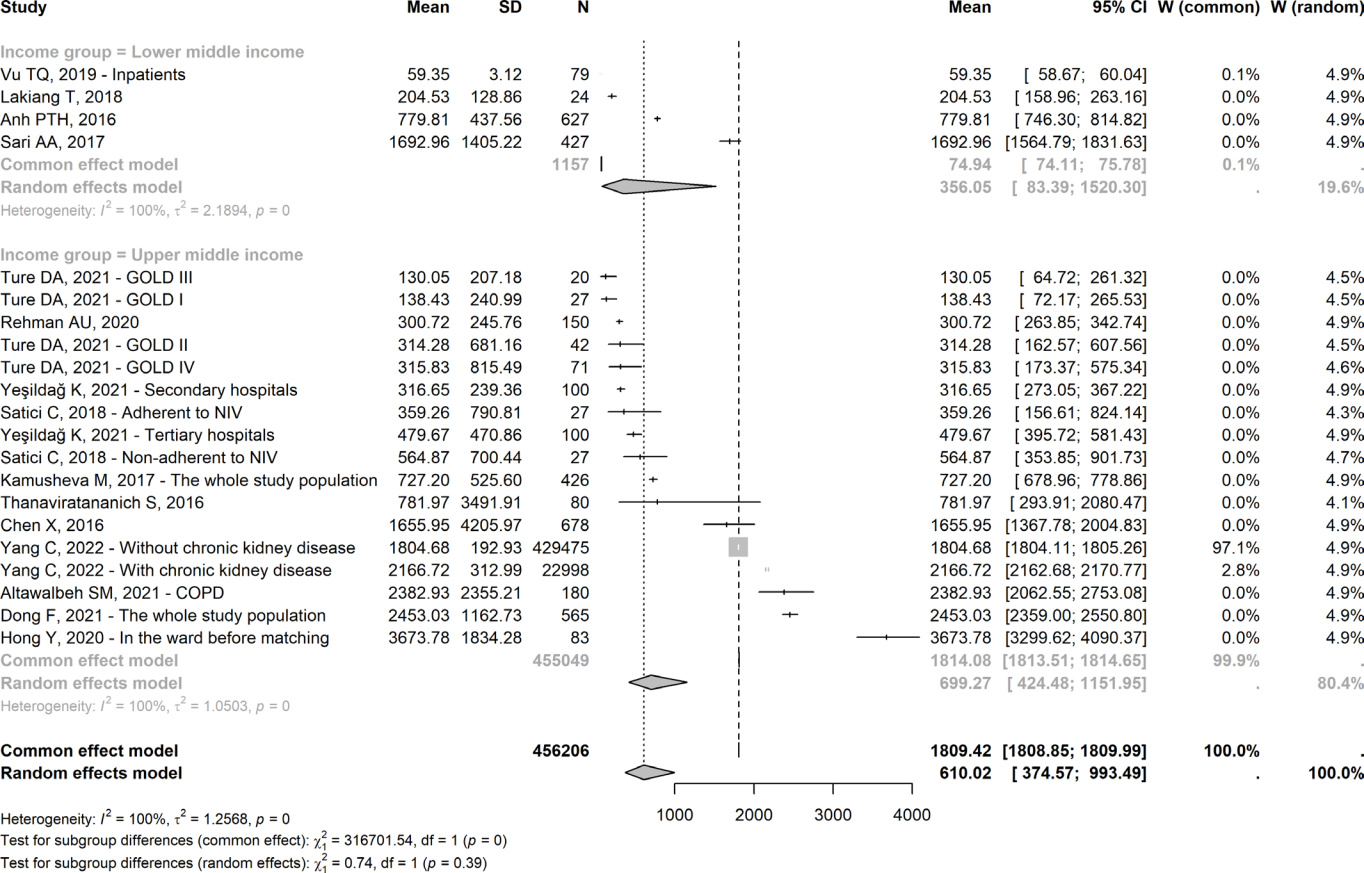
Meta-analysis of hospitalisation costs in 2021 USD of COPD by income group (W denotes weight). COPD, chronic obstructive pulmonary disease.

Figure 3 shows the overall pooled random effects estimates of hospitalisation costs of COPD across non-intervention studies and subgroup estimates according to the World Bank income group of the study’s country for the fiscal year 2021–2022. The pooled hospitalisation costs of COPD were $610.02 and the overall estimates were $356.05 and $699.27 in lower and upper MICs, respectively. The heterogeneity across studies within each income group was statistically significant (p<0.01) but the between-subgroup differences were not significant (p=0.39).

**Figure 4:**
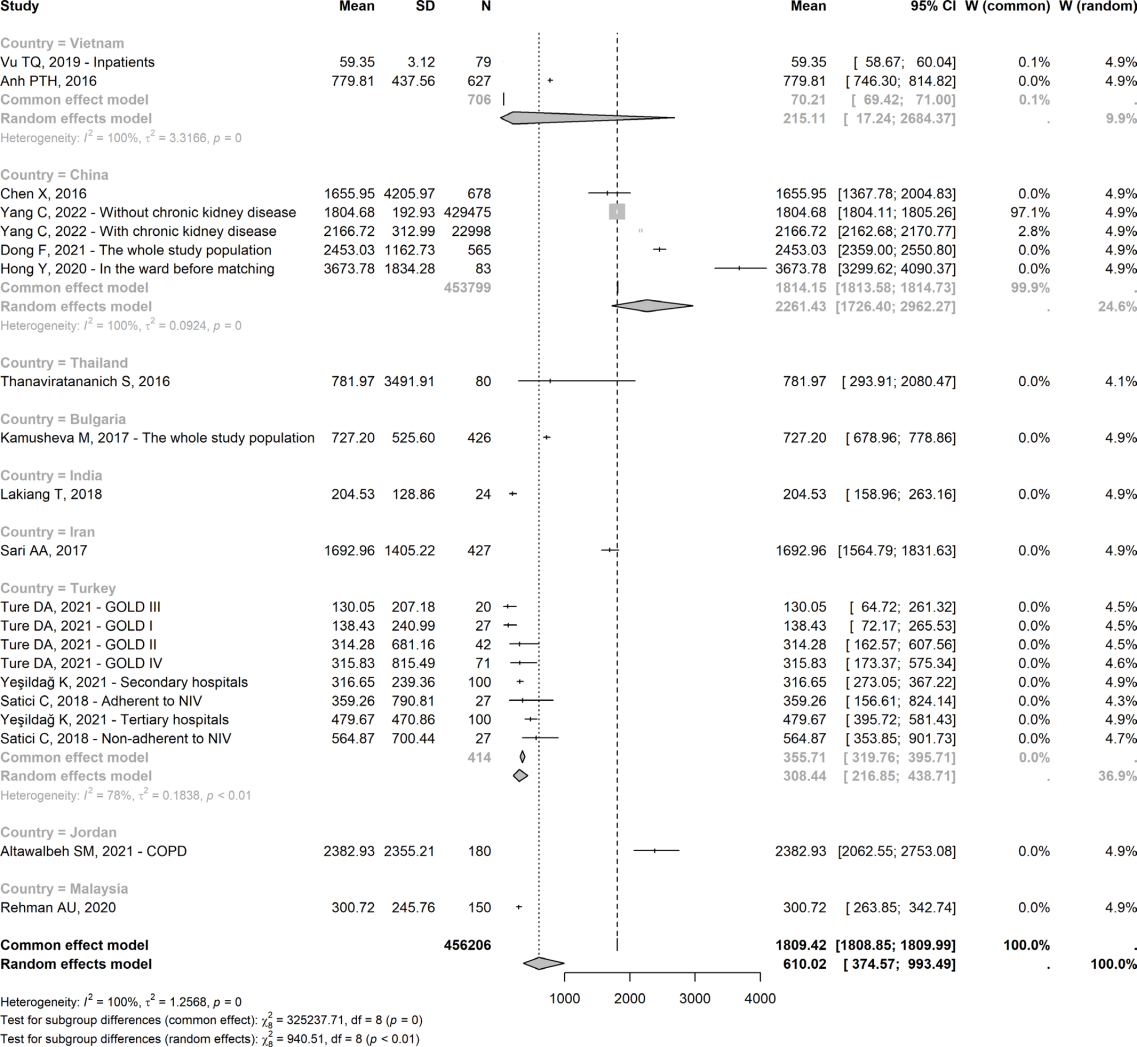
Meta-analysis of hospitalisation costs in 2021 USD of COPD by country (W denotes weight). COPD, chronic obstructive pulmonary disease.

The estimated hospitalisation costs across Asian countries were $621.24 and $627.79 in Eastern Europe, and between-continent random effects were not significantly different (p=0.97) (online supplemental figure S4.1). Figure 4 indicates a high degree of heterogeneity across countries with the first two highest estimates in Jordan and China ($2382.93 and $2261.43, respectively) and significant between-country differences (p<0.01). The results did not differ significantly between the group of studies that measured costs per patient and per hospitalisation event (p=0.78) with a pooled cost of $610 with slightly higher costs per event than per patient—although without significance (online supplemental figure S4.2).

The overall pooled random effects estimate of hospitalisation costs of AECOPD was $1812.40 and the heterogeneity across studies was statistically significant (p≤0.001) (online supplemental figure S4.3 and S4.4). The subgroup estimates were $755.06, $1919.66 among lower and upper MICs, respectively, and $394.62 and $2008.67 among Eastern European and Asian countries, respectively, with statistically significant differences between income groups (p=0.02) and continents (p≤0.01). An analysis by country showed statistically significant heterogeneity between studies within single countries and across countries (p≤0.01) (online supplemental figure S4.5). When we analysed the costs of AECOPD by calculation unit (either per event or per person), there was significant heterogeneity between subgroups (p<0.01) with a higher cost per event at $2507.44 versus per patient $1468.37 (online supplemental figure S4.5).

#### Medication costs

The pooled estimate of medication costs of COPD was $155.76 with a higher estimate in upper MICs than in lower MICs ($280.26 vs $48.56, p<0.01) and the between-country differences were significant (p<0.01) (online supplemental figures 4.7 and 4.8). A similar trend was observed for the costs of AECOPD with an overall random effects estimate of $360.57, but no significant difference between income groups was detected ($379.33 and $298.13 for upper and lower MICs, respectively, p=0.62) (online supplemental figure S4.9). However, the between-country differences were significant (p<0.01) (online supplemental figure S4.10).

#### Outpatient costs

The overall random effects estimate of outpatient costs of COPD was $158.77; the pooled estimate was $26.17 and $525.87 in lower and upper MICs, respectively; and the between-country differences were also significant (p<0.01) (online supplemental figure S4.11 and S4.12). There were no data available for meta-analysis on the outpatient costs of AECOPD.

## Discussion

This systematic review found that COPD poses large economic impact on countries across continents. We found that there were limited data on the costs of post-TB sequelae from the literature. There were only two studies on the cost of COPD-like symptoms on patients with a previous diagnosis of TB. These studies indicated a high economic impact after TB treatment. In urban Blantyre, Malawi, around two-thirds of patients sought healthcare during the year after TB treatment and one-fifth lost the work they had prior to TB, while some households faced financial difficulties and interruption of children’s schooling.^42^ In Thailand, patients with diagnosed TB accounted for 2.3% of the total economic burden of hospital admissions of COPD and 41% of bronchitis and emphysema.^43^

Yet, these studies did not clinically define post-TB disease but stated that patients had completed TB treatment for 1 year or that patients had a TB diagnosis. Some studies showed evidence that TB is associated with the presence of COPD,^6^,^911^ whereas some scholars argued that the pathophysiologic process in COPD and post-TB obstructive airway diseases are different.^10 47^ The features of COPD due to smoking can be different from COPD due to post-TB disease, and whether chronic airflow limitation due to other diseases such as TB should be defined as COPD needs further research.^48^ Although the cause of COPD, whether it is smoking or previous TB, might make a difference in terms of the symptoms experienced, it may not make a substantial difference in terms of resources spent. Therefore, as there is no better proxy at the moment, the cost of COPD (even if caused by smoking) will have to be the most appropriate estimate of COPD caused by TB. Overall, TB adds substantial risk and burden to chronic lung and respiratory diseases.^1149^,^51^ The lifetime burden of post-TB disease was estimated to be 58 million DALYs (95% uncertainty interval, 38–83), globally accounting for 47% of the overall burden caused by TB.^12^ More work should be conducted to investigate the economic impact of post-TB or the lifetime costs of TB.

There were also a limited number of cost analysis studies conducted in low-income countries and in Africa (three and five studies, respectively), which bear a large proportion of the disease burden of TB^12^ and chronic respiratory disease.^19^ This suggests that more efforts and priorities should be put to collect or generate quality data in resource-limited countries to inform local governments and international organisations to take action to reduce the global disease burden of COPD.

Similar to a previous systematic review that comprehensively searched the literature published between 2003 and 2013,^24^ variation in country of study, data source, perspective of cost, disease diagnosis, disease severity, comorbidity and patient characteristics generally exists and makes it challenging to compare costs across different studies and countries. However, both reviews indicate COPD contributed a considerable burden to society.

The costs of COPD in LMICs seemed to be lower than in HICs when comparing to the evidence from HICs collected by other reviews.^25 27 28^ However, the cost range is large in both LMICs and HICs, and it could be due to differences in labour costs, the costs of medicines and other medical supplies and the healthcare services provided. For example, our review found that hospitalisation costs of COPD ranged from $59 per patient in Vietnam^52^ to $3240 per year per patient in Mexico with the highest annual costs—$5775 per patient—occurring among patients with severe disease^53^; the costs of AECOPD ranged from $46 per exacerbation^54^ to $5732 per year per patient^55^ while reaching $15 442 per patient among those with non-invasive positive-pressure ventilation failure.^56^ Anees ur Rehman *et al*^28^ found that the hospitalisation costs of COPD ranged from $925^57^ to $6929^58^ in Europe and $7242 in the USA with costs due to severe exacerbation reaching $20 757.^59^

We also found lower costs in lower MICs in comparison to upper MICs. Direct medical costs of COPD ranged from $26 per hospitalisation in India^60^ to $1720 per patient in private facilities in Kenya,^61^ while among upper MICs, the minimum direct medical cost was $428 per year per patient in Thailand^62^ and the maximum was $2694 per year per patient with severe disease in Mexico.^53^ Similarly, the range of direct medical costs of AECOPD in lower MICs was $631–$3232 per patient with both costs occurring in Iran,^63^ while in upper MICs, it ranged $137–$4207 per exacerbation, both estimates from studies conducted in Turkey.^54^ This finding is also supported by the subgroup analysis, but there were high heterogeneities across populations and countries.

An advantage of the present systematic review compared with previous ones is that we explored the possibility of performing meta-analyses while taking into account heterogeneity across studies and subgroups. Heterogeneity is high within and between subgroups, possibly due to differences in study setting, disease severity and comorbidity between studies, and labour costs between countries. Meta-regression was not performed because cost data were sparsely distributed across disease and participant characteristics, resulting in limited data to explore the sources of heterogeneity using meta-regression. However, we conducted subgroup analyses to examine differences in costs between countries, regions, income groups and cost units (such as costs per event or per patient).

Demographic factors, such as sex and age, and health factors, such as comorbidities and disease severity, might have an impact on the costs (online supplemental tables S2.8–S2.11). Costs were higher among women, the elderly, patients with comorbidities and those with more severe disease. Future research should further investigate the factors that contribute to the cost burden.

There were no data on the cost of invasive or non-invasive mechanical ventilation, but there were studies reporting costs on oxygen^64^ or home oxygen therapy.^22^ Home oxygen costs ranged from $91 in Brazil to $168 in Russia. There were limited data on indirect costs from cost analysis studies.

On the other hand, high economic burden attributable to air pollution and smoking was documented for several countries. Direct medical costs of COPD due to smoking ranged from 15.5 million in Honduras (0.86% of HE) to 4004.2 million in Brazil (2.9% of HE),^65^ and the economic burden of premature mortality attributable to PM₂.₅ pollution was $717.3 million (correspondence with authors) in Nigeria (where the GDP per capita was $3204 and the population was 184 million)^66^ and $6903.9 million in Thailand (where the GDP per capita was $6487 and the population was 70.6 million).^67^ The estimates could differ due to author’s different methods of estimation.

One strength of our study is the breadth of literature that we examined. The review covers publications over 9 years and across LMICs without language restrictions. However, both the quality of the studies and the heterogeneity between the studies warrant caution at the time of interpretation.

The present review also faced some limitations. First, although this review covers the literature from 2013 up to 2022, the data may already be outdated at the time of publication. Future research should update the literature search and data accordingly. Second, our data were not extracted in duplicate due to time constraints, but instead we chose an approach of validation with a peer. Third, while our quality assessment was performed as consistently as possible, there is no conventional standard on which to judge all studies, and, therefore, we chose an assessment of the presence and absence of the characteristics mentioned in the JBI checklist, CHEC, GHCC and CHEERS. Moreover, the data presented challenges at the time of synthesis, as there were inconsistencies in the summary statistics that were presented; nonetheless, the package we used was able to approximate means and SDs when there was a measure of central tendency (mean or median), spread (IQR or range) and the sample size.

Meta-analyses were focused on hospitalisations, medications and outpatient visits, but the data might support a wealth of other subgroup analyses of interest. Heterogeneity is potentially biased upward^68^ due to the small number of studies in the subgroup analysis and variation in disease and participant characteristics across studies.

Meta-regression was not performed due to the sparse distribution of cost data across disease and patient characteristics and the limited data available on factors that may influence costs. Costs can vary widely between health systems and countries. Costs are influenced by several factors including the economic development of the country, the functioning of the health system, the income level of patients, the availability and coverage of health insurance, the severity of the disease, the presence of comorbidities, the perspective of the study and, therefore, the types of costs included, and other relevant factors. Apart from the level of economic development of the countries, data on most of the other factors are very limited, as presented in the CD-CPTB database. This makes it challenging to conduct and interpret meta-regression to further explore sources of heterogeneity.

Finally, it was often hard to categorise the costs per patient or per event (hospitalisation, outpatient visit or exacerbation), but with chronic conditions, a more appropriate appraisal of the costs would be all the hospitalisations and doctor’s visits over a certain period of time.

## Conclusions

Our database gives a thorough overview of the knowledge of COPD costs between 2013 and 2022, updated to 2021 values. We determined that there is a lack of data on the cost and economic impact of TB infection on chronic respiratory diseases and of the estimate from future chronic conditions on the estimates of the cost of a TB case in the present. While there is substantial heterogeneity on COPD costs between countries, there are numerous studies from the Americas and the Asian regions, but a near-complete lack of information from Africa. Meta-analyses and subgroup analyses indicate costs of COPD and AECOPD vary across countries, regions, income groups and cost units. Our review may inform policymakers throughout LMICs, but it may also help researchers strategise on the selection of settings for future studies.

## supplementary material

10.1136/bmjph-2023-000441 online supplemental file 1

10.1136/bmjph-2023-000441 online supplemental file 2

10.1136/bmjph-2023-000441 online supplemental file 3

10.1136/bmjph-2023-000441 online supplemental file 4

## Data Availability

All data relevant to the study are included in the article or uploaded as supplementary information.
