# Supplementary material for: Economic burden of chronic obstructive pulmonary disease and post-tuberculosis sequelae in low- and middle-income countries: a database compiled from a systematic review and meta-analysis
Source: BMJ Public Health. 2024 Jul 30;2(1):e000441. doi: 10.1136/bmjph-2023-000441 (PMC11816951; doi:10.1136/bmjph-2023-000441)
Supplement: online supplemental file 1 [file bmjph-2-1-s001.pdf]

The economic burden of chronic obstructive pulmonary disease and post-tuberculosis sequelae in low- and middle-income countries: a database compiled from a systematic review and meta-analysis

Yuling Lin[1], Alexandra Walker[2,3], Marguerite Batta[2,3], Sierra Ottilie-Kovelman[2,4], Anna Duchenko[2,3], Curdin Brugger[2,3], Olivia Keiser[1], Robert S. Wallis[5], Klaus Reither[2,3], Fabrizio Tediosi[2,3] , Marina Antillon[2,3*]

1 Institute of Global Health, University of Geneva, 1205 Geneva, Switzerland
2 Swiss Tropical and Public Health Institute (Swiss TPH), Kreuzstrasse 2, 4123 Allschwil, Switzerland
3 University of Basel, Peterspl. 1, 4001 Basel, Switzerland
4 Yale School of Public Health, New Haven, Connecticut 06510, United States
5 The Aurum Institute, Johannesburg, South Africa
*Corresponding author

Supplemental Methods and Results

Contents

1.1 Search strategy 1
1.1.1 PubMed databases (2013/01/01-2022/03/28) 1
1.1.2 Embase (2013-2022) 1
1.1.3 Web of Science (2013.01.01 – 2022.03.28) 2
1.1.4 EconLit via EBSCOhost (2013.01.01 – 2022.03.31) 4
1.1.5 CINAHL via EBSCOhost (2013.01.01 – 2022.03.31) – CINAHL subject headings 5
1.1.6 Global Index Medicus databases (2013-2022) – indexed with MeSH terms 6
1.1.7 CENTRAL (The Cochrane Central Register of Controlled Trials) - via Cochrane library (2013-2022) – indexed with MeSH terms 6
1.1.8 Cochrane National Health Service Economic Evaluation Database (NHS EED) - via https://www.crd.york.ac.uk/CRDWeb/ (2013-2022) - indexed with MeSH terms 7
1.2 Eligibility criteria 8
1.3 Quality assessment tool 8
1.4 Characteristics of economic burden studies 9
1.5 Quality assessment results 10
1.6 Data from additional costs studies 12
1.6.1 Costs for combined groups of patients 12
1.6.2 Costs from intervention studies 12
1.7 Economic burden analyses 12
1.7.1 Economic burden analyses of COPD 12

1.7.2 Economic burden analyses of AECOPD 13
1.7.3 Economic burden analyses of CB 13
1.7.4 Economic burden for combined groups of patients 13
1.8 Length of hospital stay 14
1.9 Disease burden 14
1.10 Eligible studies 18

1.1 Search strategy

Date of search: 2022.03.28

1.1.1 PubMed databases (2013/01/01-2022/03/28)

Search number	Search query	Results
#1	("Chronic Obstructive"[Title/Abstract] AND ("Lung"[Title/Abstract] OR "pulmonary"[Title/Abstract] OR "airway"[Title/Abstract] OR "airflow"[Title/AbPubstract]) AND "Disease"[Title/Abstract]) OR "airflow obstruction*"[Title/Abstract] OR "airway obstruction*"[Title/Abstract] OR "COPD"[Title/Abstract] OR "Chronic Bronchitis"[Title/Abstract] OR "Pulmonary Emphysema"[Title/Abstract] OR "Lung Emphysema"[Title/Abstract] OR "pulmonary disease, chronic obstructive"[MeSH Terms]	123,392
#2	("post tuberculosis"[Title/Abstract] OR "post TB"[Title/Abstract]) OR "post-TB"[Title/Abstract] OR "post-tuberculosis"[Title/Abstract]	315
#3	#1 OR #2	145,641
#4	"cost"[Title/Abstract] OR “costs"[Title/Abstract] OR “costing"[Title/Abstract] OR "affordabilit*"[Title/Abstract] OR "price"[Title/Abstract] OR "prices"[Title/Abstract] OR "pricing"[Title/Abstract] OR "expenditure*"[Title/Abstract] OR "out-of-pocket"[Title/Abstract] OR "expense*"[Title/Abstract] OR "payment"[Title/Abstract] OR "spend*"[Title/Abstract] OR "economic*"[Title/Abstract] OR "pharmacoeconomic*"[Title/Abstract] OR "socioeconomic burden"[Title/Abstract] OR "socio-economic burden"[Title/Abstract] OR "Consumption"[Title/Abstract] OR “financ*"[Title/Abstract] OR "costs and cost analysis"[MeSH Terms] OR "Economics, Pharmaceutical"[Mesh Terms] OR "Economics, Medical"[Mesh Terms] OR "Economics, Nursing"[Mesh Terms] OR "Economics, Hospital"[Mesh Terms]	1,513,844
#5	#3 AND #4	8,013
#6	#3 AND #4 AND ("2013/01/01"[Date - Publication] : "2022/03/28"[Date - Publication])	4,293
#7	(#6) NOT (Preprint[Publication Type] OR Comment[Publication Type] OR Editorial[Publication Type] OR Letter[Publication Type] OR review[Publication Type])	3,454
#8	(#6) AND Letter[Publication Type]	24
#9	(#6) AND review[Publication Type]	783
**Notes:** no [key word] field		

1.1.2 Embase (2013-2022)

Search number	Search query	Results

#1	'chronic obstructive lung disease'/exp OR 'lung emphysema'/de OR 'chronic bronchitis'/exp OR ('chronic obstructive':ti,ab,kw AND (lung:ti,ab,kw OR pulmonary:ti,ab,kw OR airway:ti,ab,kw OR airflow:ti,ab,kw) AND disease:ti,ab,kw) OR copd:ti,ab,kw OR 'airflow obstruction*':ti,ab,kw OR 'airway obstruction*':ti,ab,kw OR ‘chronic bronchitis’:ti,ab,kw OR ‘pulmonary emphysema’:ti,ab,kw OR ‘lung emphysema’:ti,ab,kw	238,980
#2	'post tb':ab,ti,kw OR 'post tuberculosis':ab,ti,kw OR 'post-tb':ab,ti,kw OR 'post-tuberculosis':ab,ti,kw	463
#3	#1 OR #2	239,313
#4	'health care cost'/exp OR 'health economics'/exp OR cost:ab,ti,kw OR costs:ab,ti,kw OR costing:ab,ti,kw OR affordabilit*:ab,ti,kw OR price:ab,ti,kw OR prices:ab,ti,kw OR pricing:ab,ti,kw OR expenditure*:ab,ti,kw OR 'out of pocket':ab,ti,kw OR expense*:ab,ti,kw OR payment:ab,ti,kw OR spend*:ab,ti,kw OR economic*:ab,ti,kw OR pharmacoeconomic*:ab,ti,kw OR 'socioeconomic burden':ab,ti,kw OR 'socio-economic burden':ab,ti,kw OR consumption:ab,ti,kw OR financ*:ab,ti,kw	2,369,608
#5	#3 AND #4	21,276
#6	#3 AND #4 AND [2013-2022]/py	12,767
#7	#6 NOT (preprint:it AND 'unpublished, non-peer reviewed':it OR editorial:it OR 'conference abstract':it OR letter:it OR review:it)	6,447
#8	#6 AND letter:it	199
#9	#6 AND review:it	1,398
**Notes:** could only limited to the year of publication but not the date of publication<br>Airflow limitation was excluded in the search terms, as it returned too broad results and not necessarily relevant with COPD.<br>Explode 'chronic bronchitis'<br>De-explode 'lung emphysema'		

1.1.3 Web of Science (2013.01.01 – 2022.03.28)

Search number	Search query	Results
**Core Collection**		
#1	TS=(("Chronic Obstructive" AND (Lung OR pulmonary OR airway OR airflow) AND Disease) OR COPD OR "airflow obstruction*" OR "airway obstruction*" OR “Chronic Bronchitis” OR “Pulmonary Emphysema” OR “Lung Emphysema”)	121,154
#2	TS=("post tuberculosis" OR "post TB" OR "post-TB" OR "post-tuberculosis")	249
#3	#1 OR #2	121,342
#4	TS=(cost OR costs OR costing OR affordabilit* OR price OR prices OR pricing OR expenditure* OR out-of-pocket OR expense* OR payment OR spend* OR economic* OR	3,573,960

	pharmacoeconomic* OR "socioeconomic burden" OR "socio-economic burden" OR Consumption OR financ*)	
#5	#3 AND #4	7,771
#6	#3 AND #4<br>Publication data: 2013-01-01 to 2022-03-28	4,705
#7	#6 NOT DT=(Editorial Material OR Meeting Abstract OR letter OR review)	3,457
#8	#6 AND DT=(letter)	17
#9	#6 AND DT=(review)	716
**Russian Science Citation Index**		
#1	TS=(("Chronic Obstructive" AND (Lung OR pulmonary OR airway OR airflow) AND Disease) OR COPD OR "airflow obstruction*" OR "airway obstruction*" OR “Chronic Bronchitis” OR “Pulmonary Emphysema” OR “Lung Emphysema”)	1,928
#2	TS=("post tuberculosis" OR "post TB" OR "post-TB" OR "post-tuberculosis")	15
#3	#1 OR #2	1,943
#4	TS=(cost OR costs OR costing OR affordabilit* OR price OR prices OR pricing OR expenditure* OR out-of-pocket OR expense* OR payment OR spend* OR economic* OR pharmacoeconomic* OR "socioeconomic burden" OR "socio-economic burden” OR Consumption OR financ*)	88,651
#5	#3 AND #4	114
#6	#3 AND #4<br>Publication data: 2013-01-01 to 2022-03-28	118
#7	#6 NOT DT=(Editorial Material OR Meeting Abstract OR letter OR review)	91
#8	#6 AND DT=(letter)	0
#9	#6 AND DT=(review)	14
**Web of Science SciELO Citation Index**		
#1	TS=(("Chronic Obstructive" AND (Lung OR pulmonary OR airway OR airflow) AND Disease) OR COPD OR "airflow obstruction*" OR "airway obstruction*" OR “Chronic Bronchitis” OR “Pulmonary Emphysema” OR “Lung Emphysema”)	1,647
#2	TS=("post tuberculosis" OR "post TB" OR "post-TB" OR "post-tuberculosis")	6
#3	#1 OR #2	1,653
#4	TS=(cost OR costs OR costing OR affordabilit* OR price OR prices OR pricing OR expenditure* OR out-of-pocket OR expense* OR payment OR spend* OR economic* OR pharmacoeconomic* OR "socioeconomic burden" OR "socio-economic burden” OR Consumption OR financ*)	94,973
#5	#3 AND #4	168
#6	#3 AND #4<br>Publication data: 2013-01-01 to 2022-03-28	83
#7	#6 NOT DT=(Editorial Material OR Meeting Abstract OR letter OR review)	83

#8	#6 AND DT=(letter)	0
#9	#6 AND DT=(review)	0
**Web of Science KCI-Korean Journal**		
#1	TS=(("Chronic Obstructive" AND (Lung OR pulmonary OR airway OR airflow) AND Disease) OR COPD OR "airflow obstruction*" OR "airway obstruction*" OR “Chronic Bronchitis” OR “Pulmonary Emphysema” OR “Lung Emphysema”)	1,429
#2	TS=("post tuberculosis" OR "post TB" OR "post-TB" OR "post-tuberculosis")	8
#3	#1 OR #2	1,433
#4	TS=(cost OR costs OR costing OR affordabilit* OR price OR prices OR pricing OR expenditure* OR out-of-pocket OR expense* OR payment OR spend* OR economic* OR pharmacoeconomic* OR "socioeconomic burden" OR "socio-economic burden” OR Consumption OR financ*)	238,746
#5	#3 AND #4	63
#6	#3 AND #4<br>Publication data: 2013-01-01 to 2022-03-28	40
#7	#6 NOT DT=(Editorial Material OR Meeting Abstract OR letter OR review)	40
#8	#6 AND DT=(letter)	0
#9	#6 AND DT=(review)	0
**Notes**: Meeting Abstract were also excluded		

1.1.4 EconLit via EBSCOhost (2013.01.01 – 2022.03.31)

S1	TI (("Chronic Obstructive" AND (Lung OR pulmonary OR airway OR airflow) AND Disease) OR COPD OR "airflow obstruction*" OR "airway obstruction*" OR “Chronic Bronchitis” OR “Pulmonary Emphysema” OR “Lung Emphysema”) OR AB (("Chronic Obstructive" AND (Lung OR pulmonary OR airway OR airflow) AND Disease) OR COPD OR "airflow obstruction*" OR "airway obstruction*" OR “Chronic Bronchitis” OR “Pulmonary Emphysema” OR “Lung Emphysema”)	81
S2	TI ("post tuberculosis" OR "post TB" OR "post-TB" OR "post-tuberculosis") OR AB ("post tuberculosis" OR "post TB" OR "post-TB" OR "post-tuberculosis")	0
S3	S1 OR S2	81
S4	TI (cost OR costs OR costing OR affordabilit* OR price OR prices OR pricing OR expenditure* OR out-of-pocket OR expense* OR payment OR spend* OR economic* OR pharmacoeconomic* OR "socioeconomic burden" OR "socio-economic burden” OR Consumption OR financ*) OR AB (cost OR costs OR costing OR affordabilit* OR price OR prices OR pricing OR expenditure* OR out-of-pocket OR expense* OR payment OR spend* OR economic* OR pharmacoeconomic* OR "socioeconomic burden" OR "socio-economic burden” OR Consumption OR financ*)	753,340

S5	S3 AND S4	64
S6	S3 AND S4<br>Published Date: 20130101-20220331	35

Limiters - Published Date: 20130101-20220331
Expanders - Apply related words; Apply equivalent subjects
Search modes - Boolean/Phrase
Notes: there might be some reviews which are not original studies (no filter to limit publication types)
The number of results is the same when using [Subject] or not
No [key word] field

1.1.5 CINAHL via EBSCOhost (2013.01.01 – 2022.03.31) – CINAHL subject headings

S1	(MH "Pulmonary Disease, Chronic Obstructive+") OR (MM "Bronchitis, Chronic") OR (MM "Emphysema") OR TI ("Chronic Obstructive" AND (Lung OR pulmonary OR airway OR airflow) AND Disease) OR COPD OR "airflow obstruction" OR "airway obstruction*" OR "Chronic Bronchitis" OR "Pulmonary Emphysema" OR "Lung Emphysema") OR AB ("Chronic Obstructive" AND (Lung OR pulmonary OR airway OR airflow) AND Disease) OR COPD OR "airflow obstruction" OR "airway obstruction*" OR "Chronic Bronchitis" OR "Pulmonary Emphysema" OR "Lung Emphysema")	16,124
S2	TI ("post tuberculosis" OR "post TB" OR "post-TB" OR "post-tuberculosis") OR AB ("post tuberculosis" OR "post TB" OR "post-TB" OR "post-tuberculosis")	18
S3	S1 OR S2	16,138
S4	(MH "Costs and Cost Analysis+") OR (MM "Economics, Pharmaceutical") OR TI (cost OR costs OR costing OR affordabilit* OR price OR prices OR pricing OR expenditure* OR out-of-pocket OR expense* OR payment OR spend* OR economic* OR pharmacoeconomic* OR "socioeconomic burden" OR "socio-economic burden" OR Consumption OR financ*) OR AB (cost OR costs OR costing OR affordabilit* OR price OR prices OR pricing OR expenditure* OR out-of-pocket OR expense* OR payment OR spend* OR economic* OR pharmacoeconomic* OR "socioeconomic burden" OR "socio-economic burden" OR Consumption OR financ*)	213,401
S5	S3 AND S4	1,045
S6	S3 AND S4<br>Published Date: 20130101-20220331	754
S7	S6 NOT (PT Editorial Material OR Letter OR Review)	616
S8	S6 AND (PT letter)	4
S9	S6 AND (PT review)	44

Limiters - Published Date: 20130101-20220331; Exclude MEDLINE records
Expanders - Apply related words; Apply equivalent subjects
Search modes - Boolean/Phrase
Notes: there is no [key word] field

1.1.6 Global Index Medicus databases (2013-2022) – indexed with MeSH terms

#1	tw:(("Chronic Obstructive" AND (lung OR pulmonary OR airway OR airflow) AND disease) OR “copd” OR "airflow obstruction" OR "airway obstruction*" OR “Chronic Bronchitis” OR “Pulmonary Emphysema” OR “Lung Emphysema”)	2,432
#2	(tw:("post tuberculosis" OR "post TB" OR "post-TB" OR "post-tuberculosis"))	30
#3	#1 OR #2<br>tw:((tw:(("Chronic Obstructive" AND (lung OR pulmonary OR airway OR airflow) AND disease) OR “copd” OR "airflow obstruction" OR "airflow obstructions" OR "airway obstruction*" OR “Chronic Bronchitis” OR “Pulmonary Emphysema” OR “Lung Emphysema”)) OR (tw:("post tuberculosis" OR "post TB" OR "post-TB" OR "post-tuberculosis")))	2,460
#4	tw:(cost OR costs OR costing OR affordabilit* OR price OR prices OR pricing OR expenditure* OR out-of-pocket OR expense* OR payment OR spend* OR economic* OR pharmacoeconomic* OR "socioeconomic burden" OR “socio-economic burden” OR Consumption OR financ*)	196.036
#5	#3 AND #4<br>(tw:(tw:((tw:(("Chronic Obstructive" AND (lung OR pulmonary OR airway OR airflow) AND disease) OR “copd” OR "airflow obstruction" OR "airflow obstructions" OR "airway obstruction*" OR “Chronic Bronchitis” OR “Pulmonary Emphysema” OR “Lung Emphysema”)) OR (tw:("post tuberculosis" OR "post TB" OR "post-TB" OR "post-tuberculosis"))))) AND (tw:(tw:(cost OR costs OR costing OR affordabilit* OR price OR prices OR pricing OR expenditure* OR out-of-pocket OR expense* OR payment OR spend* OR economic* OR pharmacoeconomic* OR "socioeconomic burden" OR “socio-economic burden” OR Consumption OR financ*)))	154
#6	#5<br>Limited to publication year between 2013 and 2022	56
**Notes**: searched by title, abstract, subject (tw)<br>There is no filter to limit publication types		

1.1.7 CENTRAL (The Cochrane Central Register of Controlled Trials) - via Cochrane library (2013-2022) – indexed with MeSH terms

#1	MeSH descriptor: [Pulmonary Disease, Chronic Obstructive] explode all trees	6,223
#2	(("Chronic Obstructive" AND (Lung OR pulmonary OR airway OR airflow) AND Disease) OR COPD OR "airflow obstruction*" OR "airway obstruction*" OR “Chronic Bronchitis” OR “Pulmonary Emphysema” OR “Lung Emphysema”):ti,ab,kw	24,157
#3	#1 OR #2	24,174

#4	("post tuberculosis" OR "post TB" OR "post-TB" OR "post-tuberculosis"):ti,ab,kw	24
#5	#3 OR #4	24,192
#6	MeSH descriptor: [Costs and Cost Analysis] explode all trees	11,322
#7	MeSH descriptor: [Economics, Medical] explode all trees	62
#8	MeSH descriptor: [Economics, Hospital] explode all trees	734
#9	MeSH descriptor: [Economics, Nursing] explode all trees	13
#10	MeSH descriptor: [Economics, Pharmaceutical] explode all trees	65
#11	(cost OR costs OR costing OR affordabilit* OR price OR prices OR pricing OR expenditure* OR out-of-pocket OR expense* OR payment OR spend* OR economic* OR pharmacoeconomic* OR "socioeconomic burden" OR "socio-economic burden" OR Consumption OR financ*):ti,ab,kw	156,441
#12	#6 OR #7 OR #8 OR #9 OR #10 OR #11	156,448
#13	#5 AND #12	2,266
#14	#5 AND #12<br>with Cochrane Library publication date from Jan 2013 to Mar 2022	1,698
#15	#5 AND #12<br>with Publication Year from 2013 to 2022, in Trials	1,268
#16	#5 AND #12<br>with Cochrane Library publication date from Jan 2013 to Mar 2022, in Cochrane Reviews	36
#17	#5 AND #12<br>with Cochrane Library publication date from Jan 2013 to Mar 2022, in Special Collections	0
**Notes:**<br>Content type: Cochrane Reviews, Cochrane Protocols, Trials, Clinical Answers, Editorials, Special Collections.		

1.1.8 Cochrane National Health Service Economic Evaluation Database (NHS EED) - via https://www.crd.york.ac.uk/CRDWeb/ (2013-2022) - indexed with MeSH terms

#1	(MeSH DESCRIPTOR Pulmonary Disease, Chronic Obstructive EXPLODE ALL TREES) IN NHSEED	151
#2	(((Chronic Obstructive) AND (Lung OR pulmonary OR airway OR airflow) AND Disease) OR COPD OR (airflow obstruction*) OR (airway obstruction*) OR (Chronic Bronchitis) OR (Pulmonary Emphysema) OR (Lung Emphysema)) IN NHSEED	275
#3	#1 OR #2	276
#4	((post tuberculosis) OR (post TB) OR (post-TB) OR (post-tuberculosis)) IN NHSEED	1
#5	#3 OR #4	277
#6	((MeSH DESCRIPTOR Costs and Cost Analysis EXPLODE ALL TREES) OR (MeSH DESCRIPTOR Economics, Medical EXPLODE ALL TREES) OR (MeSH DESCRIPTOR Economics, Hospital EXPLODE ALL TREES) OR (MeSH	15,030

	DESCRIPTOR Economics, Nursing EXPLODE ALL TREES) OR (MeSH DESCRIPTOR Economics, Pharmaceutical EXPLODE ALL TREES)) IN NHSEED	
#7	(cost OR costs OR costing OR affordabilit* OR price OR prices OR pricing OR expenditure* OR out-of-pocket OR expense* OR payment OR spend* OR economic* OR pharmacoeconomic* OR (socioeconomic burden) OR (socio-economic burden) OR Consumption OR financ*) IN NHSEED	17,613
#8	#6 OR #7	17,613
#9	#5 AND #8	277
#10	(#9) IN NHSEED FROM 2013 TO 2022	29
**Notes**: searched by any field; there is no filter to limit publication types		

1.2 Eligibility criteria

Table S1.2 Eligibility criteria

Inclusion criteria	Exclusion criteria
• Original studies reporting costs of COPD including chronic bronchitis and pulmonary emphysema or post-TB in LMICs<br>• Published between 1 January 2013 and 28 March 2022 (the date of the search)<br>• Any study design (retrospective, prospective, cohort, cross-sectional, case-control, etc.)<br>• Any language	• Other disease<br>• No costs on COPD or post-TB in LMICs<br>• Studies reporting costs of COPD or post-TB in HICs<br>• Pre-print paper<br>• Economic modelling studies without original data or original methods on the cost estimates<br>• No patient involved<br>• Protocols, reviews, editorials, letters to editors, commentaries, posters, abstracts presented at scientific conferences, and meeting proceedings

1.3 Quality assessment tool

The critical appraisal tool is drafted by adapting from JBI checklist for economic evaluations (1) and Consensus on Health Economic Criteria (CHEC) (2), together with consideration of Global Health Cost Consortium (GHCC) Reference Case for Estimating the Costs of Global Health Services and Interventions (3) and Consolidated Health Economic Evaluation Reporting Standards (CHEERS) (4).

There are 15 items in total. Sub-items a), b), and c) indicate the points that need to be considered when assessing the compliance with each quality criterion.

Q1 Is there a well-defined question/objective?
- a) Is the objective/question of the study clearly stated?
- b) Was the study placed in a particular decision-making context?

Q2 Is the study population clearly described?
- a) Are study population characteristics described?
- b) Is the place where study populated were recruited or from stated?

Q3 Is the perspective of costs clearly stated?
- a) Such as patient, health provider, health payer, and societal etc.

Q4 Is there comprehensive description of alternatives (i.e., intervention and/or comparators)? (**If applicable**)

a) This question is only applicable to interventional studies.

Q5 Is clinical effectiveness measured in this study or a related study (a publication by the same group, or a citation to another analysis)? If yes, has clinical effectiveness been well established? **(If applicable)**

a) The effectiveness estimate in the evaluation does not need to be derived from the same study as the resource use/cost estimate.

b) Clinical effectiveness is considered to be well established if it's from randomized-control study or cluster-randomized study, and not well established if it's from observational studies.

Q6 Is there a detailed description of the measures used for diagnosis of the disease?

a) The answer is "Yes" if the study stated the diseases of interest were diagnosed or identified according to GOLD guideline, ICD-9, ICD-10, or other disease classification system.

Q7 Is there a detailed description of the measures used for costs?

a) Detailed description of the measures used for costs and how it justifies them.

b) If it's a lump sum cost, do they list what that lump sum includes?

Q8 Is there discussion about any limitations associated with the measures used and concerns about the accuracy of measurement?

Q9 If this is a costing study of some patients, then does the study state the year in which the costs were collected or converted to?

Q10 Are costs adjusted for differential timing? **(If applicable)**

a) Does the study identify and justify the discount rate used?

b) Dose the study identify and justify the time frame over which they study was conducted?

Q11 Is there any incremental analysis of costs and consequences? **(If applicable)**

a) This question is applicable for cost-effectiveness or cost-utility analysis studies, etc.

b) Dose the study report changes in costs and benefits – marginal shifts in resources from the comparator to the intervention?

Q12 Were sensitivity analyses conducted to investigate uncertainty in estimates of cost or consequences? **(If applicable)**

a) This question is applicable for modelling studies, or studies with simulations or calculations into the future, but not applicable for costing studies. Although sometimes, authors might state uncertainty analysis in costing studies. But we do not assess this for costing studies.

b) Dose the study describe how the study findings vary with changes in key variables.

Q13 Does the article indicate that there is no potential conflict of interest of study researcher(s) and funder(s)?

a) Is there no role of funders in the study design, research, and result interpreting, as well as no conflicts among authors/researchers?

Possible answers for each criterion are "yes", "no", "unclear", "not applicable".

1.4 Characteristics of economic burden studies

Table S1.4 Characteristics of economic burden studies

Literature characteristics	Category	Number of studies*	Percentage
Publication year	2013	1	1.5%
	2014	1	1.5%
	2015	4	6.2%
	2016	10	15.4%

Literature characteristics	Category	Number of studies*	Percentage
	2017	9	13.8%
	2018	10	15.4%
	2019	8	12.3%
	2020	7	10.8%
	2021	14	21.5%
	2022	1	1.5%
Geographical region	Across the globe	1	1.5%
	Africa	1	1.5%
	Asia	40	61.5%
	Eastern Europe	15	23.1%
	Latin America	7	10.8%
	Middle East and North Africa	1	1.5%
World Bank income group	Low and middle income	1	1.5%
	Low income	1	1.5%
	Lower middle income	17	26.2%
	Upper middle income	49	75.4%
Country	China	24	36.9%
	Russia	9	13.8%
	Iran	6	9.2%
	India	4	6.2%
	Brazil	4	6.2%
	Argentina	3	4.6%
	Bulgaria	3	4.6%
	Peru	3	4.6%
	Thailand	3	4.6%
	Turkey	3	4.6%
	Other	9	13.8%
Disease	COPD	51	78.5%
	Chronic bronchitis	13	20.0%
	Bronchitis and emphysema	2	3.1%
	AECOPD	1	1.5%
	COPD/asthma	1	1.5%
	COPD associated malnutrition	1	1.5%
Attribution	Air pollution	29	44.6%
	Smoking	17	26.2%
	Second-hand smoking	1	1.5%
	COPD-associated malnutrition	1	1.5%
	No attribution or not applicable	24	36.9%

*Total number of economic burden studies is 65, which is the denominator for percentage. Six studies (5–10) reported economic burden with and without attribution to certain factors, three studies (11–13) across countries from multiple income groups, and four studies (14–17) across 2 diseases. Therefore, the sum of the studies across income groups, countries, diseases and attribution is more than 65.

1.5 Quality assessment results

Table S1.5 Quality assessment results of cost analysis studies (n (percentage))

Question	No	Yes	Unclear	Not applicable	Yes, only for funders	Yes, only for authors	Yes, both for funders and authors
**Cost analysis studies (n=128)**							
Q1 Research question	0 (0%)	123 (96.1%)	5 (3.9%)	0 (0%)	-	-	-
Q2 Cost perspective	78 (60.9%)	36 (28.1%)	14 (10.9%)	0 (0%)	-	-	-

Question	No	Yes	Unclear	Not applicable	Yes, only for funders	Yes, only for authors	Yes, both for funders and authors
Q3 Study population	19 (14.8%)	98 (76.6%)	11 (8.6%)	0 (0%)	-	-	-
Q4 Description of alternatives	3 (2.3%)	47 (36.7%)	1 (0.8%)	77 (60.2%)	-	-	-
Q5 Clinical effectiveness	29 (22.7%)	11 (8.6%)	10 (7.8%)	78 (60.9%)	-	-	-
Q6 Disease diagnosis	42 (32.8%)	77 (60.2%)	9 (7.0%)	0 (0%)	-	-	-
Q7 Cost measures	32 (25.0%)	66 (51.6%)	30 (23.4%)	0 (0%)	-	-	-
Q8 Limitation	45 (35.2%)	81 (63.3%)	2 (1.6%)	0 (0%)	-	-	-
Q9 Year of costs	37 (28.9%)	87 (68.0%)	4 (3.1%)	0 (0%)	-	-	-
Q10 Differential timing	1 (0.8%)	8 (6.3%)	1 (0.8%)	118 (92.2%)	-	-	-
Q11 Incremental analysis	5 (3.9%)	11 (8.6%)	0 (0%)	112 (87.5%)	-	-	-
Q12 Sensitivity analysis	7 (5.5%)	10 (7.8%)	1 (0.85)	110 (85.9%)	-	-	-
Q13 Conflict of interest	36 (28.1%)	-	1 (0.8%)	0 (0%)	2 (1.6%)	57 (44.5%)	32 (25.0%)
**Economic burden studies (n=65)**							
Q1 Research question	0 (0%)	62 (95.4%)	3 (4.6%)	0 (0%)	-	-	-
Q2 Cost perspective	21 (32.3%)	26 (40.0%)	18 (27.7%)	0 (0%)	-	-	-
Q3 Study population	14 (21.5%)	43 (66.2%)	8 (12.3%)	0 (0%)	-	-	-
Q4 Description of alternatives	1 (1.5%)	7 (10.8%)	0 (0%)	57 (87.7%)	-	-	-
Q5 Clinical effectiveness	5 (7.7%)	1 (1.5%)	0 (0%)	59 (90.8%)	-	-	-
Q6 Disease diagnosis	41 (63.1%)	20 (30.8%)	3 (4.6%)	0 (0%)	-	-	-
Q7 Cost measures	10 (15.4)	49 (75.4%)	6 (9.2%)	0 (0%)	-	-	-
Q8 Limitation	13 (20.0%)	47 (72.3%)	5 (7.7%)	0 (0%)	-	-	-
Q9 Year of costs	12 (18.5%)	50 (76.9%)	3 (4.6%)	0 (0%)	-	-	-
Q10 Differential timing	21 (32.3%)	19 (29.2)	1 (1.5%)	24 (36.9%)	-	-	-
Q11 Incremental analysis	1 (1.5%)	3 (4.6%)	1 (1.5%)	60 (92.3%)	-	-	-
Q12 Sensitivity analysis	30 (46.2%)	26 (40.0%)	2 (3.1%)	7 (10.8%)	-	-	-
Q13 Conflict of interest	22 (33.8%)	-	1 (1.5%)	0 (0%)	0 (0%)	25 (38.5%)	14 (21.5%)

1.6 Data from additional costs studies

1.6.1 Costs for combined groups of patients

Table S2.6 (**Supplementary Material 2**) shows costs and length of hospital stay (LOS) of diseases in studies that presented data without stratification for COPD, AECOPD and CB specifically. Peng et al. estimated hospitalization costs of $2,694 per COPD and/or AECOPD patient (18) and Altawalbeh et al. reported $2,085 per patient in population with diagnosis of asthma-COPD overlap (19). A study in Uganda showed that the medication costs per outpatient visit ranged from $0.04 in hospital to $0.61 in health center of level III for patients with COPD and/or asthma (20).

1.6.2 Costs from intervention studies

Table S2.7 (**Supplementary Material 2**) presents the costs and LOS of COPD, AECOPD and CB from intervention studies. Costs and LOS varied by different interventions. For example, Qiu and colleagues conducted an economic evaluation indicating the mean hospitalization costs of COPD were $892 before 23-valent pneumococcal polysaccharide vaccination and $308 after vaccination (21). Results from Lin and colleagues showed that hospitalization costs of AECOPD patients decreased from $2,441 per patient with pulmonary infection control window guided treatment to $1,812 per patient with procalcitonin kinetics guided treatment and LOS was reduced from 11.5 to 8.7 days (22).

1.7 Economic burden analyses

1.7.1 Economic burden analyses of COPD

Table S3.1 (**Supplementary Material 3**) shows the economic burden of COPD by country. The direct economic burden of hospitalization was estimated to be $3.9 million in Bulgaria (0.074% of health expenditures, or HE), $111.6 million in Iran (0.14% of HE), and $1,404.3 million in Thailand (9.8% of HE), of which $31.8 million (0.22% of HE) were from COPD patients with a TB diagnosis; the burden attributable to smoking was $18 million in Iran (0.023% of HE); the burden due to PM_{10} pollution was $515.6 million in China (0.095% of HE).

Direct medical cost burden of COPD was estimated as $97.7 million from the perspective of the healthcare system in Russia (0.12% of HE), $108.1 million in Paraguay (4.8% of HE), $285.4 million in Turkey (1.1% of HE) and $3,040.9 million in China (0.43% of HE), while the costs attributable to smoking ranged from $15.5 million in Honduras (0.86% of HE) to 4,004.2 million in Brazil (2.9% of HE). Moreover, the direct cost burden was $103.4 million in Russia (0.13% of HE) and $294.3 million in Peru (3.1% of HE), and the burden attributable to smoking was $10.5 million in Russia (0.13% of HE), $226.1 million in Peru (2.4% of HE), and $401.9 million in Vietnam (4.3% of HE).

There were some studies estimating the economic burden of premature mortality due to COPD. It was $40.8 million in Iran (where the GDP per capita was $12,816 and the population was 80 million) and $2,705.2 million in Russia (where the GDP per capita was $10,139 and the population was 144.3 million); the burden attributable to $PM_{2.5}$ pollution was $717.3 million in Nigeria (where the GDP per capita was $3,204 and the population was 184 million) and $6,903.9 million in Thailand (where the GDP per capita was $6,487 and the population was 70.6 million); the burden attributable to smoking ranged from $102.4 million in Vietnam (where the GDP per capita was $2,379 and the population was 88.3 million) to $515.7 million in Russia (where the GDP per capita

was $10,139 and the population was 144 million). Disease burden in terms of mortality and morbidity are described in the **Supplementary Section 3-II**.

Table S3.2 (**Supplementary Material 3**) also presents the economic burden of COPD at subnational and regional level. For example, Huang and colleagues estimated economic burden of COPD due to premature mortality to be $4,443.1 million in the Ningbo and the Yangtze River Delta region of China, of which, $324.4 million were attributable to unsafe ozone concentrations (23). Bayat et al. estimated that excess costs attributable to air pollution would be $25.9 million if $PM_{2.5}$ increases to 35 μg/m^3 while the economic benefits would be $40.3 million and $110.9 million if $PM_{2.5}$ decreases to 35 μg/m^3 and to 25 μg/m^3, respectively (24). The burden of mortality due to $PM_{2.5}$ was estimated to be $17.8 billion, $1562.4 billion and $3672.9 billion in low-income, lower middle-income and upper middle-income countries, respectively (13).

1.7.2 Economic burden analyses of AECOPD

There was one study reporting the economic burden of AECOPD specifically (25). The authors estimated that the hospitalization cost burden attributable to air pollution in Guangdong, China ranged from $15.8 million in 2013 to $11.8 million in 2017 (**Supplementary Material 3, Table S3.4**).

1.7.3 Economic burden analyses of CB

The mortality cost burden of CB attributable to $PM_{2.5}$ in China was $4,564.2 million in 2017 in Gansu province and $24.3 million in 2016 in Ji'nan city, while the national burden attributable to PM_{10} was $28,064.3 million in 2014 (where the GDP per capita was $8,188, and the population was 1.39 billion). The economic burden of premature mortality due to air pollution between 1996 and 2015 in Bulgaria and Romania was estimated to be $36,837 (where the GDP per capita was $6,770, and the population (2015) was 7.2 million) and $81,525 (where the GDP per capita was $7,733, and the population (2015) was 19.8 million), respectively, due in part to the smaller populations of those countries. The disability cost burden due to PM_{10} in India in the year of 2015 was calculated as $1,277.5 in Mumbai, and $1,923.3 million in Delhi (**Supplementary Material 3, Table S3.3**).

The societal burden in China due to $PM_{2.5}$ was estimated as $2,604.3 million among population aged <14 years and $2,689.5 million among those ≥15 in Xi'an city in 2016, while in Beijing the burden attributable to PM_{10} ranged from $14.0 billion in 2008 to $15.2 billion in 2012.

1.7.4 Economic burden for combined groups of patients

As shown in **Table S3.4** (**Supplementary Material 3**), the economic burden of hospitalization costs of bronchitis and emphysema among the population aged 35 or older in Thailand from 2007 to 2014 was $33 million (0.23% of HE) with $13.6 million from those with TB diagnosis (0.095% of HE) (17). In Indonesia, the direct medical cost burden of bronchitis and emphysema due to smoking in 2015 was $9.7 million (0.036% of HE) among the male population, but much less among the female population $16,152 because women have much fewer smoking-attributable cases (26,731 versus 1,071). The total economic burden of COPD-associated malnutrition was $17.6 billion in China in 2011 (4.5% of HE), with $13 billion attributed to the population aged 60 and older (3.3% of HE), $4.5 billion attributed to people aged 15-59 years (1.1% of HE) and $6.1 million attributed to people aged <15 (0.0016% of HE). Mongolia bore $3.1 million in public sector spending due to COPD/asthma burden in 2013 (0.68% of HE).

1.8 Length of hospital stay

The mean LOS among COPD patients ranged from 0.5 days among patients with mild to moderate disease to 15 days per patient (with disease severity not stated) (**Supplementary Material 2, Table S2.1**). By World Bank income groups, the range of LOS was 5.6-11.8 in lower MICs and 0.5-15 among upper MICs. Among AECOPD patients, the LOS ranged from 4.2-9.3 and 6.9-18.5 days in lower and upper MICs, respectively (**Supplementary Material 2, Table S2.3**).

One study by Zhu et al. indicated average LOS of 26 days among CB patients with treatment of cephalosporin combined with herba houttuyniae and 32.4 days among those with treatment of cephalosporin alone (26).

1.9 Disease burden

Table S3.5 (**Supplementary Material 3**) displays the disease burden of COPD per country. The annual mortality burden of COPD ranged from 1,174 deaths in Paraguay in a population of 2.26 million to 507,705 deaths in a Chinese region with a population of 4 million people with COPD hospitalizations. The number of deaths per year attributable to smoking ranged from 432 in a Costa Rican population of 4.8 million, 461 in Honduras with a population of 8.1 million to 31,120 in Brazil with a population 207.8 million population. The burden due to air pollution was 4,999 deaths between 1996 and 2016 in Thailand with a population of 43.8 million population and 105,000 deaths in 2016 in 31 Chinese provinces totaling a population of 1,308 million. Besides, Thailand bore a morbidity burden of COPD attributable to $PM_{2.5}$ of 31,090 DALYs and China bore 5,061 DALYs attributable to PM_{10}. **Supplementary Material 3, Table S3.6** also presents the disease burden of COPD at a subnational level.

Table S3.7 (**Supplementary Material 3**) shows the disease burden of CB. The annual morbidity burden in DALYs attributable to PM_{10} was 235,832.6 in Mumbai, a city of 18.84 million population; 355,052.9 in Delhi, a city of 17.45 million population in 2015; and 4,350,249 in 190 Chinese cities of 869.7 million population in 2014. The mortality burden of COPD-associated malnutrition ranged from 12 deaths among population aged 0-14 to 183,425 deaths among those aged 60+, while DALYs ranged from 458 to 975,270 (**Supplementary Material 3, Table S3.8**).

References for sections 1.3 - 1.9:

1. Joanna Briggs Institute. Checklist for Economic Evaluations. Critical Appraisal tools for use in JBI Systematic reviews [Internet]. 2020. Available from: jbi.global

2. Evers S, Goossens M, De Vet H, Van Tulder M, Ament A. Criteria list for assessment of methodological quality of economic evaluations: Consensus on Health Economic Criteria. Int J Technol Assess Health Care. 2005;21(2):240–5.

3. Vassall A, Sweeney S, Kahn J, Gomez Guillen G, Bollinger L, Marseille E, et al. Reference case for estimating the costs of global health services and interventions. 2017;

4. Husereau D, Drummond M, Petrou S, Carswell C, Moher D, Greenberg D, et al. Consolidated health economic evaluation reporting standards (CHEERS)—explanation and elaboration: a report of the ISPOR health economic evaluation publication guidelines good reporting practices task force. Value Heal. 2013;16(2):231–50.

5. Alcaraz A, Caporale J, Bardach A, Augustovski F, Pichon-Riviere A. Burden of disease attributable to tobacco use in Argentina and potential impact of price increases through taxes. Rev Panam SALUD PUBLICA-PAN Am J PUBLIC Heal. 2016;40(4):204-212 WE-Social Science Citation Index (S.

6. Rezaei S, Karami Matin B, Hajizadeh M, Bazyar M, Akbari Sari A. Economic Burden of Smoking in Iran: A Prevalence-Based Annual Cost Approach. Asian Pac J Cancer Prev [Internet]. 2017;18(10):2867–73. Available from: https://www.embase.com/search/results?subaction=viewrecord&id=L630217795&from=export U2 - L630217795

7. Cai L, Cui W, He J, Wu X. The economic burden of smoking and secondhand smoke exposure in rural South-West China. J Asthma [Internet]. 2014;51(5):515–21. Available from: https://www.embase.com/search/results?subaction=viewrecord&id=L373121055&from=export U2 - L373121055

8. Bardach A, Cañete F, Sequera VG, Palacios A, Alcaraz A, Rodríguez B, et al. Burden of disease attributable to tobacco use in Paraguay, and potential health and financial impact of increasing prices through taxing. Carga Enferm atribuible al uso del Tab en paraguay y potencial impacto Sanit y económico del aumento del precio a través impuestos [Internet]. 2018;35(4):599–609. Available from: https://www.embase.com/search/results?subaction=viewrecord&id=L2001604525&from=export U2 - L2001604525

9. Bardach AE, Caporale JE, Alcaraz A, Augustovski F, Huayanay-Falconí L, Loza-Munarriz C, et al. Burden of smoking-related disease and potential impact of cigarette price increase in Peru. Carga Enferm por Tab e impacto potencial del incremento precios cigarrillos en el Perú [Internet]. 2016;33(4):651–61. Available from: https://www.embase.com/search/results?subaction=viewrecord&id=L614479894&from=export U2 - L614479894

10. Sapunova ID, Kontsevaya A V, Myrzamatova AO, Mukaneeva DK, Khudyakov MB, Ipatov P V, et al. Economic damage from smoking associated with four groups of chronic non-communicable diseases in the Russian Federation in 2016. Экономический ущерб от курения, ассоциированный с четырьмя группами хронических неинфекционных заболеваний в Российской Федерации в 2016 году [Internet]. 2019;18(6):6–12. Available from: https://www.embase.com/search/results?subaction=viewrecord&id=L2005010347&from=export U2 - L2005010347

11. Pichon-Riviere A, Bardach A, Augustovski F, Alcaraz A, Reynales-Shigematsu LM, Teixeira Pinto M, et al. Impacto económico del tabaquismo en los sistemas de salud de América Latina: un estudio en siete países y su extrapolación a nivel regional. Rev Panam Salud Pública [Internet]. 2016;40(4):213–21. Available from: https://search.ebscohost.com/login.aspx?direct=true&db=cin20&AN=120443551&authtype=shib&site=ehost-live

12. Pichon-Riviere A, Alcaraz A, Palacios A, Rodríguez B, Reynales-Shigematsu LM, Pinto M, et al. The health and economic burden of smoking in 12 Latin American countries and the potential effect of increasing tobacco taxes: an economic modelling study. Lancet Glob Heal [Internet]. 2020;8(10):e1282–94. Available from: https://www.embase.com/search/results?subaction=viewrecord&id=L2007882946&from=

export U2 - L2007882946

13. Yin H, Brauer M, Zhang JJ, Cai W, Navrud S, Burnett R, et al. Population ageing and deaths attributable to ambient PM2·5 pollution: a global analysis of economic cost. Lancet Planet Heal [Internet]. 2021;5(6):e356–67. Available from: https://www.embase.com/search/results?subaction=viewrecord&id=L2012929470&from=export U2 - L2012929470

14. Kristina SA, Endarti D, Wiedyaningsih C, Fahamsya A, Faizah N. Health Care Cost of Noncommunicable Diseases Related to Smoking in Indonesia, 2015. Asia-Pacific J Public Heal [Internet]. 2018;30(1):29–35. Available from: https://search.ebscohost.com/login.aspx?direct=true&db=cin20&AN=128060503&authtype=shib&site=ehost-live

15. Maji KJ, Arora M, Dikshit AK. Burden of disease attributed to ambient PM2.5 and PM10 exposure in 190 cities in China. Env Sci Pollut Res Int [Internet]. 2017;24(12):11559–72. Available from: https://www.embase.com/search/results?subaction=viewrecord&id=L616925778&from=export U2 - L616925778

16. Maji KJ, Ye WF, Arora M, Shiva Nagendra SM. PM2.5-related health and economic loss assessment for 338 Chinese cities. Environ Int [Internet]. 2018;121:392–403. Available from: https://www.embase.com/search/results?subaction=viewrecord&id=L2001116521&from=export U2 - L2001116521

17. Patanavanich R, Aekplakorn W, Suriyawongpaisal P. Trend analysis of smoking-attributable hospitalizations in Thailand, 2007-2014. Tob Induc Dis [Internet]. 2018;16:1–10. Available from: https://search.ebscohost.com/login.aspx?direct=true&db=cin20&AN=135261923&authtype=shib&site=ehost-live

18. Peng X, Yu J, Zhang W, Li Y, Wu M, Xu T, et al. Drug usage and costs of inpatients with chronic obstructive pulmonary disease in Karamay Central Hospital in 2014. Chin J Evid-Based Med [Internet]. 2017;17(8):888–91. Available from: https://www.embase.com/search/results?subaction=viewrecord&id=L623184481&from=export U2 - L623184481

19. Altawalbeh SM, Hijazi B, Kufoof L, Basheti IA. Health expenditures of asthma-COPD overlap in Northern Jordan. PLoS One. 2021;16(9):e0257566.

20. SN S, Sweeney S, Seeley J, Biraro S, Mutungi G, Munderi P, et al. The health system burden of chronic disease care: an estimation of provider costs of selected chronic diseases in Uganda. Trop Med Int Health [Internet]. 2015;20(6):781–90. Available from: https://pubmed.ncbi.nlm.nih.gov/25707376/

21. YP Q, Zhao K, Li X, LW S, WD G, XR Q, et al. [Health economic evaluation of a 23 value pneumococcal polysaccharide vaccination pilot programme among elderly chronic obstructive pulmonary disease patients in China]. Zhonghua Yu Fang Yi Xue Za Zhi [Internet]. 2016;50(12):1074–8. Available from: https://pubmed.ncbi.nlm.nih.gov/28057111/

22. Lin SH, He YP, Lian JJ, Chu CK. Procalcitonin kinetics to guide sequential invasive-noninvasive mechanical ventilation weaning in patients with acute exacerbation of chronic

obstructive pulmonary disease and respiratory failure: procalcitonin’s adjunct role. Libyan J Med [Internet]. 2021;16(1):1961382. Available from: https://www.cochranelibrary.com/central/doi/10.1002/central/CN-02304486/full

23. Huang J, Li G, Xu G, Qian X, Zhao Y, Pan X, et al. The burden of ozone pollution on years of life lost from chronic obstructive pulmonary disease in a city of Yangtze River Delta, China. Environ Pollut [Internet]. 2018;242:1266–73. Available from: https://www.embase.com/search/results?subaction=viewrecord&id=L2001028344&from=export U2 - L2001028344

24. Bayat R, Ashrafi K, Shafiepour Motlagh M, Hassanvand MS, Daroudi R, Fink G, et al. Health impact and related cost of ambient air pollution in Tehran. Environ Res [Internet]. 2019;176. Available from: https://www.embase.com/search/results?subaction=viewrecord&id=L2002160721&from=export U2 - L2002160721

25. Wang Z, Zhou Y, Zhang Y, Huang X, Duan X, Chen D, et al. Association of change in air quality with hospital admission for acute exacerbation of chronic obstructive pulmonary disease in Guangdong, China: A province-wide ecological study. Ecotoxicol Environ Saf [Internet]. 2021;208. Available from: https://www.embase.com/search/results?subaction=viewrecord&id=L2008568239&from=export U2 - L2008568239

26. Zhu YF, Gao Z, Zhu M, Cai S, Zhou H. Clinical effect of cephalosporin combined with herba houttuyniae on senile chronic bronchitis. Prog Mod Biomed [Internet]. 2015;15:6919-6921. Available from: https://www.cochranelibrary.com/central/doi/10.1002/central/CN-02190280/full

1.10 Eligible studies

Below are the studies included in the review.

1. Sari AA, Rezaei S, Arab M, B KM, Majdzadeh R. Does smoking status affect cost of hospitalization? Evidence from three main diseases associated with smoking in Iran. Med J Islam Repub Iran [Internet]. 2017;31:63. Available from: https://pubmed.ncbi.nlm.nih.gov/29445692/
2. Abdulsalim S, Unnikrishnan MK, Manu MK, Alsahali S, Alrasheedy AA, Martin AP, et al. Impact of a Clinical Pharmacist Intervention on Medicine Costs in Patients with Chronic Obstructive Pulmonary Disease in India. Pharmacoeconomics - open [Internet]. 2020;4(2):331-342. Available from: https://www.cochranelibrary.com/central/doi/10.1002/central/CN-02130671/full
3. Akramova EG, Khamitova RY, Bakirov Эндже Гамировна RSZA, Хамитова РЯ, Бакиров РС. The clinical economic analysis of hospital treatment of chronic obstructive disease of lungs FT Клинико-экономический анализ стационарного лечения хронической обструктивной болезни легких. Zdr Ross Fed S1 Здравоохранение Российской Федерации. 2014;58(4):41–6.
4. Alcaraz A, Caporale J, Bardach A, Augustovski F, Pichon-Riviere A. Burden of disease attributable to tobacco use in Argentina and potential impact of price increases through taxes. Rev Panam SALUD PUBLICA-PAN Am J PUBLIC Heal. 2016;40(4):204-212 WE-Social Science Citation Index (S.
5. Altaf M, AM Z, Nazneen F, Kareemulla S, SA A, NM A, et al. Cost-effectiveness analysis of three different combinations of inhalers for severe and very severe chronic obstructive pulmonary disease patients at a tertiary care teaching hospital of South India. Perspect Clin Res [Internet]. 2015;6(3):150–8. Available from: https://pubmed.ncbi.nlm.nih.gov/26229751/
6. Altawalbeh SM, Hijazi B, Kufoof L, Basheti IA. Health expenditures of asthma-COPD overlap in Northern Jordan. PLoS One. 2021;16(9):e0257566.
7. Artyukhov IP, Arshukova IL, Dobretsova EA, Shulmin A V. Mortality and economic burden of krasnoyarsk region, Russia, caused by regular tobacco usage. Int J COPD [Internet]. 2016;11(1):351–5. Available from: https://www.embase.com/search/results?subaction=viewrecord&id=L608595511&from=export U2 - L608595511
8. Artyukhov IP, Shulmin A V, Dobretsova EA, Arshukova И.П. ILZA, Шульмин АВ, Добрецова ЕА, et al. The evaluation of medical demographic losses and economical costs conditioned by chronic obstructive disease of lungs exemplified by the Krasnoyarsk region FT Оценка медико-демографических потерь и экономических затрат, обусловлен. Zdr Ross Fed S1 Здравоохранение Российской Федерации. 2015;59(5):32–7.
9. Astaf'ev A V, Styrt EA, Sinopal'nikov AI. Infectious exacerbation of chronic obstructive pulmonary disease: prospects for high-dose levofloxacin therapy. Klin Med (Mosk) [Internet]. 2013;91(3):44-50. Available from: https://www.cochranelibrary.com/central/doi/10.1002/central/CN-00864043/full
10. Aunan K, Alnes LWH, Berger J, Dong ZQ, Ma LY, Mestl HES, et al. Upgrading to cleaner household stoves and reducing chronic obstructive pulmonary disease among women in rural China - A cost-benefit analysis. ENERGY Sustain Dev. 2013;17(5):489–96.
11. Balunov PA, Khitrov П.А. ANZБ, Хитров АН. Pharmacoeconomic aspects of the use of bronchodilators in the management of COPD: real clinical practice FT Фармакоэкономические аспекты применения бронхолитиков в терапии ХОБЛ: реальная клиническая практика. Med Counc S1 Медицинский совет. 2018;(21):96–104.
12. Bao H, Wang J, Zhou D, Han Z, Zhang Y, Su L, et al. Community Physician-Guided Long-Term Domiciliary Oxygen Therapy Combined With Conventional Therapy in Stage IV COPD Patients. Rehabil Nurs [Internet]. 2017;42(5):268–73. Available from: https://www.embase.com/search/results?subaction=viewrecord&id=L621324290&from=export U2 - L621324290
13. Bardach AE, Caporale JE, Alcaraz A, Augustovski F, Huayanay-Falconí L, Loza-Munarriz C, et al. Burden of smoking-related disease and potential impact of cigarette price increase in Peru. Carga Enferm por Tab e impacto potencial del incremento precios cigarrillos en el Perú [Internet]. 2016;33(4):651–61. Available from: https://www.embase.com/search/results?subaction=viewrecord&id=L614479894&from=export U2 - L614479894
14. Bardach A, Cañete F, Sequera VG, Palacios A, Alcaraz A, Rodríguez B, et al. Burden of disease attributable to tobacco use in Paraguay, and potential health and financial impact of increasing prices through taxing. Carga Enferm atribuible al uso del Tab en paraguay y potencial impacto Sanit y económico del aumento del precio a través impuestos [Internet]. 2018;35(4):599–609. Available from: https://www.embase.com/search/results?subaction=viewrecord&id=L2001604525&from=export U2 - L2001604525
15. Bayat R, Ashrafi K, Shafiepour Motlagh M, Hassanvand MS, Daroudi R, Fink G, et al. Health impact and related cost of ambient air pollution in Tehran. Environ Res [Internet]. 2019;176. Available from: https://www.embase.com/search/results?subaction=viewrecord&id=L2002160721&from=export U2 -

L2002160721
16. Bundhamcharoen K, Aungkulanon S, Makka N, Shibuya K. Economic burden from smoking-related diseases in Thailand. Tob Control [Internet]. 2016;25(5):532–7. Available from: https://www.embase.com/search/results?subaction=viewrecord&id=L619912535&from=export U2 - L619912535
17. Cai L, Cui W, He J, Wu X. The economic burden of smoking and secondhand smoke exposure in rural South-West China. J Asthma [Internet]. 2014;51(5):515–21. Available from: https://www.embase.com/search/results?subaction=viewrecord&id=L373121055&from=export U2 - L373121055
18. Chen R, Gao Y, Wang H, Shang H, Xuan J. Association between adherence to maintenance medication in patients with COPD and acute exacerbation occurrence and cost in china: A retrospective cohort database study. Int J COPD [Internet]. 2020;15:963–71. Available from: https://www.embase.com/search/results?subaction=viewrecord&id=L2004324701&from=export U2 - L2004324701
19. Chen X, Wang N, Chen Y, Xiao T, Fu C, Xu B. Costs of chronic obstructive pulmonary disease in urban areas of China: a cross-sectional study in four cities. Int J Chron Obstruct Pulmon Dis [Internet]. 2016;11:2625–32. Available from: https://pubmed.ncbi.nlm.nih.gov/27799761/
20. Chen Y, Liu Y, Zhang J, Yao W, Yang J, Li F, et al. Comparison of the clinical outcomes between nebulized and systemic corticosteroids in the treatment of acute exacerbation of copd in China (Contain study): A post hoc analysis. Int J COPD [Internet]. 2020;15:2343–53. Available from: https://www.embase.com/search/results?subaction=viewrecord&id=L2005171114&from=export U2 - L2005171114
21. Cui Y, Zhan Z, Ma Y, Huang K, Liang C, Mao X, et al. Clinical and economic burden of comorbid coronary artery disease in patients with acute exacerbation of chronic obstructive pulmonary disease: sex differences in a nationwide cohort study. Respir Res [Internet]. 2022;23(1). Available from: https://www.embase.com/search/results?subaction=viewrecord&id=L2015074191&from=export U2 - L2015074191
22. Cui Y, Zhan Z, Zeng Z, Huang K, Liang C, Mao X, et al. Blood Eosinophils and Clinical Outcomes in Patients With Acute Exacerbation of Chronic Obstructive Pulmonary Disease: A Propensity Score Matching Analysis of Real-World Data in China. Front Med [Internet]. 2021;8. Available from: https://www.embase.com/search/results?subaction=viewrecord&id=L635340582&from=export U2 - L635340582
23. Cui Y, Zhang W, Ma Y, Zhan Z, Chen Y. Stability of blood eosinophils in acute exacerbation of chronic obstructive pulmonary disease and its relationship to clinical outcomes: a prospective cohort study. Respir Res [Internet]. 2021;22(1). Available from: https://www.embase.com/search/results?subaction=viewrecord&id=L2014298065&from=export U2 - L2014298065
24. Da Cruz DM, Ohara DG, De Castro SS, Jamami M. Hospitalization, deaths, expenses respiratory diseases and its relationship with climate change in the municipality of San Carlos - SP, Brazil. Internações Hosp óbitos, custos com doenças Respir e sua relação com alterações climáticas no município São Carlos - SP, Bras [Internet]. 2016;49(3):248–57. Available from: https://www.embase.com/search/results?subaction=viewrecord&id=L612434549&from=export U2 - L612434549
25. Deniz S, Şengül A, Aydemir Y, J ÇE, MH Ö. Clinical factors and comorbidities affecting the cost of hospital-treated COPD. Int J Chron Obstruct Pulmon Dis [Internet]. 2016;11:3023–30. Available from: https://pubmed.ncbi.nlm.nih.gov/27980399/
26. Dong F, Huang K, Ren X, Qumu S, Niu H, Wang Y, et al. Factors associated with inpatient length of stay among hospitalised patients with chronic obstructive pulmonary disease, China, 2016-2017: A retrospective study. BMJ Open [Internet]. 2021;11(2). Available from: https://www.embase.com/search/results?subaction=viewrecord&id=L634109182&from=export U2 - L634109182
27. Duangbubpha S, Hanucharurnkul S, Pookboonmee R, Orathai P, Kiatboonsri C. Chronic Care Model Implementation and Outcomes among Patients with COPD in Care Teams with and without Advanced Practice Nurses. Pacific Rim Int J Nurs Res [Internet]. 2013;17(2):102–16. Available from: https://search.ebscohost.com/login.aspx?direct=true&db=cin20&AN=104284666&authtype=shib&site=ehost-live
28. Dugee O, Munaa E, Sakhiya A, Mahal A. Mongolia's Public Spending On Noncommunicable Diseases Is Similar To The Spending Of Higher-Income Countries. Health Aff [Internet]. 2017;36(5):918–25. Available from: https://search.ebscohost.com/login.aspx?direct=true&db=cin20&AN=122825718&authtype=shib&site=ehost-live
29. Dzakwan NSAB, Hariadha E. Profile assessment for hospital readmission among male patients of acute

exacerbation chronic obstructive pulmonary disease at selected hospital in Malaysia. Asian J Pharm Clin Res [Internet]. 2017;10:21–4. Available from: https://www.embase.com/search/results?subaction=viewrecord&id=L617507142&from=export U2 - L617507142
30. E WJ, Jiazina T, Hou TT, A MGL. Clinical characteristics of coal worker's peneumoconiosis complicated with acute exacerbation of chronic obstractive pulno-mary disease. Zhonghua Lao Dong Wei Sheng Zhi Ye Bing Za Zhi [Internet]. 2021;39(9):661–4. Available from: https://www.embase.com/search/results?subaction=viewrecord&id=L636346397&from=export U2 - L636346397
31. Eniko B, Katalin B, Zsolt-Levente B, Georgeta DL, Marius DC, Cornel C, et al. Cost-effectiveness of home non-invasive ventilation in COPD group GOLD D patients. BALNEO PRM Res J. 2021;12(4):327–32.
32. Estrada J, Restrepo AM, Herrera R, Arrieta J, Serna JA, Segura Á. The economic outcome of pharmacotherapeutic follow-up in patients with chronic obstructive pulmonary disease at a health care institution, Medellín-Colombia, 2012-2014. Result económicos del Seguim Farmacoter en pacientes con Enferm Pulm Obstr crónica, en una Inst salud, Medellín-Colombia, 2012-2014 [Internet]. 2015;22:S176–9. Available from: https://www.embase.com/search/results?subaction=viewrecord&id=L608048325&from=export U2 - L608048325
33. Etchie TO, Etchie AT, Adewuyi GO, Pillarisetti A, Sivanesan S, Krishnamurthi K, et al. The gains in life expectancy by ambient PM2.5 pollution reductions in localities in Nigeria. Environ Pollut [Internet]. 2018;236:146–57. Available from: https://www.embase.com/search/results?subaction=viewrecord&id=L620557226&from=export U2 - L620557226
34. Fan L, Zhao Q, Liu Y, Zhou L, Duan J. Semiquantitative cough strength score and associated outcomes in noninvasive positive pressure ventilation patients with acute exacerbation of chronic obstructive pulmonary disease. Respir Med [Internet]. 2014;108(12):1801–7. Available from: https://www.embase.com/search/results?subaction=viewrecord&id=L603783625&from=export U2 - L603783625
35. Farias CC, Resqueti V, Dias FA, Borghi-Silva A, Arena R, Fregonezi GA. Costs and benefits of pulmonary rehabilitation in chronic obstructive pulmonary disease: a randomized controlled trial. Brazilian J Phys Ther [Internet]. 2014;18(2):165 ‐ 173. Available from: https://www.cochranelibrary.com/central/doi/10.1002/central/CN-00992117/full
36. Fernández-Plata R, Martínez-Briseño D, Figueroa CG-S, Cano-Jiménez D, Ramírez-Venegas A, Sansores-Martínez R, et al. Methods for estimating health costs of COPD: Baseline results. Métodos para la estimación costos en salud la EPOC Result basales [Internet]. 2016;75(1):4–11. Available from: https://www.embase.com/search/results?subaction=viewrecord&id=L2007504669&from=export U2 - L2007504669
37. Foo J, Landis SH, Maskell J, Oh Y-M, Van Der Molen T, Han MK, et al. Continuing to confront COPD international patient survey: economic impact of COPD in 12 countries. PLoS One. 2016;11(4):e0152618.
38. Fouladi Fard R, Naddafi K, Yunesian M, Nabizadeh Nodehi R, Dehghani MH, Hassanvand MS. The assessment of health impacts and external costs of natural gas-fired power plant of Qom. Env Sci Pollut Res Int [Internet]. 2016;23(20):20922–36. Available from: https://www.embase.com/search/results?subaction=viewrecord&id=L616196115&from=export U2 - L616196115
39. Fu X, Li L, Lei Y, Wu S, Yan D, Luo X, et al. The economic loss of health effect damages from PM2.5 pollution in the Central Plains Urban Agglomeration. Env Sci Pollut Res Int [Internet]. 2020;27(20):25434–49. Available from: https://www.embase.com/search/results?subaction=viewrecord&id=L631664539&from=export U2 - L631664539
40. Gaygol'Nik T V, Demko I V, Bochanova EN, Bikulova T V, Kraposhina AY, Solov'Eva IA, et al. Drug supplying management for patients with chronic obstructive pulmonary disease at Krasnoyarsk kray. Pulmonologiya [Internet]. 2016;26(2):208–14. Available from: https://www.embase.com/search/results?subaction=viewrecord&id=L614676993&from=export U2 - L614676993
41. Gaygol'Nik T V, Demko I V, Bochanova EN, Gordeeva N V, Kraposhina AY, Solov'Eva IA. Pharmacoeconomic analysis of therapy of acute exacerbation of chronic obstructive pulmonary disease in a large Krasnoyarsk hospital. Pulmonologiya [Internet]. 2015;25(3):320–6. Available from: https://www.embase.com/search/results?subaction=viewrecord&id=L613697164&from=export U2 - L613697164
42. Ghobadi M, MR F, Nakhaee N, Jafari-Sirizi M, Barouni M. Estimation of the Cost of Smoking-Attributable Diseases (Five Selected Diseases): A Case in Kerman City, Iran, 2014. Addict Heal [Internet]. 2017;9(4):190–8. Available from: https://pubmed.ncbi.nlm.nih.gov/30574281/
43. Ghoshal A, Ravindran G, Gangwal P, Rajadhyaksha G, Cho S-H, Muttalif A, et al. The burden of segregated respiratory diseases in India and the quality of care in these patients: Results from the Asia-Pacific Burden of

Respiratory Diseases study. Lung India [Internet]. 2016;33(6):611–9. Available from: https://www.embase.com/search/results?subaction=viewrecord&id=L613175183&from=export U2 - L613175183
44. Gong C, Yang Y, Chen M, Xie Z. Effect of procalcitonin on the prognosis of patients with COPD. Biomed reports [Internet]. 2020;12(6):313-318. Available from: https://www.cochranelibrary.com/central/doi/10.1002/central/CN-02163941/full
45. Gong S, Hu H, Zhao K, Yang T. Cost-effectiveness of dual bronchodilator indacaterol/glycopyrronium for copd treatment in China. Int J COPD [Internet]. 2021;16:433–41. Available from: https://www.embase.com/search/results?subaction=viewrecord&id=L2006158618&from=export U2 - L2006158618
46. Gutiérrez Ávila SA, Domínguez Borgua A, Valenzuela Plata A. Efficacy of clinical criteria and risk factors in the diagnosis of chronic obstructive pulmonary disease. Efic los criterios clínicos y factores riesgo en el diagnóstico Enferm Pulm Obstr crónica [Internet]. 2014;30(3):247–56. Available from: https://www.embase.com/search/results?subaction=viewrecord&id=L373369772&from=export U2 - L373369772
47. Hadei M, Shahsavani A, Krzyzanowski M, Querol X, Stafoggia M, Nazari SSH, et al. Burden of mortality attributed to PM2.5 exposure in cities of Iran; contribution of short-term pollution peaks. Atmos Environ [Internet]. 2020;224. Available from: https://www.embase.com/search/results?subaction=viewrecord&id=L2005058939&from=export U2 - L2005058939
48. Hoang Anh PT, Thu leT., Ross H, Quynh Anh N, Linh BN, Minh NT. Direct and indirect costs of smoking in Vietnam. Tob Control [Internet]. 2016;25(1):96–100. Available from: https://www.embase.com/search/results?subaction=viewrecord&id=L615305541&from=export U2 - L615305541
49. Hong Y, Liu Q, Bai L, Jiang L, Han X, Huang S, et al. Head-To-Head Comparison of Treatment Failure and Costs among COPD Patients Who Used Noninvasive Ventilation in the Ward versus in the ICU: A Propensity-Matched Cohort Study. Can Respir J [Internet]. 2020;2020. Available from: https://www.embase.com/search/results?subaction=viewrecord&id=L2010675649&from=export U2 - L2010675649
50. Huang J, Li G, Xu G, Qian X, Zhao Y, Pan X, et al. The burden of ozone pollution on years of life lost from chronic obstructive pulmonary disease in a city of Yangtze River Delta, China. Environ Pollut [Internet]. 2018;242:1266–73. Available from: https://www.embase.com/search/results?subaction=viewrecord&id=L2001028344&from=export U2 - L2001028344
51. Huang J, Li J, Yin P, Wang L, Pan X, Zhou M, et al. Ambient nitrogen dioxide and years of life lost from chronic obstructive pulmonary disease in the elderly: A multicity study in China. Chemosphere [Internet]. 2021;275. Available from: https://www.embase.com/search/results?subaction=viewrecord&id=L2011160546&from=export U2 - L2011160546
52. Ignatova GL, Antonov VN, Rodionova O V. Economic efficacy of vaccination of patients with chronic obstructive pulmonary disease and coronary heart disease. Pulmonologiya [Internet]. 2015;25(3):312–9. Available from: https://www.embase.com/search/results?subaction=viewrecord&id=L613697153&from=export U2 - L613697153
53. Ignatova GL, Antonov VN, Rodionova O V, Grebneva I V, Bel'Sner MS. Evaluation of quality of life and prognostic scales in patients with chronic obstructive pulmonary disease. Pulmonologiya [Internet]. 2016;26(4):473–80. Available from: https://www.embase.com/search/results?subaction=viewrecord&id=L614678618&from=export U2 - L614678618
54. Ignatova GL, Zakharova IA, Antonov Г.Л. VNZИ, Захарова ИА, Антонов ВН. Clinical and Economic Efficiency of Vaccination in a Pneumococcal 13-Valent Conjugate Vaccine in Patients with Chronic Bronchitis at a Young Age FT Клинико-экономическая эффективность вакцинации конъюгированной пневмококковой 13-в. Epidemiol i vaktsinoprofilaktika S1 Эпидемиология и вакцинопрофилактика. 2017;16(2):17–22.
55. Inchai J, Keeratiruangrong K, Liwsrisakun C, Deesomchok A, Bumroongkit C, Theerakittikul T, et al. Influence of comorbidities on hospital mortality and healthcare utilization in hospitalized chronic obstructive pulmonary disease patients. J Med Assoc Thail [Internet]. 2020;103(7):673–9. Available from: https://www.embase.com/search/results?subaction=viewrecord&id=L2007091297&from=export U2 - L2007091297
56. Iqbal MS, Al-Saikhan FI, Ahmed NJ, Iqbal MZ. Pharmacoeconomic Analysis of Acute Exacerbation of Chronic Obstructive Pulmonary Disease. J Pharm Res Int. 2020;32(4):59–66.
57. Iqbal MS, Al-Saikhan FI, Iqbal MZ. Out-of-pocket Healthcare Costs of COPD Exacerbation Episodes: A Hidden Cost and Growing Strain on Family Budgets. J Pharm Res Int. 2020;32(5):42–8.

58. Isaacs AA, Manga N, Le Grange C, Titus V, Sayed R, Hellenberg DA. A snapshot of noncommunicable disease profiles and their prescription costs at ten primary healthcare facilities in the in the western half of the Cape Town Metropole. South African Fam Pract [Internet]. 2014;56(1):43–9. Available from: https://search.ebscohost.com/login.aspx?direct=true&db=cin20&AN=95474011&authtype=shib&site=ehost-live
59. Ishmurzin Геннадий Петрович GPZИ. Medico-economic performance of treatment of patients with chronic obstructive pulmonary disease and bronchial asthma in case of emergency admission FT Медико-экономические показатели амбулаторного и госпитального лечения пациентов. Kazan Med J S1 Казанский медицинский журнал. 2016;97(6):950–8.
60. Jha P, Wu DC, Sheel V, Gupta P, Essue BM, Luong L. Impact of cigarette tax increase on health and financing outcomes in four Indian states. Gates Open Res [Internet]. 2020;4. Available from: https://www.embase.com/search/results?subaction=viewrecord&id=L633402438&from=export U2 - L633402438
61. Jinfeng Z, Qingli D, Juan C, Yuling L, Zhemei H. Analysis of clinical effects of early enteral nutrition standardized treatment process management on patients with acute exacerbation of chronic obstructive pulmonary disease on invasive mechanical ventilation. Zhonghua Wei Zhong Bing Ji Jiu Yi Xue [Internet]. 2020;32(1):67–71. Available from: https://www.embase.com/search/results?subaction=viewrecord&id=L2005811421&from=export U2 - L2005811421
62. Kallaru H, Nagasubramanian VR, Balakrishnan HP, Gopal K, Palani T. Impact of severity of the disease on cost of illness and quality of life of patients with chronic obstructive pulmonary disease. J Young Pharm [Internet]. 2015;7(2):106–12. Available from: https://www.embase.com/search/results?subaction=viewrecord&id=L602768936&from=export U2 - L602768936
63. Kamusheva M, Dimitrova M, JF van B, MJ P, T van der M, JW K, et al. Clinical characteristics, treatment patterns, and socio-economic burden of COPD in Bulgaria. J Med Econ [Internet]. 2017;20(5):503–9. Available from: https://pubmed.ncbi.nlm.nih.gov/28058859/
64. Kara E, Ozdilek HG, Kara EE, Balci F, Mestav B. Ambient Air Quality and General Health Outcomes in Nigde (Turkey) between 2011 and 2017. Iran J Public Health. 2021;50(10):1963-1972 WE-Science Citation Index Expande.
65. Kontsevaya A V, Mukaneyeva DK, Balanova YA, Khudyakov MB, Drapkina OM. Economic burden of respiratory diseases and chronic obstructive pulmonary disease in Russian Federation, 2016. Экономический ущерб от болезней органов дыхания и хронической обструктивной болезни легких в Российской Федерации в 2016 году [Internet]. 2019;29(2):159–66. Available from: https://www.embase.com/search/results?subaction=viewrecord&id=L2002231805&from=export U2 - L2002231805
66. Koul PA, Nowshehri AA, Khan UH, Jan RA, Shah SU. Cost of severe chronic obstructive pulmonary disease exacerbations in a high burden region in North India. Ann Glob Heal [Internet]. 2019;85(1). Available from: https://www.embase.com/search/results?subaction=viewrecord&id=L2001786936&from=export U2 - L2001786936
67. Kristina SA, Endarti D, Wiedyaningsih C, Fahamsya A, Faizah N. Health Care Cost of Noncommunicable Diseases Related to Smoking in Indonesia, 2015. Asia-Pacific J Public Heal [Internet]. 2018;30(1):29–35. Available from: https://search.ebscohost.com/login.aspx?direct=true&db=cin20&AN=128060503&authtype=shib&site=ehost-live
68. Lakiang T, NS N, Ramaswamy A, Singhal U. Economic impact of chronic obstructive pulmonary disease: A cross-sectional study at teaching hospital in South India. J Fam Med Prim care [Internet]. 2018;7(5):1002–6. Available from: https://pubmed.ncbi.nlm.nih.gov/30598947/
69. Lan B, Zhang DW, Yuan Y, Wang F, Liu QX. Observation on the clinical efficacy of ambroxol hydrochloride in the treatment of AECOPD combined with pulmonary infection. Prog Mod Biomed [Internet]. 2013;13:5549-5553. Available from: https://www.cochranelibrary.com/central/doi/10.1002/central/CN-02184042/full
70. Li CL, Hu YJ, Zhang F, Chen JM, Ma Z, Ye XN, et al. Multi-pollutant emissions from the burning of major agricultural residues in China and the related health-economic effects. Atmos Chem Phys. 2017;17(8):4957–88.
71. Li F, Sun Z, Li H, Yang T, Shi Z. Factors associated with hospitalisation costs in patients with chronic obstructive pulmonary disease. Int J Tuberc lung Dis Off J Int Union against Tuberc Lung Dis [Internet]. 2018;22(4):458–63. Available from: https://pubmed.ncbi.nlm.nih.gov/29562996/
72. Li J, Wang Y, Yin P, Huang J, Wu Z, Cao R, et al. The burden of sulfur dioxide pollution on years of life lost from chronic obstructive pulmonary disease: A nationwide analysis in China. Environ Res [Internet]. 2021;194. Available from: https://www.embase.com/search/results?subaction=viewrecord&id=L2010111536&from=export U2 - L2010111536

73. Li J, Zhu Y, Kelly JT, Jang CJ, Wang S, Hanna A, et al. Health benefit assessment of PM2.5 reduction in Pearl River Delta region of China using a model-monitor data fusion approach. J Environ Manag [Internet]. 2019;233:489–98. Available from: https://www.embase.com/search/results?subaction=viewrecord&id=L2001442397&from=export U2 - L2001442397
74. Li M, Wang F, Chen R, Liang Z, Zhou Y, Yang Y, et al. Factors contributing to hospitalization costs for patients with COPD in China: A retrospective analysis of medical record data. Int J COPD [Internet]. 2018;13:3349–57. Available from: https://www.embase.com/search/results?subaction=viewrecord&id=L2001618419&from=export U2 - L2001618419
75. Li P, Gong Y, Zeng G, Ruan L, Li G. A new mode of community continuing care service for COPD patients in China: Participation of respiratory nurse specialists. Int J Clin Exp Med [Internet]. 2015;8(9):15878–88. Available from: https://www.embase.com/search/results?subaction=viewrecord&id=L606756446&from=export U2 - L606756446
76. Liang L, Li C, Shen Y, Rong H, Jing H, Tong Z. Long-term trends in hospitalization and outcomes in adult patients with exacerbation of chronic obstructive pulmonary disease in Beijing, China, from 2008 to 2017. Int J COPD [Internet]. 2020;15:1155–64. Available from: https://www.embase.com/search/results?subaction=viewrecord&id=L2004427961&from=export U2 - L2004427961
77. Liang L, Shang Y, Xie W, Shi J, Tong Z, Jalali MS. Trends in hospitalization expenditures for acute exacerbations of copd in Beijing from 2009 to 2017. Int J COPD [Internet]. 2020;15:1165–75. Available from: https://www.embase.com/search/results?subaction=viewrecord&id=L2004427960&from=export U2 - L2004427960
78. Liao Q, Jin W, Tao Y, Qu J, Li Y, Niu Y. Health and economic loss assessment of PM2.5 pollution during 2015–2017 in Gansu Province, China. Int J Environ Res Public Heal [Internet]. 2020;17(9). Available from: https://www.embase.com/search/results?subaction=viewrecord&id=L2004311785&from=export U2 - L2004311785
79. Lin SH, He YP, Lian JJ, Chu CK. Procalcitonin kinetics to guide sequential invasive-noninvasive mechanical ventilation weaning in patients with acute exacerbation of chronic obstructive pulmonary disease and respiratory failure: procalcitonin's adjunct role. Libyan J Med [Internet]. 2021;16(1):1961382. Available from: https://www.cochranelibrary.com/central/doi/10.1002/central/CN-02304486/full
80. Linthicum MT, Thornton Snider J, Vaithianathan R, Wu Y, LaVallee C, Lakdawalla DN, et al. Economic burden of disease-associated malnutrition in China. Asia Pac J Public Heal [Internet]. 2015;27(4):407–17. Available from: https://www.embase.com/search/results?subaction=viewrecord&id=L611927394&from=export U2 - L611927394
81. Liu HL, Wang N, Chen W, Liu WY, Wang SP, Lei JB, et al. Hospitalization Trends in Adult Patients with COPD and Other Respiratory Diseases in Northeast China from 2005 to 2015. Biomed Res Int. 2018;2018.
82. Liu M, Liu J, Geng Z, Bai S. Evaluation of outcomes of medication therapy management (Mtm) services for patients with chronic obstructive pulmonary disease (copd). Pakistan J Med Sci [Internet]. 2021;37(7):1832-1836. Available from: https://www.cochranelibrary.com/central/doi/10.1002/central/CN-02336277/full
83. Lu X, Lin C, Li Y, Yao T, Fung JCH, Lau AKH. Assessment of health burden caused by particulate matter in southern China using high-resolution satellite observation. Environ Int [Internet]. 2017;98:160–70. Available from: https://www.embase.com/search/results?subaction=viewrecord&id=L613380159&from=export U2 - L613380159
84. Luo L, Li J, Lian S, Zeng X, Sun L, Li C, et al. Using machine learning approaches to predict high-cost chronic obstructive pulmonary disease patients in China. Health Informatics J [Internet]. 2020;26(3):1577–98. Available from: https://search.ebscohost.com/login.aspx?direct=true&db=cin20&AN=144846437&authtype=shib&site=ehost-live
85. Maji KJ, Arora M, Dikshit AK. Burden of disease attributed to ambient PM2.5 and PM10 exposure in 190 cities in China. Env Sci Pollut Res Int [Internet]. 2017;24(12):11559–72. Available from: https://www.embase.com/search/results?subaction=viewrecord&id=L616925778&from=export U2 - L616925778
86. Maji KJ, Dikshit AK, Deshpande A. Assessment of City Level Human Health Impact and Corresponding Monetary Cost Burden due to Air Pollution in India Taking Agra as a Model City. AEROSOL AIR Qual Res. 2017;17(3):831–42.
87. Maji KJ, Dikshit AK, Deshpande A. Disability-adjusted life years and economic cost assessment of the health effects related to PM2.5 and PM10 pollution in Mumbai and Delhi, in India from 1991 to 2015. Env Sci Pollut Res Int [Internet]. 2017;24(5):4709–30. Available from: https://www.embase.com/search/results?subaction=viewrecord&id=L617664075&from=export U2 - L617664075
88. Maji KJ, Ye W-F, Arora M, Shiva Nagendra SM. PM2.5-related health and economic loss assessment for 338

Chinese cities. Environ Int [Internet]. 2018;121:392–403. Available from: https://www.embase.com/search/results?subaction=viewrecord&id=L2001116521&from=export U2 - L2001116521
89. Mao X, Liang C, Niu H, Dong F, Huang K, Chen Y, et al. Outcomes associated with comorbid diabetes among patients with COPD exacerbation: findings from the ACURE registry. Respir Res [Internet]. 2021;22(1). Available from: https://www.embase.com/search/results?subaction=viewrecord&id=L2010122871&from=export U2 - L2010122871
90. Meghji J, Gregorius S, Madan J, Chitimbe F, Thomson R, Rylance J, et al. The long term effect of pulmonary tuberculosis on income and employment in a low income, urban setting. Thorax. 2021;76(4):387–95.
91. MJ U, Alam N, TP K, Sarma H, MA C, DS A, et al. Consequences of hypertension and chronic obstructive pulmonary disease, healthcare-seeking behaviors of patients, and responses of the health system: a population-based cross-sectional study in Bangladesh. BMC Public Health [Internet]. 2014;14:547. Available from: https://pubmed.ncbi.nlm.nih.gov/24888580/
92. Mueller W, Vardoulakis S, Steinle S, Loh M, Johnston HJ, Precha N, et al. A health impact assessment of long-term exposure to particulate air pollution in Thailand. Environ Res Lett. 2021;16(5).
93. Nagi M, Riewpaiboon A, Thavorncharoensap M. Cost of premature mortality attributable to smoking in the middle east and north africa. Coût la mortalité prématurée imputable au tabagisme dans la région moyenorient afrique du Nord [Internet]. 2021;27(10):974–83. Available from: https://www.embase.com/search/results?subaction=viewrecord&id=L2014642114&from=export U2 - L2014642114
94. Nair M, Bherwani H, Mirza S, Anjum S, Kumar R. Valuing burden of premature mortality attributable to air pollution in major million-plus non-attainment cities of India. Sci Rep [Internet]. 2021;11(1):22771. Available from: https://www.embase.com/search/results?subaction=viewrecord&id=L636843489&from=export U2 - L636843489
95. Nedogoda S V, Yu FM, Salasyuk AS, Barykina IN, Smirnova VO. Pharmacoeconomic analysis of tiotropium bromide and olodaterol fixed combination as maintenance therapy for patients with COPD in the Russian Federation. Фармакоэкономический анализ применения фиксированной комбинации тиотропия бромид+олодатерол в качестве поддерживающей терапии у пациентов с ХОБЛ в Российской Федерации [Internet]. 2020;13(2):101–11. Available from: https://www.embase.com/search/results?subaction=viewrecord&id=L2007647533&from=export U2 - L2007647533
96. Nevárez-Sida A, Castro-Bucio AJ, García-Contreras F, Cisneros-González N. Costos Medicos Directos en Pacientes Con Enfermedad Pulmonar Obstructiva Crónica en Mexico Direct Medical Cost in Pacients with Chronic Obstructive Pulmonary Disease in Mexico. Value Heal Reg Issues [Internet]. 2017;14:9–14. Available from: https://www.embase.com/search/results?subaction=viewrecord&id=L615635398&from=export U2 - L615635398
97. Ngo CQ, Bui TT, Vu G V, Chu HT, Phan PT, Pham HN, et al. Direct hospitalization cost of patients with acute exacerbation of chronic obstructive pulmonary disease in Vietnam. Int J Environ Res Public Heal [Internet]. 2019;16(1). Available from: https://www.embase.com/search/results?subaction=viewrecord&id=L625710175&from=export U2 - L625710175
98. Orlova EA, Umerova AR, Dorfman IP, Orlov MA, Abdullaev MA. Estimation of socio-economic burden of chronic obstructive pulmonary disease for a 5-year period: A regional aspect. ОЦЕНКА СОЦИАЛЬНО-ЭКОНОМИЧЕСКОГО БРЕМЕНИ ХРОНИЧЕСКОЙ ОБСТРУКТИВНОЙ БОЛЕЗНИ ЛЕГКИХ ЗА 5-ЛЕТНИЙ ПЕРИОД – РЕГИОНАЛЬНЫЙ АСПЕКТ [Internet]. 2021;9(2):130–8. Available from: https://www.embase.com/search/results?subaction=viewrecord&id=L2013986448&from=export U2 - L2013986448
99. Örnek T, Atalay F, Erboy F, Altinsoy B, Tanriverdi H, Uygur F, et al. Is pneumoconiosis a factor of severity in acute exacerbation of chronic obstructive pulmonary disease? Clin Ter [Internet]. 2014;164(6):e473–7. Available from: https://www.embase.com/search/results?subaction=viewrecord&id=L372623151&from=export U2 - L372623151
100. Ortaköylü MG, Altın S, Bahadır A, Ürer HN, Koşar F, Coskun A. Activity-based costing management and hospital cost in patients with chronic obstructive pulmonary disease. Kron Obs akciğer Hast olan Hast Faal tabanlı maliyet yöntemi ile Hastan maliyeti [Internet]. 2016;13(2):116–26. Available from: https://www.embase.com/search/results?subaction=viewrecord&id=L610078318&from=export U2 - L610078318
101. Ozdemir T, Kilic H, Demirci NY, Ozdilekcan C, Bektemur G, Turkkani MH, et al. Five-Year Trends in Direct Costs of Chronic Obstructive Pulmonary Disease in Turkey: COPDTURKEY-3. Turk Toraks Derg / Turkish Thorac J [Internet]. 2021;22(5):393–8. Available from: https://search.ebscohost.com/login.aspx?direct=true&db=cin20&AN=153483935&authtype=shib&site=ehost-live

102. Pan Z, Dickens AP, Chi C, Kong X, Enocson A, G Cooper B, et al. Accuracy and cost-effectiveness of different screening strategies for identifying undiagnosed COPD among primary care patients (=40 years) in China: A cross-sectional screening test accuracy study: Findings from the Breathe Well group. BMJ Open [Internet]. 2021;11(9). Available from: https://www.embase.com/search/results?subaction=viewrecord&id=L636080638&from=export U2 - L636080638
103. Patanavanich R, Aekplakorn W, Suriyawongpaisal P. Trend analysis of smoking-attributable hospitalizations in Thailand, 2007-2014. Tob Induc Dis [Internet]. 2018;16:1–10. Available from: https://search.ebscohost.com/login.aspx?direct=true&db=cin20&AN=135261923&authtype=shib&site=ehost-live
104. Patel K, Lalwani T, Shah K. Economic Burden in Direct Cost of Chronic Obstructive Pulmonary Disease at a Tertiary Care Teaching Hospital: A Prospective Observational Cohort Study. Indian J Pharm Pract. 2014 Sep 9;7:61–8.
105. Pavlovic R, Stojkov S, Binakaj Z. Costs of treatment of severe copd exacerbation in Serbia. Troškovi lečenja teškog pogoršanja hobp-A u Srb [Internet]. 2020;21(1):51–8. Available from: https://www.embase.com/search/results?subaction=viewrecord&id=L2004807917&from=export U2 - L2004807917
106. Peker K. Cost analysis according to diagnosis in tertiary care patients in a university hospital. Bir Üniversite Hastan Üçüncü Basamak Yoğun Bakım Hast Tanıya Göre Maliyet Anal [Internet]. 2019;27(4):265–71. Available from: https://www.embase.com/search/results?subaction=viewrecord&id=L2004764262&from=export U2 - L2004764262
107. Peng X, Yu J, Zhang W, Li Y, Wu M, Xu T, et al. Drug usage and costs of inpatients with chronic obstructive pulmonary disease in Karamay Central Hospital in 2014. Chin J Evid-Based Med [Internet]. 2017;17(8):888–91. Available from: https://www.embase.com/search/results?subaction=viewrecord&id=L623184481&from=export U2 - L623184481
108. Pichon-Riviere A, Alcaraz A, Palacios A, Rodríguez B, Reynales-Shigematsu LM, Pinto M, et al. The health and economic burden of smoking in 12 Latin American countries and the potential effect of increasing tobacco taxes: an economic modelling study. Lancet Glob Heal [Internet]. 2020;8(10):e1282–94. Available from: https://www.embase.com/search/results?subaction=viewrecord&id=L2007882946&from=export U2 - L2007882946
109. Pichon-Riviere A, Bardach A, Augustovski F, Alcaraz A, Reynales-Shigematsu LM, Teixeira Pinto M, et al. Impacto económico del tabaquismo en los sistemas de salud de América Latina: un estudio en siete países y su extrapolación a nivel regional. Rev Panam Salud Pública [Internet]. 2016;40(4):213–21. Available from: https://search.ebscohost.com/login.aspx?direct=true&db=cin20&AN=120443551&authtype=shib&site=ehost-live
110. Piroozi B, Amerzadeh M, Safari H, Mohamadi-Bolbanabad A, Afkhamzadeh A, Zarezadeh Y, et al. The burden of preventable hospitalizations before and after implementation of the health transformation plan in a hospital in west of Iran. Prim Heal Care Res Dev [Internet]. 2019;20:e87. Available from: https://www.embase.com/search/results?subaction=viewrecord&id=L632620895&from=export U2 - L632620895
111. Pothirat C, Liwsrisakun C, Bumroongkit C, Deesomchok A, Theerakittikul T, Limsukon A. Comparative study on health care utilization and hospital outcomes of severe acute exacerbation of chronic obstructive pulmonary disease managed by pulmonologists vs internists. [Internet]. Vol. 10, International journal of chronic obstructive pulmonary disease. 2015. p. 759–66. Available from: https://pubmed.ncbi.nlm.nih.gov/25926727/
112. Qian W, Huang G-Z. Neutrophil CD64 as a Marker of Bacterial Infection in Acute Exacerbations of Chronic Obstructive Pulmonary Disease. Immunol Invest [Internet]. 2016;45(6):490–503. Available from: https://www.embase.com/search/results?subaction=viewrecord&id=L610510393&from=export U2 - L610510393
113. Qu S, You X, Liu T, Wang L, Yin Z, Liu Y, et al. Cost-effectiveness analysis of COPD screening programs in primary care for high-risk patients in China. npj Prim Care Respir Med [Internet]. 2021;31(1). Available from: https://www.embase.com/search/results?subaction=viewrecord&id=L2011574729&from=export U2 - L2011574729
114. Rahul S, Abhinand CR, Nikithareddy B, Jayachandra K, Lakshmi P, Doddayya H, et al. Pharmacoeconomic evaluation of acute exacerbations of chronic obstructive pulmonary disease at a tertiary care teaching hospital in North Karnataka, India. Asian J Pharm Clin Res [Internet]. 2018;11(5):463–6. Available from: https://www.embase.com/search/results?subaction=viewrecord&id=L621965155&from=export U2 - L621965155
115. Ramanath K V, Thomas AJ, Joy L, Thomas V. Assessment of drug utilization pattern in asthma, chronic obstructive pulmonary disease and pneumonia: In a rural tertiary care teaching hospital. Res J Pharm, Biol

Chem Sci [Internet]. 2016;7(5):748–56. Available from: https://www.embase.com/search/results?subaction=viewrecord&id=L612220196&from=export U2 - L612220196
116. Reechaipichitkul W. Precipitating causes and outcomes of chronic obstructive pulmonary disease exacerbation at a tertiary care center in northeast Thailand. Asian Biomed [Internet]. 2014;8(2):229–36. Available from: https://www.embase.com/search/results?subaction=viewrecord&id=L373801065&from=export U2 - L373801065
117. Rezaei S, Karami Matin B, Hajizadeh M, Bazyar M, Akbari Sari A. Economic Burden of Smoking in Iran: A Prevalence-Based Annual Cost Approach. Asian Pac J Cancer Prev [Internet]. 2017;18(10):2867–73. Available from: https://www.embase.com/search/results?subaction=viewrecord&id=L630217795&from=export U2 - L630217795
118. Saiphoklang N, Kanitsap A, Ruchiwit P, Pirompanich P, Sricharoenchai T, Cooper C. Patient characteristics and outcomes of a home mechanical ventilation program in a developing country. Lung India [Internet]. 2019;36(3):207–11. Available from: https://www.embase.com/search/results?subaction=viewrecord&id=L627459627&from=export U2 - L627459627
119. Salameh P, Khayat G, Waked M. Diagnostic score for COPD: Validation of the DS-COPD in clinical settings. Clin Epidemiol Glob Heal [Internet]. 2013;1(3):107–14. Available from: https://www.embase.com/search/results?subaction=viewrecord&id=L370364061&from=export U2 - L370364061
120. Salem A, Zhong H, Ramos M, Lamotte M, Hu H. Potential clinical and economic impact of optimised maintenance therapy on discharged patients with COPD after hospitalisation for an exacerbation in China. BMJ Open [Internet]. 2021;11(4). Available from: https://www.embase.com/search/results?subaction=viewrecord&id=L634890110&from=export U2 - L634890110
121. Samarnkongsak T, Thavorncharoensap M, Kawamatawong T, Pattanaprateep O, Chanjaruporn F, Sunantiwat M. Treatment of COPD in one university hospital setting in Thailand: The real-life prescribing patterns and treatment expenditures. Pharma Sci Asia [Internet]. 2019;46(3):175–83. Available from: https://www.embase.com/search/results?subaction=viewrecord&id=L629017286&from=export U2 - L629017286
122. Sapunova ID, Kontsevaya A V, Myrzamatova AO, Mukaneeva DK, Khudyakov MB, Ipatov P V, et al. Economic damage from smoking associated with four groups of chronic non-communicable diseases in the Russian Federation in 2016. Экономический ущерб от курения, ассоциированный с четырьмя группами хронических неинфекционных заболеваний в Российской Федерации в 2016 году [Internet]. 2019;18(6):6–12. Available from: https://www.embase.com/search/results?subaction=viewrecord&id=L2005010347&from=export U2 - L2005010347
123. Satici C, Arpinar Yigitbas B, Seker B, Demirkol MA, Kosar AF. Does Adherence to Domiciliary NIMV Decrease the Subsequent Hospitalizations Rates and Cost for Patients Diagnosed with COPD? COPD J Chronic Obstr Pulm Dis [Internet]. 2018;15(3):303–9. Available from: https://www.embase.com/search/results?subaction=viewrecord&id=L623910211&from=export U2 - L623910211
124. Shang D, Dang X-M, Yang L, Han J-F, Sun Z-M, Feng S-F, et al. Treatment of respiratory muscle fatigue in patients with AECOPD by non-invasive positive pressure ventilation. J Xi'An Jiaotong Univ Med Sci [Internet]. 2014;35(6):824–7 and 847. Available from: https://www.embase.com/search/results?subaction=viewrecord&id=L600557292&from=export U2 - L600557292
125. Shi M, Wang J, Zhang L, Yan Y, Miao Y-D, Zhang X. Effects of Integrated Case Payment on Medical Expenditure and Readmission of Inpatients with Chronic Obstructive Pulmonary Disease: A Nonrandomized, Comparative Study in Xi County, China. Curr Med Sci [Internet]. 2018;38(3):558–66. Available from: https://www.embase.com/search/results?subaction=viewrecord&id=L624804384&from=export U2 - L624804384
126. Sichali JM, Khan JAK, Gama EM, Banda HT, Namakhoma I, Bongololo G, et al. Direct costs of illness of patients with chronic cough in rural Malawi-Experiences from Dowa and Ntchisi districts. PLoS One [Internet]. 2019;14(12). Available from: https://www.embase.com/search/results?subaction=viewrecord&id=L2004475209&from=export U2 - L2004475209
127. SN S, Sweeney S, Seeley J, Biraro S, Mutungi G, Munderi P, et al. The health system burden of chronic disease care: an estimation of provider costs of selected chronic diseases in Uganda. Trop Med Int Health [Internet]. 2015;20(6):781–90. Available from: https://pubmed.ncbi.nlm.nih.gov/25707376/
128. Stâmbu I, IP S. [Estimation of direct medical costs of chronic obstructive pulmonary disease over 12 months]. [Internet]. Vol. 62, Pneumologia (Bucharest, Romania). Romania; 2013. p. 86–92. Available from:

https://pubmed.ncbi.nlm.nih.gov/23894789/
129. Subramanian S, Gakunga R, Kibachio J, Gathecha G, Edwards P, Ogola E, et al. Cost and affordability of non-communicable disease screening, diagnosis and treatment in Kenya: Patient payments in the private and public sectors. PLoS One [Internet]. 2018;13(1). Available from: https://www.embase.com/search/results?subaction=viewrecord&id=L620083110&from=export U2 - L620083110
130. Sutanto YS, Makhabah DN, Aphridasari J, Doewes M, Suradi null, Ambrosino N. Videogame assisted exercise training in patients with chronic obstructive pulmonary disease: a preliminary study. Pulmonology [Internet]. 2019;25(5):275-282. Available from: https://www.cochranelibrary.com/central/doi/10.1002/central/CN-01942457/full
131. Szpak R, Strapasson GC, Böger B, Rattmann YD, Gomes EC. Legal demands of the tiotropium bromide for treatment of chronic obstructive pulmonary disease and their financial impact for the State of Paraná, Brazil. Einstein (Sao Paulo) [Internet]. 2020;18:eGS4442. Available from: https://www.embase.com/search/results?subaction=viewrecord&id=L629491472&from=export U2 - L629491472
132. Tabyshova A, Estebesova B, Beishenbekova A, Sooronbaev T, Brakema EA, Chavannes NH, et al. Clinical characteristics, treatment patterns and economic burden of COPD in Kyrgyzstan: A FRESH AIR study. Int J COPD [Internet]. 2021;16:2833–43. Available from: https://www.embase.com/search/results?subaction=viewrecord&id=L2014188970&from=export U2 - L2014188970
133. Tachkov K, Dimitrova M, Mitov K, Savova A, Kamusheva M, Dimitrov J, et al. Micro and macro analysis on the burden of COPD hospitalizations on the Bulgarian healthcare system. Biotechnol Biotechnol Equip. 2019;33(1):1–11.
134. Tachkov K, Kamusheva M, Pencheva V, Mitov K. Evaluation of the economic and social burden of chronic obstructive pulmonary disease (COPD). Biotechnol Biotechnol Equip. 2017;31(4):855–61.
135. Tachkov K, Mitov K, Savova A, Kamusheva M. A one-way, static markov model estimating the social and economic burden of chronic obstructive pulmonary disease in bulgaria for patients who smoke or don’t. Biomed Res [Internet]. 2018;29(16):3261–6. Available from: https://www.embase.com/search/results?subaction=viewrecord&id=L624250015&from=export U2 - L624250015
136. Tan J, Hao L, Cheng Y, Xu T, Song Y. [Clinical pathway management of acute exacerbations of chronic obstructive pulmonary disease based on state machine]. [Internet]. Vol. 34, Nan fang yi ke da xue xue bao = Journal of Southern Medical University. China; 2014. p. 568–70. Available from: https://pubmed.ncbi.nlm.nih.gov/24752111/
137. Tanriverdi H. Direct cost analyse of COPD patients in Erzincan state hospital. Erzincan Devl Hastan Koah’lı Hast doğrudan maliyet Anal [Internet]. 2013;15(2):15–8. Available from: https://www.embase.com/search/results?subaction=viewrecord&id=L372444894&from=export U2 - L372444894
138. Tarin-Carrasco P, Morales-Suarez-Varela M, Im U, Brandt J, Palacios-Pena L, Jimenez-Guerrero P. Isolating the climate change impacts on air-pollution-related-pathologies over central and southern Europe - a modelling approach on cases and costs. Atmos Chem Phys. 2019;19(14):9385–98.
139. Thanaviratananich S, SH C, AG G, ARBA M, HC L, Pothirat C, et al. Burden of respiratory disease in Thailand: Results from the APBORD observational study. Medicine (Baltimore) [Internet]. 2016;95(28):e4090. Available from: https://pubmed.ncbi.nlm.nih.gov/27428193/
140. Titova ON, Volchkov VA, Kuzubova NA, Kozyrev Ольга Николаевна AGZT, ВОЛЧКОВ ВА, КУЗУБОВА НА, et al. THE ORGANIZATIONAL ASPECTS OF ADMINISTRATION OF LONG-DURATION OXYGEN THERAPY IN CONDITIONS OF ST. PETERSBURG FT Организационные аспекты назначения длительной кислородотерапии в условиях Санкт-Петербурга. Zdr Ross Fed S1 Здравоохранение Российской Федерации. 2014;58(2):35–8.
141. Torabipour A, Hakim A, K AA, Dolatshah M, Yusofzadeh M. Cost Analysis of Hospitalized Patients with Chronic Obstructive Pulmonary Disease: A State-Level Cross-Sectional Study. Tanaffos [Internet]. 2016;15(2):75–82. Available from: https://pubmed.ncbi.nlm.nih.gov/27904538/
142. Turan O, JC E, Deniz S, Baysak A, PA T, Mirici A. Adherence to Current COPD Guidelines in Turkey. Expert Opin Pharmacother [Internet]. 2016;17(2):153–8. Available from: https://pubmed.ncbi.nlm.nih.gov/26629809/
143. Ture DA, Demirci H, Sengoren Dikis O. The relationship between health literacy and disease specific costs in subjects with chronic obstructive pulmonary disease (COPD). Aging Male [Internet]. 2021;23(5):396–402. Available from: https://www.embase.com/search/results?subaction=viewrecord&id=L624106933&from=export U2 - L624106933
144. Tuvdendorj A, Dechinkhorloo O, Dorjsuren B, Buskens E, Feenstra T. The costs of inappropriate referral pathways in inpatient care for three major noncommunicable diseases in Mongolia: a national registry-based analysis. BMC Heal Serv Res [Internet]. 2021;21(1):1280. Available from: https://www.embase.com/search/results?subaction=viewrecord&id=L636816378&from=export U2 -

L636816378
145. ur Rehman A, Hassali MAA, Muhammad SA, Shakeel S, Chin OS, Ali IABH, et al. Economic Burden of Chronic Obstructive Pulmonary Disease Patients in Malaysia: A Longitudinal Study. PharmacoEcon Open [Internet]. 2021;5(1):35–44. Available from: https://www.embase.com/search/results?subaction=viewrecord&id=L2010539214&from=export U2 - L2010539214
146. Varmaghani M, Ghobadi M, Sharifi F, Roshanfekr P, Sheidaei A, Mansouri M, et al. The economic burden of smoking-attribution and years of life lost due to chronic diseases in Mashhad, 2015-2016. Int J Prev Med [Internet]. 2021;12(1). Available from: https://www.embase.com/search/results?subaction=viewrecord&id=L634387296&from=export U2 - L634387296
147. Varol Y, Varol U, Başer Z, Usta L, Balcı G, Özacar R. The Cost of COPD Exacerbations Managed in Hospital. Turk Toraks Derg / Turkish Thorac J [Internet]. 2013;14(1):19–23. Available from: https://search.ebscohost.com/login.aspx?direct=true&db=cin20&AN=104256139&authtype=shib&site=ehost-live
148. Vidyakina EE, Malchikova E.Э. SVZB, Мальчикова СВ. Pharmacoeconomic analysis of the inpatient care for COPD FT Фармакоэкономический анализ терапии пациентов с хронической обструктивной болезнью легких в стационарных условиях. Klin Farmakol i Ter S1 Клиническая фармакология и терапия. 2016;25(5):86–8.
149. Villarreal-Ríos E, YJ J-H, ER V-D, Tapia-Mendoza F, Galicia-Rodríguez L, Martínez-González L. [Cost of medical attention in patients with Chronic Obstructive Pulmonary Disease]. [Internet]. Vol. 56, Revista medica del Instituto Mexicano del Seguro Social. Mexico; 2018. p. 371–8. Available from: https://pubmed.ncbi.nlm.nih.gov/30521740/
150. Vo TQ, Phung TCN, Vu TQ, Tran TN, Vo TTT, Phan VHA, et al. Cost trend analysis of chronic obstructive pulmonary disease among Vietnamese patients: Findings from two provincial facilities 2015–2017. J Clin Diagn Res [Internet]. 2018;12(6):LC92–8. Available from: https://www.embase.com/search/results?subaction=viewrecord&id=L623032462&from=export U2 - L623032462
151. Vu TQ, Vo TQ. Economic outcomes among chronic obstructive pulmonary disease Vietnamese patients: Approach considerations from a social perspective. J Pak Med Assoc [Internet]. 2019;69(6):S64–74. Available from: https://www.embase.com/search/results?subaction=viewrecord&id=L629006213&from=export U2 - L629006213
152. Wang J, Li P, Wen J. Impacts of the zero mark-up drug policy on hospitalization expenses of COPD inpatients in Sichuan province, western China: an interrupted time series analysis. BMC Heal Serv Res [Internet]. 2020;20(1):519. Available from: https://www.embase.com/search/results?subaction=viewrecord&id=L632017839&from=export U2 - L632017839
153. Wang L, Gu W, Zhang X, Fu S, Zhang D, Guan X, et al. How the cost-effectiveness results change in the China health policy environment: an economic evaluation of glycopyrrolate/formoterol for the treatment of COPD. J Med Econ [Internet]. 2022;25(1):356–66. Available from: https://pubmed.ncbi.nlm.nih.gov/35184671/
154. Wang S-M, Zhang F-Y, Du C-L, Wang X-B, Li F, Hang J-Q, et al. Epidemiology and issues of NIV-treated AECOPD patients with hypercapnic respiratory failure in Shanghai: A multicentre retrospective survey. Clin Respir J [Internet]. 2021;15(5):550–7. Available from: https://www.embase.com/search/results?subaction=viewrecord&id=L2010597129&from=export U2 - L2010597129
155. Wang Z, Zhou Y, Zhang Y, Huang X, Duan X, Chen D, et al. Association of change in air quality with hospital admission for acute exacerbation of chronic obstructive pulmonary disease in Guangdong, China: A province-wide ecological study. Ecotoxicol Environ Saf [Internet]. 2021;208. Available from: https://www.embase.com/search/results?subaction=viewrecord&id=L2008568239&from=export U2 - L2008568239
156. Wei G-R, Shi X-M. Evaluation the Extent of Health Damage Caused by PM2.5 Particulate in Xi'an City. Huan Jing Ke Xue [Internet]. 2018;39(7):3014–21. Available from: https://www.embase.com/search/results?subaction=viewrecord&id=L626410554&from=export U2 - L626410554
157. Widyastuti K, Makhabah DN, Setijadi AR, Sutanto YS, Suradi null, Ambrosino N. Benefits and costs of home pedometer assisted physical activity in patients with COPD. A preliminary randomized controlled trial. Pulmonology [Internet]. 2018;24(4):211-218. Available from: https://www.cochranelibrary.com/central/doi/10.1002/central/CN-01913430/full
158. Wu L, Lan N, Yang X. Effects of empirical glucocorticoid use on severe acute exacerbation of copd during hospitalization. Int J COPD [Internet]. 2021;16:2419–31. Available from: https://www.embase.com/search/results?subaction=viewrecord&id=L2013569765&from=export U2 -

L2013569765
159. Xu XM, Zhang W, Zhu C, Li JR, Yuan WP, Lv JL. Regional sources and the economic cost assessment of PM2.5 in Ji'nan, eastern China. Atmos Pollut Res. 2021;12(2):386–94.
160. Xu Z, Zhu L, Xu X, Zhu L, Feng H, Zhu A, et al. Preliminary study of the effect of outpatient oxygen therapy on patients with chronic respiratory insufficiency receiving home oxygen therapy. Ann Palliat Med [Internet]. 2022;11(2):513–20. Available from: https://www.embase.com/search/results?subaction=viewrecord&id=L637435969&from=export U2 - L637435969
161. Xuan J, Wang L, Yin H, Xuan D, Zhou Y, Hu S. The cost-effectiveness of OM-85 in managing respiratory tract infections in China. J Med Econ. 2015;18(3):167–72.
162. Yagudra RI, Skrypnyk Р.И. ARZЯ, Скрипник АР. COMPARATIVE ECONOMIC MODELING OF RESTRICTIONS TOBACCO SMOKING IN THE RUSSIAN FEDERATION (from 2009 to 2016) FT СРАВНИТЕЛЬНОЕ ЭКОНОМИЧЕСКОЕ МОДЕЛИРОВАНИЕ ПОСЛЕДСТВИЙ ТАБАКОКУРЕНИЯ В РОССИЙСКОЙ ФЕДЕРАЦИИ (С 2009 ПО 2016 гг.). Farmakoekon Teor i Prakt S1 Фармакоэкономика теория и практика. 2018;6(2):31–7.
163. Yang C, Long J, Shi Y, Zhou Z, Wang J, Zhao M-H, et al. Healthcare resource utilisation for chronic kidney disease and other major non-communicable chronic diseases in China: A cross-sectional study. BMJ Open [Internet]. 2022;12(1). Available from: https://www.embase.com/search/results?subaction=viewrecord&id=L636989511&from=export U2 - L636989511
164. Ye X, Li Z, Zhou X, Ruan X, Lin T, Zhou J, et al. The Impact of a Health Forecasting Service on the Visits and Costs in Outpatient and Emergency Departments for COPD Patients - Shanghai Municipality, China, October 2019-April 2020. China CDC Wkly [Internet]. 2021;3(23):495–9. Available from: https://pubmed.ncbi.nlm.nih.gov/34594921/
165. Yesildag K, Aydemir O. The Burden of Chronic Obstructive Lung Disease Disease on the Health System. EURASIAN J Emerg Med. 2021;20(4):269–76.
166. Yilmaz C, Özkan S, Erer OF. Risk assessment and rate of readmission within 30 days of discharge after hospitalization for acute exacerbation of chronic obstructive pulmonary disease. Alevlenme nedeniyle Hastan yatan Kron Obs akciğer Hast taburcu olduktan sonraki 30 gün içerisindehastaneye yeniden yatış risk faktörleri [Internet]. 2021;69(3):328–37. Available from: https://www.embase.com/search/results?subaction=viewrecord&id=L2014093298&from=export U2 - L2014093298
167. Yin H, Brauer M, Zhang JJ, Cai W, Navrud S, Burnett R, et al. Population ageing and deaths attributable to ambient PM2·5 pollution: a global analysis of economic cost. Lancet Planet Heal [Internet]. 2021;5(6):e356–67. Available from: https://www.embase.com/search/results?subaction=viewrecord&id=L2012929470&from=export U2 - L2012929470
168. Yin H, Xu L, Cai Y. Monetary valuation of PM10-related health risks in Beijing China: The necessity for PM10 pollution indemnity. Int J Environ Res Public Heal [Internet]. 2015;12(8):9967–87. Available from: https://www.embase.com/search/results?subaction=viewrecord&id=L605682001&from=export U2 - L605682001
169. Yin H, Pizzol M, Xu L. External costs of PM2.5 pollution in Beijing, China: Uncertainty analysis of multiple health impacts and costs. Environ Pollut [Internet]. 2017;226:356–69. Available from: https://www.sciencedirect.com/science/article/pii/S0269749116308600
170. You L, Niu H, Huang K, Dong F, Yang T, Wang C. Clinical features and outcomes of acute exacerbation in chronic obstructive pulmonary disease patients with pulmonary heart disease: A multicenter observational study. Int J COPD [Internet]. 2021;16:2901–10. Available from: https://www.embase.com/search/results?subaction=viewrecord&id=L2014303435&from=export U2 - L2014303435
171. YP Q, Zhao K, Li X, LW S, WD G, XR Q, et al. [Health economic evaluation of a 23 value pneumococcal polysaccharide vaccination pilot programme among elderly chronic obstructive pulmonary disease patients in China]. Zhonghua Yu Fang Yi Xue Za Zhi [Internet]. 2016;50(12):1074–8. Available from: https://pubmed.ncbi.nlm.nih.gov/28057111/
172. Yu X, Yang S, Li H, Xie Y, Li J, Zhang P. Preliminary Study to Evaluate Three Different Treatments on Stable Chronic Obstructive Pulmonary Disease Patients Based on Markov Model. Evidence-based Complement Altern Med [Internet]. 2019;1–16. Available from: https://search.ebscohost.com/login.aspx?direct=true&db=cin20&AN=135926203&authtype=shib&site=ehost-live
173. Zeng Q, Wang H, Wang K, Zhou H, Wang T, Wen F. Eosinophilic phenotype was associated with better early clinical remission in elderly patients but not middle-aged patients with acute exacerbations of COPD. Int J Clin Pr [Internet]. 2021;75(9). Available from: https://www.embase.com/search/results?subaction=viewrecord&id=L2012346548&from=export U2 -

L2012346548
174. Zeng XB, Deng JH, Gui JP, He D, Li N. Clinical research on procalcitonin guidance on the use and termination of antibiotic treatment in acute exacerbation of chronic obstructive pulmonary disease. Prog Mod Biomed [Internet]. 2017;17:3310-3312. Available from: https://www.cochranelibrary.com/central/doi/10.1002/central/CN-02190316/full
175. Zhang A, Wang L, Long L, Yan J, Liu C, Zhu S, et al. Effectiveness and Economic Evaluation of Hospital-Outreach Pulmonary Rehabilitation for Patients with Chronic Obstructive Pulmonary Disease. Int J Chron Obstruct Pulmon Dis [Internet]. 2020;15:1071-1083. Available from: https://www.cochranelibrary.com/central/doi/10.1002/central/CN-02289172/full
176. Zhang H, Niu Y, Yao Y, Chen R, Zhou X, Kan H. The impact of ambient air pollution on daily hospital visits for various respiratory diseases and the relevant medical expenditures in Shanghai, China. Int J Environ Res Public Heal [Internet]. 2018;15(3). Available from: https://www.embase.com/search/results?subaction=viewrecord&id=L620901137&from=export U2 - L620901137
177. Zhang H, Song Y, Zhang X, Hu J, Yuan S, Ma J. Extent and cost of inappropriate use of tumour markers in patients with pulmonary disease: a multicentre retrospective study in Shanghai, China. BMJ Open [Internet]. 2018;8(2):e019051. Available from: https://pubmed.ncbi.nlm.nih.gov/29490961/
178. Zhang J, Miller A, Li Y, Lan Q, Zhang N, Chai Y, et al. Comparison of Multiple Chronic Obstructive Pulmonary Disease (COPD) Indices in Chinese COPD Patients. Tuberc Respir Dis [Internet]. 2018;81(2):116–22. Available from: https://www.embase.com/search/results?subaction=viewrecord&id=L621866843&from=export U2 - L621866843
179. Zhang J, Yao W, You X, Liu T, Liu Y. Comparative analysis of medical expenditure with nebulized budesonide versus systemic corticosteroids in hospitalized patients with acute exacerbations of chronic obstructive pulmonary disease in China. Int J COPD [Internet]. 2019;14:1195–207. Available from: https://www.embase.com/search/results?subaction=viewrecord&id=L2002310894&from=export U2 - L2002310894
180. Zhang P, Zhou X. Pricing air pollution: evidence from short-term exposure to air pollution on hospitalization of acute bronchitis and chronic obstructive pulmonary disease in southwestern China. Int Heal [Internet]. 2021; Available from: https://www.embase.com/search/results?subaction=viewrecord&id=L636834653&from=export U2 - L636834653
181. Zhang W, Yu J, Liu J, Li Y, Wang F, Xu T, et al. Constituation and costs of inpatients with respiratory disease in Karamay Central Hospital in 2014: A cross-sectional study. Chin J Evid-Based Med [Internet]. 2017;17(5):507–10. Available from: https://www.embase.com/search/results?subaction=viewrecord&id=L623184703&from=export U2 - L623184703
182. Zheng J-P, Zhang J, Ma L-J, Chen P, Huang M, Ou X-M, et al. Clinical outcomes of using nebulized budesonide as the initial treatment for acute exacerbations of chronic obstructive pulmonary disease: A post-hoc analysis. Int J COPD [Internet]. 2019;14:2725–31. Available from: https://www.embase.com/search/results?subaction=viewrecord&id=L2003264756&from=export U2 - L2003264756
183. Zhou Y, Long E, Xu Q, Wang L, Jiang X, Hu M. Cost-Effectiveness Analysis of Triple Combination Preparations in the Treatment of Moderate-to-Severe Chronic Obstructive Pulmonary Disease. Front Public Heal [Internet]. 2021;9:713258. Available from: https://www.embase.com/search/results?subaction=viewrecord&id=L635908100&from=export U2 - L635908100
184. Zhu B, Pang R, Chevallier J, Wei Y-M, Vo D-T. Including Intangible Costs into the Cost-of-Illness Approach: A Method Refinement Illustrated Based on the PM2.5 Economic Burden in China. Eur J Heal Econ. 2019;20(4):501–11.
185. Zhu YF, Gao Z, Zhu M, Cai S, Zhou H. Clinical effect of cephalosporin combined with herba houttuyniae on senile chronic bronchitis. Prog Mod Biomed [Internet]. 2015;15:6919-6921. Available from: https://www.cochranelibrary.com/central/doi/10.1002/central/CN-02190280/full
186. Zyryanov SK, D'yakov IN. Pharmacoeconomic assessment of dual bronchodilation therapy in patients with COPD. Pulmonologiya [Internet]. 2018;28(1):61–8. Available from: https://www.embase.com/search/results?subaction=viewrecord&id=L622111468&from=export U2 - L622111468
187. 谭锦风, 刘瑜, 谢玉芬, 谢冬英. 家庭雾化吸入治疗慢性阻塞性肺疾病急性发作的疗效观察. Nurs Integr Tradit Chinese West Med [Internet]. 2018;4(6):22–4. Available from: https://search.ebscohost.com/login.aspx?direct=true&db=cin20&AN=134682505&authtype=shib&site=ehost-live
188. 赖静. 基于 DRGs 为导向的 ICU 慢性阻塞性肺疾病患者护理服务成本研究. Nurs Integr Tradit Chinese West Med [Internet]. 2019;5(3):1–5. Available from: https://search.ebscohost.com/login.aspx?direct=true&db=cin20&AN=136896914&authtype=shib&site=ehost-

live
