# Supplementary material for: Economic burden of chronic obstructive pulmonary disease and post-tuberculosis sequelae in low- and middle-income countries: a database compiled from a systematic review and meta-analysis
Source: BMJ Public Health. 2024 Jul 30;2(1):e000441. doi: 10.1136/bmjph-2023-000441 (PMC11816951; doi:10.1136/bmjph-2023-000441)
Supplement: online supplemental file 2 [file bmjph-2-1-s002.pdf]

The economic burden of chronic obstructive pulmonary disease and post-tuberculosis sequelae in low- and middle-income countries: a database compiled from a systematic review and meta-analysis

Yuling Lin[1], Alexandra Walker[2,3], Marguerite Batta[2,3], Sierra Ottilie-Kovelman[2,4], Anna Duchenko[2,3], Curdin Brugger[2,3], Olivia Keiser[1], Robert S. Wallis[5], Klaus Reither[2,3], Fabrizio Tediosi[2,3] , Marina Antillon[2,3*]

1 Institute of Global Health, University of Geneva, 1205 Geneva, Switzerland
2 Swiss Tropical and Public Health Institute (Swiss TPH), Kreuzstrasse 2, 4123 Allschwil, Switzerland
3 University of Basel, Peterspl. 1, 4001 Basel, Switzerland
4 Yale School of Public Health, New Haven, Connecticut 06510, United States
5 The Aurum Institute, Johannesburg, South Africa
*Corresponding author

Supplemental Tables of Cost Data

Contents

2-I Table of cost data 1
2.1 COPD, without intervention, by income group 1
2.2 COPD, without intervention, by continent 8
2.3 AECOPD, without intervention, by income group 14
2.4 AECOPD, without intervention, by continent 22
2.5 CB, by income level and continent 30
2.6 Other disease, without intervention, by income level and continent 31
2.7 With intervention, by disease and country 31
2.8 Costs stratified by sex, without intervention 39
2.9 Costs stratified by age, without intervention 39
2.10 Cost stratified by disease severity, without intervention 42
2.11 Costs stratified by comorbidity, without intervention 43

2-I Table of cost data

Costs are expressed in terms of 2021 USD values. These costs are means and medians are expressed by placing the value in parentheses [mean (median)].

2.1 COPD, without intervention, by income group

Table S2.1 Cost of COPD by income group among non-intervention studies

Income group	Country	Reference ID	Subgroup	Unit	Length of hospitalization stay (day)	Medication costs	Emergency visit costs	Hospitalization costs	Direct medical costs	Direct non-medical costs	Indirect costs
Lower middle income	India	Lakiang T, 2018		Per hospitalization		21		205	530	132	
		Rahul S, 2018		Per patient	9	45			54	11	
		Kallaru H, 2015	Overall: GOLD II, III, IV	Per patient per hospitalization		12		99	221	29	22
			GOLD II	Per patient per hospitalization		2		0	26	3	0
			GOLD III	Per patient per hospitalization	9	12		105	232	38	6
			GOLD IV	Per patient per hospitalization	11.8	40		345	559	70	126
		Patel K, 2014		Per patient per hospitalization	5.6	22			45	10	
	Iran	Sari AA, 2017		Per patient				1693			
	Kenya	Subramanian S, 2018	Public facilities	Per patient					419		
			Private facilities	Per patient					1720		
	Kyrgyzstan	Tabyshova A, 2021		Per year per patient	10.1	62		131	398		
	Mongolia	Tuvdendorj A, 2021		Per bed-day over 2016-2018	8.7			26			
	Vietnam	Anh PTH, 2016		Per hospitalization and per outpatient visit				780			
		Vu TQ, 2019	Inpatients	Per patient		99		59	189	56	147

Income group	Country	Reference ID	Subgroup	Unit	Length of hospitalization stay (day)	Medication costs	Emergency visit costs	Hospitalization costs	Direct medical costs	Direct non-medical costs	Indirect costs
			Total Outpatient	Per patient		39			43	15	34
			Outpatient (GOLD III)	Per patient		31			35	14	35
			Outpatient (GOLD IV)	Per patient		42			47	15	33
		Vo TQ, 2018	Bien Hoa city, Inpatient	Per patient	7.9 (7)				206		
			Ho Chi Minh city, Inpatient	Per patient	11.1 (10)	167			303		
Upper middle income	Brazil	Foo J, 2016	Brazil	Per year per patient		78	29	88			1923
	Bulgaria	Kamusheva M, 2017	The whole study population	Per year per patient	12	924		727	1697		6062
			GOLD I	Per year per patient							767
			GOLD II	Per year per patient				(288)			924
			GOLD III	Per year per patient				(482)			
			GOLD IV	Per year per patient				(602)			1800
	China	Chen X, 2016		Per year per patient				1656 (0)	2401 (1116)	108 (0)	419 (0)
			Per hospitailization	Per hospitalization				914 (0)			
		Dong F, 2021	The whole study population	Per patient per hospitalization	(10)			(2366)			
			length of stay ≥8 days	Per patient per hospitalization				(1576)			
			length of stay 9-14 days	Per patient per hospitalization				(2477)			
			length of stay >14 days	Per patient per hospitalization				(4668)			
		Liu H, 2018	2005	Per hospitalization	12			1198			
			2005, age 18-35	Per hospitalization	11.4			1133			
			2005, age 36-60	Per hospitalization	12.2			1209			

Income group	Country	Reference ID	Subgroup	Unit	Length of hospitalization stay (day)	Medication costs	Emergency visit costs	Hospitalization costs	Direct medical costs	Direct non-medical costs	Indirect costs
			2005, age >61	Per hospitalization	12.6			1255			
			2006	Per hospitalization	11.7			1067			
			2006, age 18-35	Per hospitalization	10.1			1042			
			2006, age 36-60	Per hospitalization	12.4			1022			
			2006, age >61	Per hospitalization	12.7			1235			
			2007	Per hospitalization	12.5			1190			
			2007, age 18-35	Per hospitalization	12.2			1229			
			2007, age 36-60	Per hospitalization	12.7			1125			
			2007, age >61	Per hospitalization	12.6			1299			
			2008	Per hospitalization	11.8			1196			
			2008, age 18-35	Per hospitalization	10.6			1366			
			2008, age 36-60	Per hospitalization	12.3			1026			
			2008, age >61	Per hospitalization	12.3			1373			
			2009	Per hospitalization	11.5			1376			
			2009, age 18-35	Per hospitalization	10.2			1435			
			2009, age 36-60	Per hospitalization	12.2			1255			
			2009, age >61	Per hospitalization	12.1			1607			
			2010	Per hospitalization	11.4			1450			
			2010, age 18-35	Per hospitalization	10.2			1533			
			2010, age 36-60	Per hospitalization	12.1			1322			
			2010, age >61	Per hospitalization	11.8			1658			
			2011	Per hospitalization	11.1			1337			

Income group	Country	Reference ID	Subgroup	Unit	Length of hospitalization stay (day)	Medication costs	Emergency visit costs	Hospitalization costs	Direct medical costs	Direct non-medical costs	Indirect costs
			2011, age 18-35	Per hospitalization	9.3			1422			
			2011, age 36-60	Per hospitalization	12			1202			
			2011, age >61	Per hospitalization	11.6			1565			
			2012	Per hospitalization	10.8			1473			
			2012 age 18-35	Per hospitalization	9.1			1630			
			2012, age 36-60	Per hospitalization	11.6			1328			
			2012, age >61	Per hospitalization	11.4			1603			
			2013	Per hospitalization	10.5			1571			
			2013, age 18-35	Per hospitalization	9.5			1767			
			2013, age 36-60	Per hospitalization	10.9			1767			
			2013, age >61	Per hospitalization	11.2			1852			
			2014	Per hospitalization	10.4			1699			
			2014, age 18-35	Per hospitalization	9.6			2065			
			2014, age 36-60	Per hospitalization	10.7			1442			
			2014, age >61	Per hospitalization	11.1			1778			
			2015	Per hospitalization	9.8			1706			
			2015, age 18-35	Per hospitalization	8.6			2126			
			2015, age 36-60	Per hospitalization	10.3			1441			
			2015, age >61	Per hospitalization	10.7			1716			
		Zhang H, 2018a		Per day			531				
		Yang C, 2022	With chronic kidney disease	Per patient	(12)			(2166)			
			Without chronic kidney disease	Per patient	(10)			(1805)			

Income group	Country	Reference ID	Subgroup	Unit	Length of hospitalization stay (day)	Medication costs	Emergency visit costs	Hospitalization costs	Direct medical costs	Direct non-medical costs	Indirect costs
		Hong Y, 2020	In the ward before matching	Per patient	(10.2)			(3521)			
			In the ICU before matching	Per patient	(10.2)			(5280)			
			In the ward in the propensity-matched cohort	Per patient	(10.9)			(3535)			
			In the ICU in the propensity-matched cohort	Per patient	(8.8)			(4387)			
		Zhang W, 2017		Per patient	15	1235		2581			
	Colombia	Estrada JI, 2015	The whole study population	Per month per patient					(112)		
			Patients with pharmacological risk	Per month per patient					(81)		
			Patients without any pharmacological risk	Per month per patient					(127)		
			Patients who had missing doses	Per month per patient					(133)		
			Patients who had incorrect inhalation technique	Per month per patient					(97)		
	Jordan	Altawalbeh SM, 2021	COPD	Per patient				2383			
	Malaysia	Rehman AU, 2020		Per year per patient			87	301			1716
	Mexico	Foo J, 2016	Mexico	Per year per patient		69	17	112			2407
		Ávila SAG, 2014		Per patient over 6 months		208					
		Fernández-Plata R, 2016	Mild COPD	Per year per patient		(913)					(41)
			Moderate COPD	Per year per patient		(1031)					(66)
			Severe COPD	Per year per patient		(1136)					(48)
			Very severe COPD	Per year per patient		(1274)					(48)

Income group	Country	Reference ID	Subgroup	Unit	Length of hospitalization stay (day)	Medication costs	Emergency visit costs	Hospitalization costs	Direct medical costs	Direct non-medical costs	Indirect costs
		Nevárez-Aids A, 2017	Moderate COPD	Per year per patient		329	155	3240	1335		
			Severe COPD	Per year per patient		845	148	5775	2694		
		Villarreal-Rios E, 2018		Per year per patient			38	141			
	Romania	Strambu I, 2013		Per year per patient		409		2042	2475		
	Russia	Foo J, 2016	Russia	Per year per patient		122	15	112			672
		Akramova EG, 2014	With comorbidities	Per year per patient		13					
			COPD and CVD	Per year per patient		33					
			CPD+ hypertension	Per year per patient		19					
		Gaygolnik, 2015	2009	Per prescription			15				
			2010	Per prescription			18				
			2011	Per prescription			18				
			2012	Per prescription			16				
			2013	Per prescription			18				
			2014	Per prescription			20				
	South Africa	Isaacs AA, 2014		Per prescription		9					
	Thailand	Samarnkondsak T, 2019	Group 1 (mild + moderate, FEV1>=50%)	Per year per patient	0.5	305 (305)			428 (428)		
			Group 2 (severe + very severe, FEV1<50%)	Per year per patient	2.7	594 (584)			732 (736)		
		Thanaviratananich S, 2016		Per year per patient		1281		782			
	Turkey	Ortaköylü MG, 2016		Per patient			8				
		Turan O, 2016	Total study population	Per patient per month		29					

Income group	Country	Reference ID	Subgroup	Unit	Length of hospitalization stay (day)	Medication costs	Emergency visit costs	Hospitalization costs	Direct medical costs	Direct non-medical costs	Indirect costs
			Adherent to spirometric classification	Per patient per month		27					
			Inadherent to spirometric classification	Per patient per month		31					
			Adherent to combined classification	Per patient per month		27					
			Inadherent to combined classification	Per patient per month		31					
		Satici C, 2018	The whole study population	Per patient over 6 months	5.7			472			
			Adherent to NIV	Per patient over 6 months	2.7			359			
			Non-adherent to NIV	Per patient over 6 months	8.7			565			
		Peker K, 2019		Per patient		(1116)					
		Ture DA, 2021	With inadequate health literacy	Per patient			(32)	(33)	(167)		
			With adequate health literacy	Per patient			(20)	(25)	(123)		
			GOLD I	Per patient			(16)	(23)	(103)		
			GOLD II	Per patient			(27)	(28)	(142)		
			GOLD III	Per patient			(25)	(20)	(100)		
			GOLD IV	Per patient			(41)	(51)	(191)		
		Yeşildağ K, 2021	Secondary hospitals	Per patient	(7)	(15)		(257)			
			Tertiary hospitals	Per patient	(8)	(55)		(359)			
		Ozdemir T, 2021		Per patient	6.5		16	435			

2.2 COPD, without intervention, by continent

Table S2.2 Cost of COPD by continent among non-intervention studies

Continent	Country	Reference ID	Subgroup	Unit	Length of hospitalization stay (day)	Medication costs	Emergency visit costs	Hospitalization costs	Direct medical costs	Direct non-medical costs	Indirect costs
Africa	Kenya	Subramanian S, 2018	Public facilities	Per patient					419		
			Private facilities	Per patient					1720		
	South Africa	Isaacs AA, 2014		Per prescription		9					
Asia	China	Chen X, 2016		Per year per patient				1656 (0)	2401 (1116)	108 (0)	419 (0)
			Per hospitailization	Per hospitalization				914 (0)			
		Dong F, 2021	The whole study population	Per patient per hospitalization	(10)			(2366)			
			length of stay ≥8 days	Per patient per hospitalization				(1576)			
			length of stay 9-14 days	Per patient per hospitalization				(2477)			
			length of stay >14 days	Per patient per hospitalization				(4668)			
		Liu H, 2018	2005	Per hospitalization	12			1198			
			2005, age 18-35	Per hospitalization	11.4			1133			
			2005, age 36-60	Per hospitalization	12.2			1209			
			2005, age >61	Per hospitalization	12.6			1255			
			2006	Per hospitalization	11.7			1067			
			2006, age 18-35	Per hospitalization	10.1			1042			
			2006, age 36-60	Per hospitalization	12.4			1022			
			2006, age >61	Per hospitalization	12.7			1235			
			2007	Per hospitalization	12.5			1190			

Continent	Country	Reference ID	Subgroup	Unit	Length of hospitalization stay (day)	Medication costs	Emergency visit costs	Hospitalization costs	Direct medical costs	Direct non-medical costs	Indirect costs
			2007, age 18-35	Per hospitalization	12.2			1229			
			2007, age 36-60	Per hospitalization	12.7			1125			
			2007, age >61	Per hospitalization	12.6			1299			
			2008	Per hospitalization	11.8			1196			
			2008, age 18-35	Per hospitalization	10.6			1366			
			2008, age 36-60	Per hospitalization	12.3			1026			
			2008, age >61	Per hospitalization	12.3			1373			
			2009	Per hospitalization	11.5			1376			
			2009, age 18-35	Per hospitalization	10.2			1435			
			2009, age 36-60	Per hospitalization	12.2			1255			
			2009, age >61	Per hospitalization	12.1			1607			
			2010	Per hospitalization	11.4			1450			
			2010, age 18-35	Per hospitalization	10.2			1533			
			2010, age 36-60	Per hospitalization	12.1			1322			
			2010, age >61	Per hospitalization	11.8			1658			
			2011	Per hospitalization	11.1			1337			
			2011, age 18-35	Per hospitalization	9.3			1422			
			2011, age 36-60	Per hospitalization	12			1202			
			2011, age >61	Per hospitalization	11.6			1565			
			2012	Per hospitalization	10.8			1473			
			2012 age 18-35	Per hospitalization	9.1			1630			
			2012, age 36-60	Per hospitalization	11.6			1328			

Continent	Country	Reference ID	Subgroup	Unit	Length of hospitalization stay (day)	Medication costs	Emergency visit costs	Hospitalization costs	Direct medical costs	Direct non-medical costs	Indirect costs
			2012, age >61	Per hospitalization	11.4			1603			
			2013	Per hospitalization	10.5			1571			
			2013, age 18-35	Per hospitalization	9.5			1767			
			2013, age 36-60	Per hospitalization	10.9			1767			
			2013, age >61	Per hospitalization	11.2			1852			
			2014	Per hospitalization	10.4			1699			
			2014, age 18-35	Per hospitalization	9.6			2065			
			2014, age 36-60	Per hospitalization	10.7			1442			
			2014, age >61	Per hospitalization	11.1			1778			
			2015	Per hospitalization	9.8			1706			
			2015, age 18-35	Per hospitalization	8.6			2126			
			2015, age 36-60	Per hospitalization	10.3			1441			
			2015, age >61	Per hospitalization	10.7			1716			
		Zhang H, 2018a		Per day			531				
		Yang C, 2022	With chronic kidney disease	Per patient	(12)			(2166)			
			Without chronic kidney disease	Per patient	(10)			(1805)			
		Hong Y, 2020	In the ward before matching	Per patient	(10.2)			(3521)			
			In the ICU before matching	Per patient	(10.2)			(5280)			
			In the ward in the propensity-matched cohort	Per patient	(10.9)			(3535)			
			In the ICU in the propensity-matched cohort	Per patient	(8.8)			(4387)			
		Zhang W, 2017		Per patient	15	1235		2581			

Continent	Country	Reference ID	Subgroup	Unit	Length of hospitalization stay (day)	Medication costs	Emergency visit costs	Hospitalization costs	Direct medical costs	Direct non-medical costs	Indirect costs
	India	Lakiang T, 2018		Per hospitalization		21		205	530	132	
		Rahul S, 2018		Per patient	9	45			54	11	
		Kallaru H, 2015	Overall: GOLD II, III, IV	Per patient per hospitalization		12		99	221	29	22
			GOLD II	Per patient per hospitalization		2		0	26	3	0
			GOLD III	Per patient per hospitalization	9	12		105	232	38	6
			GOLD IV	Per patient per hospitalization	11.8	40		345	559	70	126
		Patel K, 2014		Per patient per hospitalization	5.6	22			45	10	
	Iran	Sari AA, 2017		Per patient				1693			
	Jordan	Altawalbeh SM, 2021	COPD	Per patient				2383			
	Kyrgyzstan	Tabyshova A, 2021		Per year per patient	10.1	62		131	398		
	Malaysia	Rehman AU, 2020		Per year per patient			87	301			1716
	Mongolia	Tuvdendorj A, 2021		Per bed-day over 2016-2018	8.7			26			
	Thailand	Samarnkondsak T, 2019	Group 1 (mild + moderate, FEV1>=50%)	Per year per patient	0.5	305 (305)			428 (428)		
			Group 2 (severe + very severe, FEV1<50%)	Per year per patient	2.7	594 (584)			732 (736)		
		Thanaviratananich S, 2016		Per year per patient		1281		782			
	Turkey	Turan O, 2016	Total study population	Per patient per month		29					
			Adherent to spirometric classification	Per patient per month		27					

Continent	Country	Reference ID	Subgroup	Unit	Length of hospitalization stay (day)	Medication costs	Emergency visit costs	Hospitalization costs	Direct medical costs	Direct non-medical costs	Indirect costs
			Inadherent to spirometric classification	Per patient per month		31					
			Adherent to combined classification	Per patient per month		27					
			Inadherent to combined classification	Per patient per month		31					
		Peker K, 2019		Per patient		(1116)					
		Ture DA, 2021	With inadequate health literacy	Per patient			(32)	(33)	(167)		
			With adequate health literacy	Per patient			(20)	(25)	(123)		
			GOLD I	Per patient			(16)	(23)	(103)		
			GOLD II	Per patient			(27)	(28)	(142)		
			GOLD III	Per patient			(25)	(20)	(100)		
			GOLD IV	Per patient			(41)	(51)	(191)		
		Yeşildağ K, 2021	Secondary hospitals	Per patient	(7)	(15)		(257)			
			Tertiary hospitals	Per patient	(8)	(55)		(359)			
	Vietnam	Anh PTH, 2016		Per hospitalization and per outpatient visit				780			
		Vu TQ, 2019	Inpatients	Per patient		99		59	189	56	147
			Total Outpatient	Per patient		39			43	15	34
			Outpatient (GOLD III)	Per patient		31			35	14	35
			Outpatient (GOLD IV)	Per patient		42			47	15	33
		Vo TQ, 2018	Bien Hoa city, Inpatient	Per patient	7.9 (7)				206		
			Ho Chi Minh city, Inpatient	Per patient	11.1 (10)	167			303		
Eastern Europe	Bulgaria	Kamusheva M, 2017	The whole study population	Per year per patient	12	924		727	1697		6062
			GOLD I	Per year per patient							767

Continent	Country	Reference ID	Subgroup	Unit	Length of hospitalization stay (day)	Medication costs	Emergency visit costs	Hospitalization costs	Direct medical costs	Direct non-medical costs	Indirect costs
			GOLD II	Per year per patient				(288)			924
			GOLD III	Per year per patient				(482)			
			GOLD IV	Per year per patient				(602)			1800
	Romania	Strambu I, 2013		Per year per patient		409		2042	2475		
	Russia	Foo J, 2016	Russia	Per year per patient		122	15	112			672
		Akramova EG, 2014	With comorbidities	Per year per patient		13					
			COPD and CVD	Per year per patient		33					
			CPD+ hypertension	Per year per patient		19					
		Gaygolnik, 2015	2009	Per prescription			15				
			2010	Per prescription			18				
			2011	Per prescription			18				
			2012	Per prescription			16				
			2013	Per prescription			18				
			2014	Per prescription			20				
	Turkey	Ortaköylü MG, 2016		Per patient			8				
		Satici C, 2018	The whole study population	Per patient over 6 months	5.7			472			
			Adherent to NIV	Per patient over 6 months	2.7			359			
			Non-adherent to NIV	Per patient over 6 months	8.7			565			
		Ozdemir T, 2021		Per patient	6.5		16	435			
Latin America	Brazil	Foo J, 2016	Brazil	Per year per patient		78	29	88			1923
	Colombia	Estrada JI, 2015	The whole study population	Per month per patient					(112)		

Continent	Country	Reference ID	Subgroup	Unit	Length of hospitalization stay (day)	Medication costs	Emergency visit costs	Hospitalization costs	Direct medical costs	Direct non-medical costs	Indirect costs
			Patients with pharmacological risk	Per month per patient					(81)		
			Patients without any pharmacological risk	Per month per patient					(127)		
			Patients who had missing doses	Per month per patient					(133)		
			Patients who had incorrect inhalation technique	Per month per patient					(97)		
	Mexico	Foo J, 2016	Mexico	Per year per patient		69	17	112			2407
	Mexico	Ávila SAG, 2014		Per patient over 6 months		208					
	Mexico	Fernández-Plata R, 2016	Mild COPD	Per year per patient		(913)					(41)
	Mexico	Fernández-Plata R, 2016	Moderate COPD	Per year per patient		(1031)					(66)
	Mexico	Fernández-Plata R, 2016	Severe COPD	Per year per patient		(1136)					(48)
	Mexico	Fernández-Plata R, 2016	Very severe COPD	Per year per patient		(1274)					(48)
	Mexico	Nevárez-Aids A, 2017	Moderate COPD	Per year per patient		329	155	3240	1335		
	Mexico	Nevárez-Aids A, 2017	Severe COPD	Per year per patient		845	148	5775	2694		
	Mexico	Villarreal-Rios E, 2018		Per year per patient			38	141			

2.3 AECOPD, without intervention, by income group

Table S2.3 Cost of AECOPD by income group among non-intervention studies

Income group	Country	Reference ID	Subgroup	Unit	Length of hospitalization stay (day)	Medication costs	Emergency visit costs	Hospitalization costs	Direct medical costs	Direct non-medical costs	Indirect costs
	India	Koul PA, 2019		Per exacerbation	(8)	(115)		(625)			

Income group	Country	Reference ID	Subgroup	Unit	Length of hospitalization stay (day)	Medication costs	Emergency visit costs	Hospitalization costs	Direct medical costs	Direct non-medical costs	Indirect costs
Lower middle income		Altaf M, 2015	Salmeterol/fluticasone	Per patient over 6 months	4.8						55
			Formoterol/budesonide	Per patient over 6 months	6						60
			Formoterol/fluticasone	Per patient over 6 months	7.4						67
	Iran	Torabipour A, 2016	2011	Per year per patient	8.5 (5)	120		370	632		
			2012	Per year per patient	8.5 (5)	301		761	1296		
			2013	Per year per patient	8.5 (5)	540		985	1893		
			Length of stay ≤ 9 days	Per patient	8.5 (5)	176 (90)		303 (156)	631 (371)		
			Length of stay > 9 days	Per patient	8.5 (5)	759 (438)		1942 (1273)	3232 (2344)		
			Age <65	Per patient	8.5 (5)				1037 (380)		
			Age >65	Per patient	8.5 (5)				1354 (621)		
			Female	Per patient	8.5 (5)				1640 (496)		
			Male	Per patient	8.5 (5)				1097 (506)		
	Pakistan	Iqbal MS, 2020b	Severity III	Per exacerbation	4.2	49					
			Severity II	Per exacerbation	9.2	62					
			Severity level I	Per exacerbation	13	81					
	Vietnam	Ngo CQ, 2019	The whole study population	Per patient	8.9	499		854			
			GOLD I	Per patient	5.3	126		299			
			GOLD II	Per patient	8.4	378		658			
			GOLD III	Per patient	9.3	537		914			
			GOLD IV	Per patient	9.3	579		975			
Upper middle income	China	Li F, 2018	Total study population	Per patient	8.5	1045		2012			
			High-cost group	Per patient	10.2			2818			
			Low-cost group	Per patient	6.9			1175			
			Length of stay in hospital <8 days	Per patient		675		1522			

Income group	Country	Reference ID	Subgroup	Unit	Length of hospitalization stay (day)	Medication costs	Emergency visit costs	Hospitalization costs	Direct medical costs	Direct non-medical costs	Indirect costs
			Length of stay in hospital ≥8 days	Per patient		1378		2423			
		Li M, 2018		Per patient per hospitalization	9.4 (7)	1898 (1010)		4171 (2517)			
		Mao X, 2021	The whole study population	Per patient				(1576)			
			With diabetes	Per patient				(1778)			
			Without diabetes	Per patient				(1551)			
		Wang S, 2021	The whole study population	Per patient	15.3			3859			
			Peripheral blood eosinophils <2%	Per patient	15.7			4191			
			Peripheral blood eosinophils ≥2%	Per patient	15			3315			
		Zhang J, 2018	Before disease assessment test	Per patient	12.6 (13)		16				
			Before disease assessment test	Per patient	18.5 (15)		57				
		Cui Y, 2022	AECOPD with coronary artery disease	Per patient	10			1571			
			AECOPD without coronary artery disease	Per patient	9			1496			
		Fan L, 2014	NPPV success group	Per patient	15.9			9970			
			NPPV failure group	Per patient	17.5			15442			
		You L, 2021	Without pulmonary heart disease	Per patient	(9)	(600)		(1557)			
			With pulmonary heart disease	Per patient	(10)	(618)		(1619)			
		Zeng Q, 2021	Total study population, eosinophilic group	Per patient	10.5			(1684)			
			Total study population, non-eosinophilic group	Per patient	11.9			(2072)			
			Eosinophilic group, aged >65	Per patient	10.7			(1819)			
			Non-eosinophilic group, aged >65	Per patient	11.9			(2116)			
			Eosinophilic group, aged 45-65	Per patient	9.1			(1255)			

Income group	Country	Reference ID	Subgroup	Unit	Length of hospitalization stay (day)	Medication costs	Emergency visit costs	Hospitalization costs	Direct medical costs	Direct non-medical costs	Indirect costs
			Non-eosinophilic group, aged 45-65	Per patient	11.4			(1631)			
		Chen R, 2020	High adherence group (before index), inhale bronchodilator	Per year per patient				1577 (717)			
			High adherence group (before index), combined	Per year per patient				1460 (624)			
			High adherence group (before index), oral therapy	Per year per patient				4070 (1025)			
			High adherence group (after index), inhale bronchodilator users	Per year per patient				1691			
			High adherence group (after index), combined	Per year per patient				1514			
			High adherence group (after index), oral therapy	Per year per patient				5732			
			Low adherence group (before index), inhale bronchodilator	Per year per patient				1232 (172)			
			Low adherence group (before index), combined	Per year per patient				1078 (243)			
			Low adherence group (before index), oral therapy	Per year per patient				1076 (80)			
			Low adherence group (after index), inhale bronchodilator	Per year per patient				2034			
			Low adherence group (after index), combined	Per year per patient				2437			
			Low adherence group (after index), oral therapy	Per year per patient				2232			
		Cui Y, 2021a	Group LL	Per patient	(10)			3049			
			Group LH	Per patient	(12)			4896			
			Group HL	Per patient	(10)			2485			
			Group HH	Per patient	(11)			2343			

Income group	Country	Reference ID	Subgroup	Unit	Length of hospitalization stay (day)	Medication costs	Emergency visit costs	Hospitalization costs	Direct medical costs	Direct non-medical costs	Indirect costs
		Cui Y, 2021b	Eosinophilic (before propensity score matching)	Per patient	(9)			(1475)			
			Non-eosinophilic (before propensity score matching)	Per patient	(9)			(1606)			
			Eosinophilic (after propensity score matching)	Per patient	(9)			(1475)			
			Non-eosinophilic (after propensity score matching)	Per patient	(9)			(1629)			
		E W, 2021	AECOPD with pneumoconiosis	Per patient	16.9			2242			
			AECOPD without pneumoconiosis	Per patient	10.4			1535			
		Gong C, 2020	Serum procalcitonin levels ≥0.1 ng/ml	Per patient				4492			
			Serum procalcitonin levels <0.1 ng/ml	Per patient				3206			
		Liang L, 2020a	2009	Per hospitalization	16			3328 (2198)			
			2010	Per hospitalization	15.9			3574 (2327)			
			2011	Per hospitalization	15.4			3395 (2241)			
			2012	Per hospitalization	14.5			3341 (2284)			
			2013	Per hospitalization	14.1			3316 (2330)			
			2014	Per hospitalization	14.3			3358 (2352)			
			2015	Per hospitalization	14			3465 (2511)			
			2016	Per hospitalization	13.8			3464 (2526)			
			2017	Per hospitalization	13.5			3389 (2500)			
			2009	Per patient	16			3995 (2289)			
			2010	Per patient	15.9			4407 (2453)			
			2011	Per patient	15.4			4289 (2392)			
			2012	Per patient	14.5			4384 (2466)			

Income group	Country	Reference ID	Subgroup	Unit	Length of hospitalization stay (day)	Medication costs	Emergency visit costs	Hospitalization costs	Direct medical costs	Direct non-medical costs	Indirect costs
			2013	Per patient	14.1			4679 (2583)			
			2014	Per patient	14.3			5018 (2760)			
			2015	Per patient	14			5192 (2848)			
			2016	Per patient	13.8			5239 (2962)			
			2017	Per patient	13.5			5222 (2955)			
		Liang L, 2020b	2008	Per hospitalization	16.7 (13)			2687 (1832)			
			2009	Per hospitalization	16.2 (13)			3113 (2042)			
			2010	Per hospitalization	15.8 (13)			3366 (2193)			
			2011	Per hospitalization	15.4 (12)			3235 (2147)			
			2012	Per hospitalization	14.4 (12)			3198 (2196)			
			2013	Per hospitalization	14.1 (12)			3228 (2282)			
			2014	Per hospitalization	14.2 (12)			3282 (2311)			
			2015	Per hospitalization	13.9 (23)			3371 (2462)			
			2016	Per hospitalization	13.5 (12)			3406 (2489)			
			2017	Per hospitalization	13.2 (12)			3348 (2476)			
		Zhang W, 2017		Per patient	15.1	1385		2796			
	Malaysia	Binti Dzakwan NSA, 2017	<5 days length of stay	Per patient		199					
			5-10 days length of stay	Per patient		337					
			>10 days length of stay	Per patient		670					
	Russia	Vidyakina EE, 2016		Per patient	16	109		190			
	Serbia	Pavlovic R, 2020	Patients with frequent exacerbations	Per patient	13.7	231		432	1103		
			Patients with only one exacerbation over the course of one year	Per patient	10.6	182		326	957		
	Thailand	Pothirat C, 2015	Managed by pulmonologist	Per hospitalization	(3.5)	(240)		(689)			

Income group	Country	Reference ID	Subgroup	Unit	Length of hospitalization stay (day)	Medication costs	Emergency visit costs	Hospitalization costs	Direct medical costs	Direct non-medical costs	Indirect costs
			Managed by internist	Per hospitalization	(4.5)	(158)		(557)			
			Requiring mechanical ventilator managed by pulmonologist	Per hospitalization	(3)	(312)		(972)			
			Requiring mechanical ventilator managed by internist	Per hospitalization	(5.5)	(707)		(2360)			
		Reechaipichitkul W, 2014		Per hospitalization	17.3			2912			
		Inchai J, 2020	With comorbidities	Per patient	(7)			(1041)			
			Without comorbidities	Per patient	(5)			(640)			
	Turkey	Deniz S, 2016	The whole study population	Per exacerbation	8.1	131		191	475		
			Pneumonia present	Per exacerbation		135		282	696		
			Diabetes present	Per exacerbation		225		377	890		
			Heart failure present	Per exacerbation		220		452	1046		
			Hypertension present	Per exacerbation		247		450	1081		
			Coronary artery disease present	Per exacerbation		272		470	1101		
			Anemia present	Per exacerbation		287		531	1252		
			Reflux present	Per exacerbation		123		231	532		
			Lung cancer present	Per exacerbation		453		195	540		
			Pulmonary thromboemboli present	Per exacerbation		264		271	652		
			Arrhythmia present	Per exacerbation		195		312	768		
			Malnutrition present	Per exacerbation		114		208	504		
			Obesity present	Per exacerbation		185		205	561		
			Anxiety/depression present	Per exacerbation		182		272	657		
			Osteoporosis present	Per exacerbation		134		340	720		

Income group	Country	Reference ID	Subgroup	Unit	Length of hospitalization stay (day)	Medication costs	Emergency visit costs	Hospitalization costs	Direct medical costs	Direct non-medical costs	Indirect costs
			Any comorbidities present	Per exacerbation		153		243	596		
			Pneumonia absent	Per exacerbation		121		171	426		
			Diabetes absent	Per exacerbation		107		144	370		
			Heart failure absent	Per exacerbation		111		130	343		
			Hypertension absent	Per exacerbation		97		114	295		
			Coronary artery disease absent	Per exacerbation		97		124	325		
			Anemia absent	Per exacerbation		106		126	350		
			Reflux absent	Per exacerbation		132		185	467		
			Lung cancer absent	Per exacerbation		125		191	474		
			Pulmonary thromboemboli absent	Per exacerbation		129		190	472		
			Arrhythmiaabsent	Per exacerbation		127		183	455		
			Malnutrition absent	Per exacerbation		132		190	474		
			Obesity absent	Per exacerbation		130		191	473		
			Anxiety/depression absent	Per exacerbation		118		170	429		
			Osteoporosis absent	Per exacerbation		131		182	461		
			Comorbidities absent	Per exacerbation		70		46	137		
			Age <65	Per exacerbation		118		172	434		
			Age ≥65	Per exacerbation		135		196	487		
			Female	Per exacerbation		103		234	542		
			Male	Per exacerbation		136		183	462		
			Without intensive care stay	Per exacerbation		264		257	752		
			With intensive care stay	Per exacerbation	10.4	407		1255	2540		

Income group	Country	Reference ID	Subgroup	Unit	Length of hospitalization stay (day)	Medication costs	Emergency visit costs	Hospitalization costs	Direct medical costs	Direct non-medical costs	Indirect costs
			Without Intubation	Per exacerbation	10.4	264		299	843		
			With Intubation	Per exacerbation	10.4	664		2334	4207		
			Without non-invasive mechanical ventilation	Per exacerbation	10.4	277		373	939		
			With non-invasive mechanical ventilation	Per exacerbation	10.4	399		951	2231		
		Tanriverdi H, 2013		Per patient		130		367			
		Örnek T, 2014	AECOPD with coal worker pneumoconiosis	Per patient	13			1438			
			AECOPD without coal worker pneumoconiosis	Per patient	9.7			660			
		Varol Y, 2013		Per hospitalization	9.9 (8)	260		904			
		Yilmaz C, 2021	The index hospitalization	Per patient	8.1			456			
			Readmission	Per patient	8.3			682			
			Index hospitalitation without readmission	Per patient	7.8			334			

2.4 AECOPD, without intervention, by continent

Table S2.4 Cost of AECOPD by continent among non-intervention studies

Continent	Country	Reference ID	Subgroup	Unit	Length of hospitalization stay (day)	Medication costs	Emergency visit costs	Hospitalization costs	Direct medical costs	Direct non-medical costs	Indirect costs
Asia	China	Li F, 2018	Total study population	Per patient	8.5	1045		2012			
			High-cost group	Per patient	10.2			2818			
			Low-cost group	Per patient	6.9			1175			
			Length of stay in hospital <8 days	Per patient		675		1522			
			Length of stay in hospital ≥8 days	Per patient		1378		2423			
		Li M, 2018		Per patient per hospitalization	9.4 (7)	1898 (1010)		4171 (2517)			

Continent	Country	Reference ID	Subgroup	Unit	Length of hospitalization stay (day)	Medication costs	Emergency visit costs	Hospitalization costs	Direct medical costs	Direct non-medical costs	Indirect costs
		Mao X, 2021	The whole study population	Per patient				(1576)			
			With diabetes	Per patient				(1778)			
			Without diabetes	Per patient				(1551)			
		Wang S, 2021	The whole study population	Per patient	15.3			3859			
			Peripheral blood eosinophils <2%	Per patient	15.7			4191			
			Peripheral blood eosinophils ≥2%	Per patient	15			3315			
		Zhang J, 2018	Before disease assessment test	Per patient	12.6 (13)		16				
			Before disease assessment test	Per patient	18.5 (15)		57				
		Cui Y, 2022	AECOPD with coronary artery disease	Per patient	10			1571			
			AECOPD without coronary artery disease	Per patient	9			1496			
		Fan L, 2014	NPPV success group	Per patient	15.9			9970			
			NPPV failure group	Per patient	17.5			15442			
		You L, 2021	Without pulmonary heart disease	Per patient	(9)	(600)		(1557)			
			With pulmonary heart disease	Per patient	(10)	(618)		(1619)			
		Zeng Q, 2021	Total study population, eosinophilic group	Per patient	10.5			(1684)			
			Total study population, non-eosinophilic group	Per patient	11.9			(2072)			
			Eosinophilic group, aged >65	Per patient	10.7			(1819)			
			Non-eosinophilic group, aged >65	Per patient	11.9			(2116)			
			Eosinophilic group, aged 45-65	Per patient	9.1			(1255)			
			Non-eosinophilic group, aged 45-65	Per patient	11.4			(1631)			
		Chen R, 2020	High adherence group (before index), inhale bronchodilator	Per year per patient				1577 (717)			

Continent	Country	Reference ID	Subgroup	Unit	Length of hospitalization stay (day)	Medication costs	Emergency visit costs	Hospitalization costs	Direct medical costs	Direct non-medical costs	Indirect costs
			High adherence group (before index), combined	Per year per patient				1460 (624)			
			High adherence group (before index), oral therapy	Per year per patient				4070 (1025)			
			High adherence group (after index), inhale bronchodilator users	Per year per patient				1691			
			High adherence group (after index), combined	Per year per patient				1514			
			High adherence group (after index), oral therapy	Per year per patient				5732			
			Low adherence group (before index), inhale bronchodilator	Per year per patient				1232 (172)			
			Low adherence group (before index), combined	Per year per patient				1078 (243)			
			Low adherence group (before index), oral therapy	Per year per patient				1076 (80)			
			Low adherence group (after index), inhale bronchodilator	Per year per patient				2034			
			Low adherence group (after index), combined	Per year per patient				2437			
			Low adherence group (after index), oral therapy	Per year per patient				2232			
		Cui Y, 2021a	Group LL	Per patient	(10)			3049			
			Group LH	Per patient	(12)			4896			
			Group HL	Per patient	(10)			2485			
			Group HH	Per patient	(11)			2343			
		Cui Y, 2021b	Eosinophilic (before propensity score matching)	Per patient	(9)			(1475)			
			Non-eosinophilic (before propensity score matching)	Per patient	(9)			(1606)			

Continent	Country	Reference ID	Subgroup	Unit	Length of hospitalization stay (day)	Medication costs	Emergency visit costs	Hospitalization costs	Direct medical costs	Direct non-medical costs	Indirect costs
			Eosinophilic (after propensity score matching)	Per patient	(9)			(1475)			
			Non-eosinophilic (after propensity score matching)	Per patient	(9)			(1629)			
		E W, 2021	AECOPD with pneumoconiosis	Per patient	16.9			2242			
			AECOPD without pneumoconiosis	Per patient	10.4			1535			
		Gong C, 2020	Serum procalcitonin levels ≥0.1 ng/ml	Per patient				4492			
			Serum procalcitonin levels <0.1 ng/ml	Per patient				3206			
		Liang L, 2020a	2009	Per hospitalization	16			3328 (2198)			
			2010	Per hospitalization	15.9			3574 (2327)			
			2011	Per hospitalization	15.4			3395 (2241)			
			2012	Per hospitalization	14.5			3341 (2284)			
			2013	Per hospitalization	14.1			3316 (2330)			
			2014	Per hospitalization	14.3			3358 (2352)			
			2015	Per hospitalization	14			3465 (2511)			
			2016	Per hospitalization	13.8			3464 (2526)			
			2017	Per hospitalization	13.5			3389 (2500)			
			2009	Per patient	16			3995 (2289)			
			2010	Per patient	15.9			4407 (2453)			
			2011	Per patient	15.4			4289 (2392)			
			2012	Per patient	14.5			4384 (2466)			
			2013	Per patient	14.1			4679 (2583)			
			2014	Per patient	14.3			5018 (2760)			
			2015	Per patient	14			5192 (2848)			
			2016	Per patient	13.8			5239 (2962)			
			2017	Per patient	13.5			5222 (2955)			

Continent	Country	Reference ID	Subgroup	Unit	Length of hospitalization stay (day)	Medication costs	Emergency visit costs	Hospitalization costs	Direct medical costs	Direct non-medical costs	Indirect costs
		Liang L, 2020b	2008	Per hospitalization	16.7 (13)			2687 (1832)			
			2009	Per hospitalization	16.2 (13)			3113 (2042)			
			2010	Per hospitalization	15.8 (13)			3366 (2193)			
			2011	Per hospitalization	15.4 (12)			3235 (2147)			
			2012	Per hospitalization	14.4 (12)			3198 (2196)			
			2013	Per hospitalization	14.1 (12)			3228 (2282)			
			2014	Per hospitalization	14.2 (12)			3282 (2311)			
			2015	Per hospitalization	13.9 (23)			3371 (2462)			
			2016	Per hospitalization	13.5 (12)			3406 (2489)			
			2017	Per hospitalization	13.2 (12)			3348 (2476)			
		Zhang W, 2017		Per patient	15.1	1385		2796			
	India	Koul PA, 2019		Per exacerbation	(8)	(115)		(625)			
		Altaf M, 2015	Salmeterol/fluticasone	Per patient over 6 months	4.8						55
			Formoterol/budesonide	Per patient over 6 months	6						60
			Formoterol/fluticasone	Per patient over 6 months	7.4						67
	Iran	Torabipour A, 2016	2011	Per year per patient	8.5 (5)	120		370	632		
			2012	Per year per patient	8.5 (5)	301		761	1296		
			2013	Per year per patient	8.5 (5)	540		985	1893		
			Length of stay ≤ 9 days	Per patient	8.5 (5)	176 (90)		303 (156)	631 (371)		
			Length of stay > 9 days	Per patient	8.5 (5)	759 (438)		1942 (1273)	3232 (2344)		
			Age <65	Per patient	8.5 (5)				1037 (380)		
			Age >65	Per patient	8.5 (5)				1354 (621)		

Continent	Country	Reference ID	Subgroup	Unit	Length of hospitalization stay (day)	Medication costs	Emergency visit costs	Hospitalization costs	Direct medical costs	Direct non-medical costs	Indirect costs
			Female	Per patient	8.5 (5)				1640 (496)		
			Male	Per patient	8.5 (5)				1097 (506)		
	Malaysia	Binti Dzakwan NSA, 2017	<5 days length of stay	Per patient		199					
			5-10 days length of stay	Per patient		337					
			>10 days length of stay	Per patient		670					
	Pakistan	Iqbal MS, 2020b	Severity III	Per exacerbation	4.2	49					
			Severity II	Per exacerbation	9.2	62					
			Severity level I	Per exacerbation	13	81					
	Thailand	Pothirat C, 2015	Managed by pulmonologist	Per hospitalization	(3.5)	(240)		(689)			
			Managed by internist	Per hospitalization	(4.5)	(158)		(557)			
			Requiring mechanical ventilator managed by pulmonologist	Per hospitalization	(3)	(312)		(972)			
			Requiring mechanical ventilator managed by internist	Per hospitalization	(5.5)	(707)		(2360)			
		Reechaipichitkul W, 2014		Per hospitalization	17.3			2912			
		Inchai J, 2020	With comorbidities	Per patient	(7)			(1041)			
			Without comorbidities	Per patient	(5)			(640)			
	Turkey	Tanriverdi H, 2013		Per patient		130		367			
		Örnek T, 2014	AECOPD with coal worker pneumoconiosis	Per patient	13			1438			
			AECOPD without coal worker pneumoconiosis	Per patient	9.7			660			
		Yilmaz C, 2021	The index hospitalization	Per patient	8.1			456			
			Readmission	Per patient	8.3			682			
			Index hospitalitation without readmission	Per patient	7.8			334			

Continent	Country	Reference ID	Subgroup	Unit	Length of hospitalization stay (day)	Medication costs	Emergency visit costs	Hospitalization costs	Direct medical costs	Direct non-medical costs	Indirect costs
	Vietnam	Ngo CQ, 2019	The whole study population	Per patient	8.9	499		854			
			GOLD I	Per patient	5.3	126		299			
			GOLD II	Per patient	8.4	378		658			
			GOLD III	Per patient	9.3	537		914			
			GOLD IV	Per patient	9.3	579		975			
Eastern Europe	Russia	Vidyakina EE, 2016		Per patient	16	109		190			
	Serbia	Pavlovic R, 2020	Patients with frequent exacerbations	Per patient	13.7	231		432	1103		
			Patients with only one exacerbation over the course of one year	Per patient	10.6	182		326	957		
	Turkey	Deniz S, 2016	The whole study population	Per exacerbation	8.1	131		191	475		
			Pneumonia present	Per exacerbation		135		282	696		
			Diabetes present	Per exacerbation		225		377	890		
			Heart failure present	Per exacerbation		220		452	1046		
			Hypertension present	Per exacerbation		247		450	1081		
			Coronary artery disease present	Per exacerbation		272		470	1101		
			Anemia present	Per exacerbation		287		531	1252		
			Reflux present	Per exacerbation		123		231	532		
			Lung cancer present	Per exacerbation		453		195	540		
			Pulmonary thromboemboli present	Per exacerbation		264		271	652		
			Arrhythmia present	Per exacerbation		195		312	768		
			Malnutrition present	Per exacerbation		114		208	504		
			Obesity present	Per exacerbation		185		205	561		
			Anxiety/depression present	Per exacerbation		182		272	657		

Continent	Country	Reference ID	Subgroup	Unit	Length of hospitalization stay (day)	Medication costs	Emergency visit costs	Hospitalization costs	Direct medical costs	Direct non-medical costs	Indirect costs
			Osteoporosis present	Per exacerbation		134		340	720		
			Any comorbidities present	Per exacerbation		153		243	596		
			Pneumonia absent	Per exacerbation		121		171	426		
			Diabetes absent	Per exacerbation		107		144	370		
			Heart failure absent	Per exacerbation		111		130	343		
			Hypertension absent	Per exacerbation		97		114	295		
			Coronary artery disease absent	Per exacerbation		97		124	325		
			Anemia absent	Per exacerbation		106		126	350		
			Reflux absent	Per exacerbation		132		185	467		
			Lung cancer absent	Per exacerbation		125		191	474		
			Pulmonary thromboemboli absent	Per exacerbation		129		190	472		
			Arrhythmiaabsent	Per exacerbation		127		183	455		
			Malnutrition absent	Per exacerbation		132		190	474		
			Obesity absent	Per exacerbation		130		191	473		
			Anxiety/depression absent	Per exacerbation		118		170	429		
			Osteoporosis absent	Per exacerbation		131		182	461		
			Comorbidities absent	Per exacerbation		70		46	137		
			Age <65	Per exacerbation		118		172	434		
			Age ≥65	Per exacerbation		135		196	487		
			Female	Per exacerbation		103		234	542		
			Male	Per exacerbation		136		183	462		
			Without intensive care stay	Per exacerbation		264		257	752		

Continent	Country	Reference ID	Subgroup	Unit	Length of hospitalization stay (day)	Medication costs	Emergency visit costs	Hospitalization costs	Direct medical costs	Direct non-medical costs	Indirect costs
			With intensive care stay	Per exacerbation	10.4	407		1255	2540		
			Without Intubation	Per exacerbation	10.4	264		299	843		
			With Intubation	Per exacerbation	10.4	664		2334	4207		
			Without non-invasive mechanical ventilation	Per exacerbation	10.4	277		373	939		
			With non-invasive mechanical ventilation	Per exacerbation	10.4	399		951	2231		
		Varol Y, 2013		Per hospitalization	9.9 (8)	260		904			

2.5 CB, by income level and continent

Table S2.5 Cost of chronic bronchitis

Income group	Continent	Country	Reference ID	Subgroup	Unit	Length of hospitalization stay (day)	Medication costs	Emergency visit costs	Hospitalization costs	Direct medical costs	Direct non-medical costs	Indirect costs
Upper middle income	Asia	China	Xuan J, 2015	Broncho-Vaxom	Per acute exacerbation					146		
				Standard care therapy	Per acute exacerbation					121		
			Zhu YF, 2015	Cephalosporin combined with herba houttuyniae)	Per patient	26				1829		
				Conventional treatment with cephalosporin group	Per patient	32.4				2233		
	Eastern Europe	Russia	Ignatova GL, 2017	Vaccinated with pneumococcal 13-valent conjugate vaccine	Per year per patient							15
				Without vaccination	Per year per patient							46

2.6 Other disease, without intervention, by income level and continent

Table S2.6 Cost of other diseases among non-intervention studies

Income group	Continent	Country	Reference ID	Subgroup	Disease	Unit	Length of hospitalization stay (day)	Medication costs	Emergency visit costs	Hospitalization costs	Direct medical costs	Direct non-medical costs	Indirect costs
Low income	Africa	Malawi	Meghji J, 2020		Post-TB sequalae	Per hospitalization	(4)			(25)			
		Uganda	Settumba SN, 2015	Hospital	COPD/Asthma	Per outpatient visit		0.04					
				Health center level IV	COPD/Asthma	Per outpatient visit		0.08					
				Health center level III	COPD/Asthma	Per outpatient visit		0.61					
				Health center level II	COPD/Asthma	Per outpatient visit		0.44					
Upper middle income	Asia	China	Peng X, 2017		COPD/AECOPD	Per patient				2694			
		Jordan	Altawalbeh SM, 2021	Asthma and COPD overlap	Asthma-COPD overlap	Per patient				2085			

2.7 With intervention, by disease and country

Table S2.7 Cost of diseases among intervention studies

Disease	Country	Reference ID	Subgroup	Unit	Length of hospitalization stay (day)	Medication costs	Emergency visit costs	Hospitalization costs	Direct medical costs	Direct non-medical costs	Indirect costs
AECOPD	China	Qian W, 2016	CD64-guided group	Per patient per hospitalization	17.4			2139			
			Conventional treatment group	Per patient per hospitalization	12.6			3300			
		Zhang J, 2019	Any Nebulized budesonide (nBUD) regimen	Per patient	12.2 (11)			(2092)			

Disease	Country	Reference ID	Subgroup	Unit	Length of hospitalization stay (day)	Medication costs	Emergency visit costs	Hospitalization costs	Direct medical costs	Direct non-medical costs	Indirect costs
			Any systemic corticosteroids (SCS) regimen: SCS monotherapy, sequential therapy (ie, initiated with SCS at hospitalization and later switched to nBUD), and sequential-combination therapy (initiated with SCS at hospitalization and later combined with nBUD)	Per patient	12.5 (12)			(2152)			
			nBud monotherapy	Per patient	11.4 (10)			(1930)			
			SCS monotherapy	Per patient	12 (11)			(2051)			
		Zheng JP, 2019	Dose escalation group (NBS initial) - initial NBS followed by dose escalation or switch to SCS	Per patient per hospitalization	13.9			3365			
			Dose maintenance or reduction group (NBS initial) - initial NBS followed by dose maintenance or reduction	Per patient per hospitalization	11.7			2405			
			Dose maintenance or escalation group (SCS initial) - initial SCS followed by dose maintenance or escalation	Per patient per hospitalization	11.3			2683			
			Dose reduction group (SCS intial) - initial SCS followed by dose reduction or switch to NBS	Per patient per hospitalization	13.3			2808			
		Chen Y, 2020	Nebulized corticosteroids (NCS)	Per year per patient	11.8			2597			
			Oral or intravenous systemic corticosteroids (SCS)	Per patient	12			2588			
			Both nebulized budesonide, and oral or intravenous sstemic corticosteroids sequentially or con-currently	Per patient	13.3			3170			
		Lan B, 2013	Physiological saline group	Per patient per day	12.2			984			
			Bromhexine group	Per patient per day	11.4			982			
			Ambroxol group	Per patient per day	10.9			972			
		Lin S-H, 2021	Procalcitonin Kinetis guided sequential invasive-noninvasive	Per patient	8.7			1812			

Disease	Country	Reference ID	Subgroup	Unit	Length of hospitalization stay (day)	Medication costs	Emergency visit costs	Hospitalization costs	Direct medical costs	Direct non-medical costs	Indirect costs
			mechanical ventilation weaning group								
			Pulmonary infection control window guided sequential invasive-noninvasive mechanical ventilation weaning group	Per patient	11.5			2441			
		Shang D, 2014	Non-invasive positive pressure ventilation group	Per patient	7.5			3535			
			Control group	Per patient	10.6			2152			
		Tan J, 2014	State machine-based clinical pathway management group	Per patient	11.1			1785			
			Tranditional clinical pathway management group	Per patient	12.9			2040			
		Wu L, 2021	Glucocorticoid use group	Per year per patient	(13)			1423			
			No glucocorticoid use group	Per year per patient	(11)			1290			
		Zeng X-B, 2017	Serum procalcitonin level-based approach to use and end the timing of antibiotic treatment	Per patient				1320			
			Patient's clinical symptoms, signs and clinical judgment of the doctor-based approach to use and end the timing of antibiotic treatment	Per patient				1516			
		Zhang J, 2020	Early enteral nutrition standardized treatment process management group	Per patient	4.9			3449			
			Routine enteral nutrition standardize treatment process management group	Per patient	5.2			3537			
	Russia	Astef'ev AV, 2013	Hyleflox-750	Per patient		19					
		Ignatova GL, 2016	Without vaccination, 2012	Per patient over 1 year				353			
			Without vaccination, 2013	Per patient over 2 years				706			
			Without vaccination, 2014	Per patient over 3 years				1058			
			Vaccinated with 13-valent vonjugate pneumococcal vaccine Prevenar 13, 2012	Per patient over 1 year				69			

Disease	Country	Reference ID	Subgroup	Unit	Length of hospitalization stay (day)	Medication costs	Emergency visit costs	Hospitalization costs	Direct medical costs	Direct non-medical costs	Indirect costs
			Vaccinated with 13-valent vonjugate pneumococcal vaccine Prevenar 13, 2013	Per patient over 2 years				161			
			Vaccinated with 13-valent vonjugate pneumococcal vaccine Prevenar 13, 2014	Per patient over 3 years				436			
			Vaccinated with polyvalent pneumococcal vaccine Pneumo 23, 2012	Per patient over 1 year				57			
			Vaccinated with polyvalent pneumococcal vaccine Pneumo 23, 2013	Per patient over 2 years				104			
			Vaccinated with polyvalent pneumococcal vaccine Pneumo 23, 2014	Per patient over 3 years				161			
Chronic bronchitis	China	Xuan J, 2015	Broncho-Vaxom (OM-85) in managing respiratory tract infections	Per acute exacerbation					146		
			Standard care therapy	Per acute exacerbation					121		
		Zhu YF, 2015	Treatment group (with cephalosporin combined with herba houttuyniae)	Per patient	26				1829		
			Conventional treatment with cephalosporin group	Per patient	32.4				2233		
	Russia	Ignatova GL, 2017	With vaccination of pneumococcal 13-valent conjugate vaccine	Per year per patient							15
			Without vaccination	Per year per patient							46
COPD	Brazil	Farias C C, 2014	Without aerobic walking program	Per patient			3	185			
			With aerobic walking program	Per patient			0	0			
	China	Bao H, 2017	Community physician-guided long-term domiciliary oxygen therapy combined with conventional therapy group - before treatment	Per year per patient			2788	9910			
			Community physician-guided long-term domiciliary oxygen therapy combined with conventional therapy group - after treatment	Per year per patient			2046	7502			

Disease	Country	Reference ID	Subgroup	Unit	Length of hospitalization stay (day)	Medication costs	Emergency visit costs	Hospitalization costs	Direct medical costs	Direct non-medical costs	Indirect costs
			Conventional therapy group - before treatment	Per year per patient			2728	9729			
			Conventional therapy group - after treatment	Per year per patient			2708	9849			
		Liu M, 2021	Clinical pharmacist-led medication therapy management combined with the conventional treatment	Per patient	11.3			2099			
			Conventional treatment	Per patient per hospitalization	13.5			2326			
		Qu S, 2021	No screening/usual care	Lifetime costs per patient					4080		
			Questionnaire screening	Lifetime costs per patient					4011		
			Portable spirometer screening	Lifetime costs per patient					3973		
		Shi M, 2018	Before adoption of integrated case payment in both township and county hospitals	Per patient	8.1				253		
			Before adoption of integrated case payment in township hospitals	Per patient	8.1				218		
			Before adoption of integrated case payment in county hospitals	Per patient	8.2				440		
			After adoption of integrated case payment in township hospitals	Per patient	12.1				279		
			After adoption of integrated case payment in county hospitals	Per patient	11.7				847		
			After adoption of integrated case payment in both township and county hospitals	Per patient	11.9				451		
		Gong S, 2021	Indacaterol/glycopyrronium versus treatment with salmeterol/fluticasone cohort - treatment with indacaterol/glycopyrronium	Lifetime costs per patient					12411		
			Indacaterol/glycopyrronium versus treatment with salmeterol/fluticasone cohort - treatment with salmeterol/fluticasone	Lifetime costs per patient					14616		

Disease	Country	Reference ID	Subgroup	Unit	Length of hospitalization stay (day)	Medication costs	Emergency visit costs	Hospitalization costs	Direct medical costs	Direct non-medical costs	Indirect costs
			Indacaterol/glycopyrronium versus tiotropium cohort - treatment with indacaterol/glycopyrronium	Lifetime costs per patient					13749		
			Indacaterol/glycopyrronium versus tiotropium cohort - treatment with tiotropium	Lifetime costs per patient					12273		
		Li P, 2015	Proposed new mode of community continuing nusing care: being followed up with by a specialist nurse after COPD-associated hospitalization	Per patient					821 (188)		
			Conventional nursing care: not being followed up with by a specialist nurse after COPD-associated hospitalization	Per patient					1394 (1606)		
		Wang L, 2022	Treatment with glycopyrrolate/formoterol	Lifetime costs per patient					4319		
			Treatment with tiotropium bromide	Lifetime costs per patient					5146		
		Ye X, 2021	Patients not receiving the COPD risk forecasting service	Per patient		66					
			Patients receiving the COPD risk forecasting service	Per patient		62					
		Zhou Y, 2021	Treatment with fluticasone/umeclidinium/vilanterol triple combination (FF/UMEC/VI)	Per patient over 14 years					31280		
			Treatment with budesonide/formoterol double combination (BUD/FOR)	Per patient over 14 years					27094		
		Qiu Y, 2016	Before 23 value pneumococcal polysaccharide vaccination	Per patient				892	1011		
			After 23 value pneumococcal polysaccharide vaccination	Per patient				308	384		
		Tan J, 2018	Home atomization inhalation in treatment of acute attack of COPD	Per year per patient					219		
			Pharmacotherapy or short-term atomization inhalation in outpatient clinic	Per year per patient					212		
		Wang J, 2020	After zero mark-up drug policy	Per patient per month				2427			

Disease	Country	Reference ID	Subgroup	Unit	Length of hospitalization stay (day)	Medication costs	Emergency visit costs	Hospitalization costs	Direct medical costs	Direct non-medical costs	Indirect costs
			Before zero mark-up drug policy	Per patient per month				2851			
		Xu Z, 2021	Before outpatient oxygen therapy	Per patient over 6 months					2030 (1504)		
			After outpatient oxygen therapy	Per patient over 6 months					1887 (1202)		
		Zhang A, 2020	Treatment with usual care (base line - 12 months before intervention)	Per year per patient	(16)		(0)	(2128)	(2502)		
			Treatment with usual care (after 12 months)	Per year per patient	(17)		(81)	(2000)	(2432)		
			Treatment with usual care (after 12-24 months	Per year per patient	(13)		(94)	(1749)	(2136)		
			Hospital-outreach pulmonary rehabilitation (base line - 12 months before intervention)	Per year per patient	(16)		(86)	(1912)	(2328)		
			Hospital-outreach pulmonary rehabilitation (after 12 months)	Per year per patient	(8)		(0)	(1385)	(1711)		
			Hospital-outreach pulmonary rehabilitation (after 12-24 months)	Per year per patient	(7)		(70)	(227)	(1328)		
	Colombia	Estrada JI, 2015	Before one-year pharmacotherapeutic follow-up program	Per month per patient					(116)		
			After one-year pharmacotherapeutic follow-up program	Per month per patient					(103)		
	India	Abdulsalim S, 2019	Normal care (GOLD I)	Per patient over 6 months		(45)					
			Normal care (GOLD II)	Per patient over 6 months		(46)					
			Normal care (GOLD III)	Per patient over 6 months		(63)					
			Normal care (GOLD IV)	Per patient over 6 months		(73)					
			Structured pharmacist-led intervention (GOLD I)	Per patient over 6 months		(34)					

Disease	Country	Reference ID	Subgroup	Unit	Length of hospitalization stay (day)	Medication costs	Emergency visit costs	Hospitalization costs	Direct medical costs	Direct non-medical costs	Indirect costs
			Structured pharmacist-led intervention (GOLD II)	Per patient over 6 months		(38)					
			Structured pharmacist-led intervention (GOLD III)	Per patient over 6 months		(47)					
			Structured pharmacist-led intervention (GOLD IV)	Per patient over 6 months		(51)					
	Iran	Piroozi B, 2019	Before health transformation plan intervention (2014)	Per hospitalization				1959			
			After health transformation plan intervention (2015)	Per hospitalization				1373			
	Romania	Beres E, 2021	Long-term oxygen therapy + non-invasive ventilation	Per patient					5303		
			Long-term oxygen therapy	Per patient					5788		
	Russia	Ignatova GL, 2015	COPD combined with coronary hear disease without vaccination after 1 year	Per patient over 1 year				313			
			COPD combined with coronary hear disease without vaccination after 1.5 year	Per patient over 2 years				625			
			COPD combined with coronary hear disease with vaccination after 1 year	Per patient over 1 year				44			
			COPD combined with coronary hear disease with vaccination after 1.5 year	Per patient over 2 years				83			
			COPD without coronary hear disease without vaccination after 1 year	Per patient over 1 year				140			
			COPD without coronary hear disease without vaccination after 1.5 year	Per patient over 2 years				280			
			COPD without coronary hear disease with vaccination after 1 year	Per patient over 1 year				26			
			COPD without coronary hear disease with vaccination after 1.5 year	Per patient over 2 years				53			

Disease	Country	Reference ID	Subgroup	Unit	Length of hospitalization stay (day)	Medication costs	Emergency visit costs	Hospitalization costs	Direct medical costs	Direct non-medical costs	Indirect costs
		Ishmurzin GP, 2016		Per patient per event			5	144	149		
		Titova ON, 2014	COPD patients not receiving assistive oxygen therapy	Per year per patient					1091		
		Balunov PA, 2018	Tiotropium-nativ	Per patient over 24 weeks					150		
			Spiriva	Per patient over 24 weeks					191		
			Ipraterol- nativ	Per year per patient					51		
			Berodual	Per year per patient					59		

2.8 Costs stratified by sex, without intervention

Table S2.8 Costs stratified by sex among observation studies

Disease	Country	Reference ID	Subgroup	Unit	Length of hospitalization stay (day)	Medication costs	Emergency visit costs	Hospitalization costs	Direct medical costs	Direct non-medical costs	Indirect costs
AECOPD	Iran	Torabipour A, 2016	Female	Per patient	8.5 (5)				1640 (496)		
			Male	Per patient	8.5 (5)				1097 (506)		
	Turkey	Deniz S, 2016	Female	Per exacerbation		103		234	542		
			Male	Per exacerbation		136		183	462		

2.9 Costs stratified by age, without intervention

Table S2.9 Costs stratified by age among observation studies

Disease	Country	Reference ID	Subgroup	Unit	Length of hospitalization stay (day)	Medication costs	Emergency visit costs	Hospitalization costs	Direct medical costs	Direct non-medical costs	Indirect costs
AECOPD	China	Zeng Q, 2021	Eosinophilic group, aged >65	Per patient	10.7			(1819)			

Disease	Country	Reference ID	Subgroup	Unit	Length of hospitalization stay (day)	Medication costs	Emergency visit costs	Hospitalization costs	Direct medical costs	Direct non-medical costs	Indirect costs
			Non-eosinophilic group, aged >65	Per patient	11.9			(2116)			
			Eosinophilic group, aged 45-65	Per patient	9.1			(1255)			
			Non-eosinophilic group, aged 45-65	Per patient	11.4			(1631)			
	Iran	Torabipour A, 2016	Age <65	Per patient	8.5 (5)				1037 (380)		
			Age >65	Per patient	8.5 (5)				1354 (621)		
	Turkey	Deniz S, 2016	Age <65	Per exacerbation		118		172	434		
			Age ≥65	Per exacerbation		135		196	487		
COPD	China	Liu H, 2018	2005, age 18-35	Per hospitalization	11.4			1133			
			2005, age 36-60	Per hospitalization	12.2			1209			
			2005, age >61	Per hospitalization	12.6			1255			
			2006, age 18-35	Per hospitalization	10.1			1042			
			2006, age 36-60	Per hospitalization	12.4			1022			
			2006, age >61	Per hospitalization	12.7			1235			
			2007, age 18-35	Per hospitalization	12.2			1229			
			2007, age 36-60	Per hospitalization	12.7			1125			
			2007, age >61	Per hospitalization	12.6			1299			
			2008, age 18-35	Per hospitalization	10.6			1366			
			2008, age 36-60	Per hospitalization	12.3			1026			
			2008, age >61	Per hospitalization	12.3			1373			

Disease	Country	Reference ID	Subgroup	Unit	Length of hospitalization stay (day)	Medication costs	Emergency visit costs	Hospitalization costs	Direct medical costs	Direct non-medical costs	Indirect costs
			2009, age 18-35	Per hospitalization	10.2			1435			
			2009, age 36-60	Per hospitalization	12.2			1255			
			2009, age >61	Per hospitalization	12.1			1607			
			2010, age 18-35	Per hospitalization	10.2			1533			
			2010, age 36-60	Per hospitalization	12.1			1322			
			2010, age >61	Per hospitalization	11.8			1658			
			2011, age 18-35	Per hospitalization	9.3			1422			
			2011, age 36-60	Per hospitalization	12			1202			
			2011, age >61	Per hospitalization	11.6			1565			
			2012 age 18-35	Per hospitalization	9.1			1630			
			2012, age 36-60	Per hospitalization	11.6			1328			
			2012, age >61	Per hospitalization	11.4			1603			
			2013, age 18-35	Per hospitalization	9.5			1767			
			2013, age 36-60	Per hospitalization	10.9			1767			
			2013, age >61	Per hospitalization	11.2			1852			
			2014, age 18-35	Per hospitalization	9.6			2065			
			2014, age 36-60	Per hospitalization	10.7			1442			
			2014, age >61	Per hospitalization	11.1			1778			
			2015, age 18-35	Per hospitalization	8.6			2126			
			2015, age 36-60	Per hospitalization	10.3			1441			
			2015, age >61	Per hospitalization	10.7			1716			

2.10 Cost stratified by disease severity, without intervention

Table S2.10 Costs stratified by disease severity among observation studies

Disease	Country	Reference ID	Subgroup	Unit	Length of hospitalization stay (day)	Medication costs	Emergency visit costs	Hospitalization costs	Direct medical costs	Direct non-medical costs	Indirect costs
AECOPD	Pakistan	Iqbal MS, 2020b	Severity III	Per exacerbation	4.2	49					
			Severity II	Per exacerbation	9.2	62					
			Severity level I	Per exacerbation	13	81					
	Vietnam	Ngo CQ, 2019	GOLD I	Per patient	5.3	126		299			
			GOLD II	Per patient	8.4	378		658			
			GOLD III	Per patient	9.3	537		914			
			GOLD IV	Per patient	9.3	579		975			
COPD	Bulgaria	Kamusheva M, 2017	GOLD I	Per year per patient							767
			GOLD II	Per year per patient				(288)			924
			GOLD III	Per year per patient				(482)			
			GOLD IV	Per year per patient				(602)			1800
	Mexico	Fernández-Plata R, 2016	Mild COPD	Per year per patient		(913)					(41)
			Moderate COPD	Per year per patient		(1031)					(66)
			Severe COPD	Per year per patient		(1136)					(48)
			Very severe COPD	Per year per patient		(1274)					(48)
		Nevárez-Aids A, 2017	Moderate COPD	Per year per patient		329	155	3240	1335		
			Severe COPD	Per year per patient		845	148	5775	2694		
	Thailand	Samarnkondsak T, 2019	Group 1 (mild + moderate, FEV1>=50%)	Per year per patient	0.5	305 (305)			428 (428)		
			Group 2 (severe + very severe, FEV1<50%)	Per year per patient	2.7	594 (584)			732 (736)		

Disease	Country	Reference ID	Subgroup	Unit	Length of hospitalization stay (day)	Medication costs	Emergency visit costs	Hospitalization costs	Direct medical costs	Direct non-medical costs	Indirect costs
	Vietnam	Vu TQ, 2019	Outpatient (GOLD III)	Per patient		31			35	14	35
			Outpatient (GOLD IV)	Per patient		42			47	15	33

2.11 Costs stratified by comorbidity, without intervention

Table S2.11 Costs stratified by comorbidity among observation studies

Disease	Country	Reference ID	Subgroup	Unit	Length of hospitalization stay (day)	Medication costs	Emergency visit costs	Hospitalization costs	Direct medical costs	Direct non-medical costs	Indirect costs
AECOPD	China	Cui Y, 2022	AECOPD with coronary artery disease	Per patient	10			1571			
			AECOPD without coronary artery disease	Per patient	9			1496			
		You L, 2021	Without pulmonary heart disease	Per patient	(9)	(600)		(1557)			
			With pulmonary heart disease	Per patient	(10)	(618)		(1619)			
		E W, 2021	AECOPD with pneumoconiosis	Per patient	16.9			2242			
			AECOPD without pneumoconiosis	Per patient	10.4			1535			
	Thailand	Inchai J, 2020	With comorbidities	Per patient	(7)			(1041)			
			Without comorbidities	Per patient	(5)			(640)			
	Turkey	Deniz S, 2016	Pneumonia present	Per exacerbation		135		282	696		
			Diabetes present	Per exacerbation		225		377	890		
			Heart failure present	Per exacerbation		220		452	1046		
			Hypertension present	Per exacerbation		247		450	1081		
			Coronary artery disease present	Per exacerbation		272		470	1101		
			Anemia present	Per exacerbation		287		531	1252		

Disease	Country	Reference ID	Subgroup	Unit	Length of hospitalization stay (day)	Medication costs	Emergency visit costs	Hospitalization costs	Direct medical costs	Direct non-medical costs	Indirect costs
			Reflux present	Per exacerbation		123		231	532		
			Lung cancer present	Per exacerbation		453		195	540		
			Pulmonary thromboemboli present	Per exacerbation		264		271	652		
			Arrhythmia present	Per exacerbation		195		312	768		
			Malnutrition present	Per exacerbation		114		208	504		
			Obesity present	Per exacerbation		185		205	561		
			Anxiety/depression present	Per exacerbation		182		272	657		
			Osteoporosis present	Per exacerbation		134		340	720		
			Any comorbidities present	Per exacerbation		153		243	596		
			Pneumonia absent	Per exacerbation		121		171	426		
			Diabetes absent	Per exacerbation		107		144	370		
			Heart failure absent	Per exacerbation		111		130	343		
			Hypertension absent	Per exacerbation		97		114	295		
			Coronary artery disease absent	Per exacerbation		97		124	325		
			Anemia absent	Per exacerbation		106		126	350		
			Reflux absent	Per exacerbation		132		185	467		
			Lung cancer absent	Per exacerbation		125		191	474		
			Pulmonary thromboemboli absent	Per exacerbation		129		190	472		
			Arrhythmiaabsent	Per exacerbation		127		183	455		
			Malnutrition absent	Per exacerbation		132		190	474		
			Obesity absent	Per exacerbation		130		191	473		

Disease	Country	Reference ID	Subgroup	Unit	Length of hospitalization stay (day)	Medication costs	Emergency visit costs	Hospitalization costs	Direct medical costs	Direct non-medical costs	Indirect costs
			Anxiety/depression absent	Per exacerbation		118		170	429		
			Osteoporosis absent	Per exacerbation		131		182	461		
			Comorbidities absent	Per exacerbation		70		46	137		
		Örnek T, 2014	AECOPD with coal worker pneumoconiosis	Per patient	13			1438			
			AECOPD without coal worker pneumoconiosis	Per patient	9.7			660			
COPD	China	Yang C, 2022	With chronic kidney disease	Per patient	(12)			(2166)			
			Without chronic kidney disease	Per patient	(10)			(1805)			
	Russia	Akramova EG, 2014	With comorbidities	Per year per patient		13					
			COPD and CVD	Per year per patient		33					
			CPD+ hypertension	Per year per patient		19					
