# Supplementary material for: Economic burden of chronic obstructive pulmonary disease and post-tuberculosis sequelae in low- and middle-income countries: a database compiled from a systematic review and meta-analysis
Source: BMJ Public Health. 2024 Jul 30;2(1):e000441. doi: 10.1136/bmjph-2023-000441 (PMC11816951; doi:10.1136/bmjph-2023-000441)
Supplement: online supplemental file 3 [file bmjph-2-1-s003.pdf]

# The economic burden of chronic obstructive pulmonary disease and post-tuberculosis sequelae in low- and middle-income countries: a database compiled from a systematic review and meta-analysis

**Yuling Lin[1], Alexandra Walker[2,3], Marguerite Batta[2,3], Sierra Ottilie-Kovelman[2,4], Anna Duchenko[2,3], Curdin Brugger[2,3], Olivia Keiser[1], Robert S. Wallis[5], Klaus Reither[2,3], Fabrizio Tediosi[2,3] , Marina Antillon[2,3*]**

1 Institute of Global Health, University of Geneva, 1205 Geneva, Switzerland
2 Swiss Tropical and Public Health Institute (Swiss TPH), Kreuzstrasse 2, 4123 Allschwil, Switzerland
3 University of Basel, Peterspl. 1, 4001 Basel, Switzerland
4 Yale School of Public Health, New Haven, Connecticut 06510, United States
5 The Aurum Institute, Johannesburg, South Africa
*Corresponding author

Supplemental Tables of Economic and Disease Burden Data

## Contents

3-I Table of economic burden data ..... 1
3.1 COPD, national, by income level ..... 1
3.2 COPD, national + city or region, by income level ..... 6
3.3 Chronic bronchitis, national + city or region, by income level ..... 22
3.4 Other diseases, national + city or region, by income level ..... 25
3-II Table of disease burden ..... 26
3.5 COPD, national, by income level ..... 27
3.6 COPD, national + city and region, by income level ..... 28
3.7 Chronic bronchitis, national + city and region, by income level ..... 31
3.8 Other diseases, national + city and region, by income level ..... 32

## 3-I Table of economic burden data

Costs are expressed in terms of millions of 2021 USD values. These means and medians are expressed by placing the value in parentheses [mean (median)]. * denotes that data are presented in absolute values and not in millions of dollars, and DALY denotes disability-adjusted life year.

### 3.1 COPD, national, by income level

**Table S3.1** Economic burden of COPD at national level

| Attribution | Income group | Country | Reference ID | Subgroup | Attribution (detail) | Unit | Medication | Hospitalization | Direct medical costs | Direct non-medical costs | Direct costs | Indirect costs | Mortality costs | Morbidity costs | DALY costs | Total costs |
|---|---|---|---|---|---|---|---|---|---|---|---|---|---|---|---|---|
| Air pollution | Lower middle income | Nigeria | Etchie TO, 2018 | 2005 | Ambient $PM_{2.5}$ | Per year | | | | | | | 802.4 | | | |
| | | | | 2015 | Ambient $PM_{2.5}$ | Per year | | | | | | | 717.3 | | | |
| | Upper middle income | China | Maji KJ, 2017c | COPD | $PM_{10}$ | Per year | | 515.6 | | | | | | | | |
| | | Thailand | Mueller W, 2021 | | $PM_{2.5}$ | During 1996 - 2016 period | | | | | | | 6903.9 | | 565.3 | |
| No attribution | Lower middle income | Iran | Rezaei S, 2017 | Overall burden | Not applicable | Per year | | 111.6 | | 236.9 | | | 40.8 | 11.1 | | |
| | Upper middle income | Bulgaria | Tachkov K, 2019 | 2013 | Not applicable | Per year | | 3.5 | | | | | | | | |
| | | | | 2016 | Not applicable | Per year | | 3.9 | | | | | | | | |
| | | | Tachkov K, 2018 | Smokers (among the cohort population) | Not applicable | Over 10 years | | | | | | | | | | 1 |
| | | | | Non-smokers or ex-smokers (among the | Not applicable | Over 10 years | | | | | | | | | | 1.3 |

| Attribution | Income group | Country | Reference ID | Subgroup | Attribution (detail) | Unit | Medication | Hospitalization | Direct medical costs | Direct non-medical costs | Direct costs | Indirect costs | Mortality costs | Morbidity costs | DALY costs | Total costs |
|---|---|---|---|---|---|---|---|---|---|---|---|---|---|---|---|---|
| | | | | cohort population) | | | | | | | | | | | | |
| | | | | Smokers (the entire Bulgarian population) | Not applicable | Over 10 years | | | | | | | | | | 762.1 |
| | | | | Non-smokers or ex-smokers (the entire Bulgarian population) | Not applicable | Over 10 years | | | | | | | | | | 404.1 |
| | | China | Salem A, 2021 | Current scenario, representativ eof the current treatment paradigm, in which only some (i.e., 38.50%) of the patients with COPD are treated with long-acting maintenance therapy after hospital charge | Not applicable | Per year | | | 3040.9 | | | | | | | |
| | | | | Future (hypothetical) scenario in which all patients with COPD are treated with long-acting maintenance therapy after hospitalisation due to a | Not applicable | Per year | | | 2445.5 | | | | | | | |

| Attribution | Income group | Country | Reference ID | Subgroup | Attribution (detail) | Unit | Medication | Hospitalization | Direct medical costs | Direct non-medical costs | Direct costs | Indirect costs | Mortality costs | Morbidity costs | DALY costs | Total costs |
|---|---|---|---|---|---|---|---|---|---|---|---|---|---|---|---|---|
| | | | | severe exacerbation | | | | | | | | | | | | |
| | | Paraguay | Bardach A, 2018 | Overall burden | Not applicable | Per year | | | 108.1 | | | | | | | |
| | | Peru | Bardach AE, 2016 | Overall burden | Not applicable | Per year | | | | | 294.3 | | | | | |
| | | Russia | Kontsevaia AV, 2019 | | Not applicable | Per year | | | 97.7 | 5.7 | 103.4 | 2739.1 | 2668.8 | | | 2842.5 |
| | | | Sapunova ID, 2019 | Overall burden | Not applicable | Per year | | | | | 103.5 | 2705.2 | 2705.2 | | | 2808.6 |
| | | | Zyryanov SK, 2018 | Single bronchodilator use, 1st year | Not applicable | Per year | | | 2.2 | | | | | | | |
| | | | | Single bronchodilator use, 2nd year | Not applicable | Per year | | | 2.2 | | | | | | | |
| | | | | Single bronchodilator use, 3rd year | Not applicable | Per year | | | 2.1 | | | | | | | |
| | | | | Dual bronchodilators (glycopyrronium bromide + indacaterol) use, 1st year | Not applicable | Per year | | | 1.8 | | | | | | | |
| | | | | Dual bronchodilators (glycopyrronium bromide + indacaterol) use, 2nd year | Not applicable | Per year | | | 1.7 | | | | | | | |
| | | | | Dual bronchodilators | Not applicable | Per year | | | 1.7 | | | | | | | |

| Attribution | Income group | Country | Reference ID | Subgroup | Attribution (detail) | Unit | Medication | Hospitalization | Direct medical costs | Direct non-medical costs | Direct costs | Indirect costs | Mortality costs | Morbidity costs | DALY costs | Total costs |
|---|---|---|---|---|---|---|---|---|---|---|---|---|---|---|---|---|
| | | | | (glycopyrronium bromide + indacaterol) use, 3rd year | | | | | | | | | | | | |
| | | Turkey | Ozdemir T, 2021 | 2012 | Not applicable | Per year | | | 274.6 | | | | | | | |
| | | | | 2013 | Not applicable | Per year | | | 274.2 | | | | | | | |
| | | | | 2014 | Not applicable | Per year | | | 284.8 | | | | | | | |
| | | | | 2015 | Not applicable | Per year | | | 278.4 | | | | | | | |
| | | | | 2016 | Not applicable | Per year | | | 285.4 | | | | | | | |
| Not applicable | | Thailand | Patanavanich R, 2018 | COPD | Not applicable | Over period from 2007 to 2014 | | 1404.3 | | | | | | | | |
| | | | | COPD with TB diagnosis | Not applicable | Over period from 2007 to 2014 | | 31.8 | | | | | | | | |
| Smoking | Lower middle income | Bolivia | Pichon-Riviere A, 2016 | Bolivia | Smoking | Per year | | | 39.4 | | | | | | | |
| | | | Pichon-Riviere A, 2020 | Bolivia | Smoking | Per year | | | 39.8 | | | | | | | |
| | | Honduras | | Honduras | Smoking | Per year | | | 15.5 | | | | | | | |
| | | Indonesia | Kristina SA, 2018 | COPD, male | Smoking | Per year | | | 789.6 | | | | | | | |
| | | | | COPD, female | Smoking | Per year | | | 2.1 | | | | | | | |

| Attribution | Income group | Country | Reference ID | Subgroup | Attribution (detail) | Unit | Medication | Hospitalization | Direct medical costs | Direct non-medical costs | Direct costs | Indirect costs | Mortality costs | Morbidity costs | DALY costs | Total costs |
|---|---|---|---|---|---|---|---|---|---|---|---|---|---|---|---|---|
| | | Iran | Rezaei S, 2017 | Smoking-attributable | Smoking | Per year | | 18 | | 37.8 | | | 145 | 11.1 | | 228.7 |
| | | Vietnam | Anh PTH, 2016 | | Smoking | Per year | | | | | 401.9 | 105.8 | 102.4 | 345818* | | 507.7 |
| | Upper middle income | Brazil | Pichon-Riviere A, 2016 | Brazil | Smoking | Per year | | | 3035.1 | | | | | | | |
| | | | Pichon-Riviere A, 2020 | Brazil | Smoking | Per year | | | 4004.2 | | | | | | | |
| | | Colombia | Pichon-Riviere A, 2016 | Columbia | Smoking | Per year | | | 376.5 | | | | | | | |
| | | | Pichon-Riviere A, 2020 | Colombia | Smoking | Per year | | | 377.4 | | | | | | | |
| | | Costa Rica | | Costa Rica | Smoking | Per year | | | 37.6 | | | | | | | |
| | | Ecuador | | Ecuador | Smoking | Per year | | | 210.1 | | | | | | | |
| | | Mexico | Pichon-Riviere A, 2016 | Mexico | Smoking | Per year | | | 1639.2 | | | | | | | |
| | | | Pichon-Riviere A, 2020 | Mexico | Smoking | Per year | | | 1638.2 | | | | | | | |
| | | Paraguay | Bardach A, 2018 | Attributable to smoking | Smoking | Per year | | | 79.9 | | | | | | | |
| | | | Pichon-Riviere A, 2020 | Paraguay | Smoking | Per year | | | 79.8 | | | | | | | |
| | | Peru | Pichon-Riviere A, 2016 | Peru | Smoking | Per year | | | 226.2 | | | | | | | |
| | | | Bardach AE, 2016 | Attributable to smoking | Smoking | Per year | | | | | 226.1 | | | | | |
| | | | Pichon-Riviere A, 2020 | Peru | Smoking | Per year | | | 227 | | | | | | | |
| | | Russia | Yagudra, 2018 | 2009 | Smoking | Per year | | | 501.2 | | | | 378.6 | | | |
| | | | | 2009-2016 | Smoking | Per year | | | 2329.2 | | | | 106 | | | |
| | | | Sapunova ID, 2019 | Attributable to smoking | Smoking | Per year | | | 100.1 | 500650* | 10.5 | 515.7 | 515.7 | | | 525.7 |
| | | Thailand | Bundhamcharoen K, 2016 | | Smoking | Per year | | | 50.5 | 6.2 | | | 354.3 | | | 418.4 |

## 3.2 COPD, national + city or region, by income level

**Table S3.2** Economic burden of COPD at national and subnational level

| Attribution | Income group | Country | Reference ID | Subgroup | Attribution (detail) | Area | Unit | Medication | Hospitalization | Direct medical costs | Direct non-medical costs | Direct costs | Indirect costs | Mortality costs | Morbidity costs | DALY costs | Total costs |
|---|---|---|---|---|---|---|---|---|---|---|---|---|---|---|---|---|---|
| Air pollution | Low income | Low income | Yin H, 2021 | Low-income countries | $PM_{2.5}$ | Regional | During 2000-2016 period | | | | | | | 17816.6 | | | |
| | Lower middle income | India | Maji KJ, 2017b | 2002 | Air pollution ($NO_2$, $SO_2$, $PM_{10}$) | Agra City, Uttar Pradesh | Per year | | 64531* | | | | | | | | |
| | | | | 2003 | Air pollution ($NO_2$, $SO_2$, $PM_{10}$) | Agra City, Uttar Pradesh | Per year | | 79430* | | | | | | | | |
| | | | | 2004 | Air pollution ($NO_2$, $SO_2$, $PM_{10}$) | Agra City, Uttar Pradesh | Per year | | 74465* | | | | | | | | |
| | | | | 2005 | Air pollution ($NO_2$, $SO_2$, $PM_{10}$) | Agra City, Uttar Pradesh | Per year | | 86893* | | | | | | | | |
| | | | | 2006 | Air pollution ($NO_2$, $SO_2$, $PM_{10}$) | Agra City, Uttar Pradesh | Per year | | 118132* | | | | | | | | |
| | | | | 2007 | Air pollution ($NO_2$, | Agra City, Uttar Pradesh | Per year | | 115830* | | | | | | | | |

| Attribution | Income group | Country | Reference ID | Subgroup | Attribution (detail) | Area | Unit | Medication | Hospitalization | Direct medical costs | Direct non-medical costs | Direct costs | Indirect costs | Mortality costs | Morbidity costs | DALY costs | Total costs |
|---|---|---|---|---|---|---|---|---|---|---|---|---|---|---|---|---|---|
| | | | | | $SO_2$, $PM_{10}$) | | | | | | | | | | | | |
| | | | | 2008 | Air pollution ($NO_2$, $SO_2$, $PM_{10}$) | Agra City, Uttar Pradesh | Per year | | 112480* | | | | | | | | |
| | | | | 2009 | Air pollution ($NO_2$, $SO_2$, $PM_{10}$) | Agra City, Uttar Pradesh | Per year | | 112867* | | | | | | | | |
| | | | | 2010 | Air pollution ($NO_2$, $SO_2$, $PM_{10}$) | Agra City, Uttar Pradesh | Per year | | 95205* | | | | | | | | |
| | | | | 2011 | Air pollution ($NO_2$, $SO_2$, $PM_{10}$) | Agra City, Uttar Pradesh | Per year | | 89220* | | | | | | | | |
| | | | | 2012 | Air pollution ($NO_2$, $SO_2$, $PM_{10}$) | Agra City, Uttar Pradesh | Per year | | 116639* | | | | | | | | |
| | | | | 2013 | Air pollution ($NO_2$, $SO_2$, $PM_{10}$) | Agra City, Uttar Pradesh | Per year | | 127883* | | | | | | | | |
| | | | | 2014 | Air pollution ($NO_2$, | Agra City, Uttar Pradesh | Per year | | 147569* | | | | | | | | |

| Attribution | Income group | Country | Reference ID | Subgroup | Attribution (detail) | Area | Unit | Medication | Hospitalization | Direct medical costs | Direct non-medical costs | Direct costs | Indirect costs | Mortality costs | Morbidity costs | DALY costs | Total costs |
|---|---|---|---|---|---|---|---|---|---|---|---|---|---|---|---|---|---|
| | | | | | $SO_2$, $PM_{10}$) | | | | | | | | | | | | |
| | | | Nair M, 2021 | 2017 | Air pollution | Thirty one non-attainment cities | Per year | | | | | | | 14659.2 | | | |
| | | Iran | Bayat R, 2019 | | $PM_{2.5}$ | Tehran | Per year | | | | | | | 25.9 | | | |
| | | | | | $PM_{2.5}$ | Tehran | Per year | | | | | | | 40.3 | | | |
| | | | | | $PM_{2.5}$ | Tehran | Per year | | | | | | | 110.9 | | | |
| | | | | | $PM_{2.5}$ | Tehran | Per year | | | | | | | 146.9 | | | |
| | | | | | $PM_{2.5}$ | Tehran | Per year | | | | | | | 211.7 | | | |
| | | | Hadei M, 2020 | | $PM_{2.5}$ | 25 major Iranian cities | Not reported | | | | | | | | | | 202.3 |
| | | Lower middle income | Yin H, 2021 | Lower middle-income countries | $PM_{2.5}$ | Regional | During 2000-2016 period | | | | | | | 1562413 | | | |
| | | Nigeria | Etchie TO, 2018 | 2005 | Ambient $PM_{2.5}$ | National | Per year | | | | | | | 802.4 | | | |
| | | | | 2015 | Ambient $PM_{2.5}$ | National | Per year | | | | | | | 717.3 | | | |
| | Upper middle income | China | Huang J, 2018 | Overall burden | Ozone | Ningbo, Yangtze River Delta | From 2011-2015 | | | | | | | 4443.1 | | | |
| | | | | Attributable to ozone | Ozone | Ningbo, Yangtze River Delta | From 2011-2015 | | | | | | | 324.4 | | | |
| | | | Lu X, 2017 | 2004 | $PM_{2.5}$ | Pearl River Delta | Per year | | | | | | | 8321.2 | | | |
| | | | | 2005 | $PM_{2.5}$ | Pearl River Delta | Per year | | | | | | | 7182.4 | | | |

| Attribution | Income group | Country | Reference ID | Subgroup | Attribution (detail) | Area | Unit | Medication | Hospitalization | Direct medical costs | Direct non-medical costs | Direct costs | Indirect costs | Mortality costs | Morbidity costs | DALY costs | Total costs |
|---|---|---|---|---|---|---|---|---|---|---|---|---|---|---|---|---|---|
| | | | | 2006 | $PM_{2.5}$ | Pearl River Delta | Per year | | | | | | | 7275.6 | | | |
| | | | | 2007 | $PM_{2.5}$ | Pearl River Delta | Per year | | | | | | | 7119.4 | | | |
| | | | | 2008 | $PM_{2.5}$ | Pearl River Delta | Per year | | | | | | | 6424.6 | | | |
| | | | | 2009 | $PM_{2.5}$ | Pearl River Delta | Per year | | | | | | | 5797 | | | |
| | | | | 2010 | $PM_{2.5}$ | Pearl River Delta | Per year | | | | | | | 6518.9 | | | |
| | | | | 2011 | $PM_{2.5}$ | Pearl River Delta | Per year | | | | | | | 5648.6 | | | |
| | | | | 2012 | $PM_{2.5}$ | Pearl River Delta | Per year | | | | | | | 6195.7 | | | |
| | | | | 2013 | $PM_{2.5}$ | Pearl River Delta | Per year | | | | | | | 5031.5 | | | |
| | | | Maji KJ, 2017c | COPD | $PM_{10}$ | National | Per year | | 515.6 | | | | | | | | |
| | | | Aunan K, 2013 | Scenario 1a: Individual households switch from No-chimney stove to Second generation improved stove. | $PM_{2.5}$ | Guizhou province | Per household | | | | | | | | | | 2317* |
| | | | | Scenario 1b: Individual households switch from Chimney | $PM_{2.5}$ | Guizhou province | Per household | | | | | | | | | | 515* |

| Attribution | Income group | Country | Reference ID | Subgroup | Attribution (detail) | Area | Unit | Medication | Hospitalization | Direct medical costs | Direct non-medical costs | Direct costs | Indirect costs | Mortality costs | Morbidity costs | DALY costs | Total costs |
|---|---|---|---|---|---|---|---|---|---|---|---|---|---|---|---|---|---|
| | | | | stove to Second generation improved stove. Scenario 2a: Community level switch from No-chimney stove to Second generation improved stove (pellets). | $PM_{2.5}$ | Guizhou province | Per household | | | | | | | | | | 4852* |
| | | | | Scenario 2b: Community level switch from Chimney stove to Second generation improved stove (pellets). | $PM_{2.5}$ | Guizhou province | Per household | | | | | | | | | | 1252* |
| | | | Huang J, 2021 | | Ambient nitrogen dioxide | Thirty seven major cities in 20 provincial administrative regions | During 2013-2017 period | | | | | | | 4271.6 | | | |
| | | | Li J, 2021 | Overall burden | Sulfur dioxide | Forty eight large cities | During 2013-2017 period | | | | | | | 156738.3 | | | |

| Attribution | Income group | Country | Reference ID | Subgroup | Attribution (detail) | Area | Unit | Medication | Hospitalization | Direct medical costs | Direct non-medical costs | Direct costs | Indirect costs | Mortality costs | Morbidity costs | DALY costs | Total costs |
|---|---|---|---|---|---|---|---|---|---|---|---|---|---|---|---|---|---|
| | | | | Attributable to sulfur | Sulfur dioxide | Forty eight large cities | During 2013-2017 period | | | | | | | 2967.8 | | | |
| | | | Zhang P, 2021 | Overall burden | Air pollution | Chengdu | During 2013-2017 period | | 44.5 | | | | | | | | |
| | | | | Tertiary hospitals | Air pollution | Chengdu | During 2013-2017 period | | 5.4 | | | | | | | | |
| | | | | Secondary hospitals | Air pollution | Chengdu | During 2013-2017 period | | 16.9 | | | | | | | | |
| | | | | Primary hospitals | Air pollution | Chengdu | During 2013-2017 period | | 15.4 | | | | | | | | |
| | | | | Age ≤65 | Air pollution | Chengdu | During 2013-2017 period | | 7.3 | | | | | | | | |
| | | | | Age ≥65–≤80 | Air pollution | Chengdu | During 2013-2017 period | | 26 | | | | | | | | |
| | | | | Age ≥80 | Air pollution | Chengdu | During 2013-2017 period | | 11.2 | | | | | | | | |
| | | Thailand | Mueller W, 2021 | | $PM_{2.5}$ | National | During 1996-2016 period | | | | | | | 6903.9 | | 565.3 | |
| | | Turkey | Kara E, 2021 | | Air pollution | Nigde | Per 1000 new cases | | | 1.7 | | | | | | | |
| | | Upper middle | Yin H, 2021 | Upper middle- | $PM_{2.5}$ | Regional | During 2000- | | | | | | | 3672934 | | | |

| Attribution | Income group | Country | Reference ID | Subgroup | Attribution (detail) | Area | Unit | Medication | Hospitalization | Direct medical costs | Direct non-medical costs | Direct costs | Indirect costs | Mortality costs | Morbidity costs | DALY costs | Total costs |
|---|---|---|---|---|---|---|---|---|---|---|---|---|---|---|---|---|---|
| | | income | | income countries | | | 2016 period | | | | | | | | | | |
| No attribution | Lower middle income | Iran | Rezaei S, 2017 | Overall burden | Not applicable | National | Per year | | 111.6 | | 236.9 | | | 40.8 | 11.1 | | |
| No attribution | Upper middle income | Brazil | da Cruz DM, 2015 | 2008 | Not applicable | São Carlos | Per year | | | 41704* | | | | | | | |
| | | | | 2009 | Not applicable | São Carlos | Per year | | | 51429* | | | | | | | |
| | | | | 2010 | Not applicable | São Carlos | Per year | | | 42821* | | | | | | | |
| | | | | 2011 | Not applicable | São Carlos | Per year | | | 38279* | | | | | | | |
| | | | | 2012 | Not applicable | São Carlos | Per year | | | 31355* | | | | | | | |
| | | | | 2008-2012 | Not applicable | São Carlos | Over 5 years | | | 41140* | | | | | | | |
| | | | Szpak R, 2022 | 2010 | Not applicable | State of Paraná | Per year | 38524* | | | | | | | | | |
| | | | | 2011 | Not applicable | State of Paraná | Per year | 151927* | | | | | | | | | |
| | | | | 2012 | Not applicable | State of Paraná | Per year | 286225* | | | | | | | | | |
| | | | | 2013 | Not applicable | State of Paraná | Per year | 480874* | | | | | | | | | |
| | | | | 2014 | Not applicable | State of Paraná | Per year | 661661* | | | | | | | | | |

| Attribution | Income group | Country | Reference ID | Subgroup | Attribution (detail) | Area | Unit | Medication | Hospitalization | Direct medical costs | Direct non-medical costs | Direct costs | Indirect costs | Mortality costs | Morbidity costs | DALY costs | Total costs |
|---|---|---|---|---|---|---|---|---|---|---|---|---|---|---|---|---|---|
| | | | | 2015 | Not applicable | State of Paraná | Per year | 682483* | | | | | | | | | |
| | | | | 2016 | Not applicable | State of Paraná | Per year | 786037* | | | | | | | | | |
| | | Bulgaria | Tachkov K, 2019 | 2013 | Not applicable | National | Per year | | 3.5 | | | | | | | | |
| | | | | 2016 | Not applicable | National | Per year | | 3.9 | | | | | | | | |
| | | | Tachkov K, 2018 | Smokers (among the cohort population) | Not applicable | 19 regions | Over 10 years | | | | | | | | | | 1 |
| | | | | Non-smokers or ex-smokers (among the cohort population) | Not applicable | 19 regions | Over 10 years | | | | | | | | | | 1.3 |
| | | | | Smokers (the entire Bulgarian population) | Not applicable | 19 regions | Over 10 years | | | | | | | | | | 762.1 |
| | | | | Non-smokers or ex-smokers (the entire Bulgarian population) | Not applicable | 19 regions | Over 10 years | | | | | | | | | | 404.1 |
| | | China | Chen X, 2016 | | Not applicable | Urban centres of Beijing, Guangzhou, Shanghai and Chengdu | Per year | | | 37191.8 | 1668.6 | | 6480.5 | | | | |
| | | | Yu XQ, 2019 | Western medicine group | Not applicable | Not reported | Per 100,000 | | | | | | | | | | 2404.9 |

| Attribution | Income group | Country | Reference ID | Subgroup | Attribution (detail) | Area | Unit | Medication | Hospitalization | Direct medical costs | Direct non-medical costs | Direct costs | Indirect costs | Mortality costs | Morbidity costs | DALY costs | Total costs |
|---|---|---|---|---|---|---|---|---|---|---|---|---|---|---|---|---|---|
| | | | | | | | patients in 40 years | | | | | | | | | | |
| | | | | Traditional Chinese medicine group | Not applicable | Not reported | Per 100,000 patients in 40 years | | | | | | | | | | 2137.8 |
| | | | | Combined group | Not applicable | Not reported | Per 100,000 patients in 40 years | | | | | | | | | | 2595.1 |
| | | | Salem A, 2021 | Current scenario, representative of the current treatment paradigm, in which only some (i.e., 38.50%) of the patients with COPD are treated with long-acting maintenance therapy after hospital charge | Not applicable | National | Per year | | | 3040.9 | | | | | | | |
| | | | | Future (hypothetical) scenario in which all patients with COPD are treated | Not applicable | National | Per year | | | 2445.5 | | | | | | | |

| Attribution | Income group | Country | Reference ID | Subgroup | Attribution (detail) | Area | Unit | Medication | Hospitalization | Direct medical costs | Direct non-medical costs | Direct costs | Indirect costs | Mortality costs | Morbidity costs | DALY costs | Total costs |
|---|---|---|---|---|---|---|---|---|---|---|---|---|---|---|---|---|---|
| | | | | with long-acting maintenance therapy after hospitalisation due to a severe exacerbation | | | | | | | | | | | | | |
| | | | Cai L, 2014 | The whole study population | Not applicable | Four rural areas of Yunnan Province | Per year | | | | | 32.1 | 390010* | | | | 32.5 |
| | | | | Males | Not applicable | Four rural areas of Yunnan Province | Per year | | | | | 22.8 | 226457* | | | | 23 |
| | | | | Females | Not applicable | Four rural areas of Yunnan Province | Per year | | | | | 855505* | 12581* | | | | 880667* |
| | | | Luo L, 2020 | 2012 | Not applicable | A large city in western China | Per year | | 146.3 | | | | | | | | |
| | | | | 2013 | Not applicable | A large city in western China | Per year | | 198.5 | | | | | | | | |
| | | Paraguay | Bardach A, 2018 | Overall burden | Not applicable | National | Per year | | | 108.1 | | | | | | | |
| | | Peru | Bardach AE, 2016 | Overall burden | Not applicable | National | Per year | | | | | 294.3 | | | | | |
| | | Russia | Artyukhov IP, 2015 | | Not applicable | Krasnoyarsk region | Per year | | | | | | | | | | 7.3 |
| | | | Gaygolnik, 2016 | 2009 | Not applicable | Krasnoyarsk city | Per year | 832334* | | | | | | | | | |

| Attribution | Income group | Country | Reference ID | Subgroup | Attribution (detail) | Area | Unit | Medication | Hospitalization | Direct medical costs | Direct non-medical costs | Direct costs | Indirect costs | Mortality costs | Morbidity costs | DALY costs | Total costs |
|---|---|---|---|---|---|---|---|---|---|---|---|---|---|---|---|---|---|
| | | | | 2010 | Not applicable | Krasnoyarsk city | Per year | 892438* | | | | | | | | | |
| | | | | 2011 | Not applicable | Krasnoyarsk city | Per year | 879831* | | | | | | | | | |
| | | | | 2012 | Not applicable | Krasnoyarsk city | Per year | 808786* | | | | | | | | | |
| | | | | 2013 | Not applicable | Krasnoyarsk city | Per year | 869685* | | | | | | | | | |
| | | | | 2014 | Not applicable | Krasnoyarsk city | Per year | 1 | | | | | | | | | |
| | | | Kontsevaia AV, 2019 | | Not applicable | National | Per year | | | 97.7 | 5.7 | 103.4 | 2739.1 | 2668.8 | | | 2842.5 |
| | | | Nedogoda SV, 2020 | With the original treatment distribution over one-year horizon | Not applicable | 14 major cities of Russia | Per year | 2.5 | | | | | | | | | |
| | | | | Transferring to tiotropium bromide and olodaterol fixed combination treatment over one-year horizon | Not applicable | 14 major cities of Russia | Per year | 2.2 | | | | | | | | | |
| | | | | With the original treatment distribution over three- | Not applicable | 14 major cities of Russia | Over three years | 6.2 | | | | | | | | | |

| Attribution | Income group | Country | Reference ID | Subgroup | Attribution (detail) | Area | Unit | Medication | Hospitalization | Direct medical costs | Direct non-medical costs | Direct costs | Indirect costs | Mortality costs | Morbidity costs | DALY costs | Total costs |
|---|---|---|---|---|---|---|---|---|---|---|---|---|---|---|---|---|---|
| | | | | year horizon Transferring to tiotropium bromide and olodaterol fixed combination treatment over three-year horizon | Not applicable | 14 major cities of Russia | Over three years | 5.4 | | | | | | | | | |
| | | | Orlova EA, 2021 | 2015 | Not applicable | Astrakhan region | Per year | | 299037* | 443553* | 76456* | 520188* | 1.8 | 1.8 | | | 2.4 |
| | | | | 2016 | Not applicable | Astrakhan region | Per year | | 344948* | 469610* | 50899* | 520509* | 2.6 | 2.6 | | | 2.6 |
| | | | | 2017 | Not applicable | Astrakhan region | Per year | | 369713* | 476426* | 87398* | 563825* | 1.3 | 1.3 | | | 1.9 |
| | | | | 2018 | Not applicable | Astrakhan region | Per year | | 392537* | 515352* | 76192* | 591544* | 1.9 | 1.9 | | | 2.4 |
| | | | | 2019 | Not applicable | Astrakhan region | Per year | | 482517* | 604868* | 67091* | 671959* | 2* | 2* | | | 2.9 |
| | | | Sapunova ID, 2019 | Overall burden | Not applicable | National | Per year | | | | | 103.5 | 2705.2 | 2705.2 | | | 2808.6 |
| | | | Zyryanov SK, 2018 | Single bronchodilator use, 1st year | Not applicable | National | Per year | | | 2.2 | | | | | | | |
| | | | | Single bronchodilator use, 2nd year | Not applicable | National | Per year | | | 2.2 | | | | | | | |
| | | | | Single bronchodila | Not applicable | National | Per year | | | 2.1 | | | | | | | |

| Attribution | Income group | Country | Reference ID | Subgroup | Attribution (detail) | Area | Unit | Medication | Hospitalization | Direct medical costs | Direct non-medical costs | Direct costs | Indirect costs | Mortality costs | Morbidity costs | DALY costs | Total costs |
|---|---|---|---|---|---|---|---|---|---|---|---|---|---|---|---|---|---|
| | | | | tor use, 3rd year | | | | | | | | | | | | | |
| | | | | Dual bronchodilators (glycopyrronium bromide + indacaterol) use, 1st year | Not applicable | National | Per year | | | 1.8 | | | | | | | |
| | | | | Dual bronchodilators (glycopyrronium bromide + indacaterol) use, 2nd year | Not applicable | National | Per year | | | 1.7 | | | | | | | |
| | | | | Dual bronchodilators (glycopyrronium bromide + indacaterol) use, 3rd year | Not applicable | National | Per year | | | 1.7 | | | | | | | |
| | | | Ortaköylü MG, 2016 | | Not applicable | Istanbul | Per year | | | 842272* | | | | | | | |
| | | Turkey | Ozdemir T, 2021 | 2012 | Not applicable | National | Per year | | | 274.6 | | | | | | | |
| | | | | 2013 | Not applicable | National | Per year | | | 274.2 | | | | | | | |
| | | | | 2014 | Not applicable | National | Per year | | | 284.8 | | | | | | | |
| | | | | 2015 | Not applicable | National | Per year | | | 278.4 | | | | | | | |

| Attribution | Income group | Country | Reference ID | Subgroup | Attribution (detail) | Area | Unit | Medication | Hospitalization | Direct medical costs | Direct non-medical costs | Direct costs | Indirect costs | Mortality costs | Morbidity costs | DALY costs | Total costs |
|---|---|---|---|---|---|---|---|---|---|---|---|---|---|---|---|---|---|
| | | | | 2016 | Not applicable | National | Per year | | | 285.4 | | | | | | | |
| Not applicable | | Thailand | Patanavanich R, 2018 | COPD | Not applicable | National | Over period from 2007 to 2014 | | 1404.3 | | | | | | | | |
| | | | | COPD with TB diagnosis | Not applicable | National | Over period from 2007 to 2014 | | 31.8 | | | | | | | | |
| Second hand smoke | | China | Cai L, 2014 | Burden attributable to second hand smoke among the whole study population | Second hand smoke | Four rural areas of Yunnan Province | Per year | | | | | 98.4 | 1.4 | | | | 99.8 |
| | | | | Burden attributable to second hand smoke among males | Second hand smoke | Four rural areas of Yunnan Province | Per year | | | | | 63.1 | 553562* | | | | 63.7 |
| | | | | Burden attributable to second hand smoke among females | Second hand smoke | Four rural areas of Yunnan Province | Per year | | | | | 35.3 | 868086* | | | | 36.2 |
| Smoking | Lower middle | Bolivia | Pichon-Riviere A, 2016 | Bolivia | Smoking | National | Per year | | | 39.4 | | | | | | | |
| | | | | Bolivia | Smoking | National | Per year | | | 39.8 | | | | | | | |

| Attribution | Income group | Country | Reference ID | Subgroup | Attribution (detail) | Area | Unit | Medication | Hospitalization | Direct medical costs | Direct non-medical costs | Direct costs | Indirect costs | Mortality costs | Morbidity costs | DALY costs | Total costs |
|---|---|---|---|---|---|---|---|---|---|---|---|---|---|---|---|---|---|
| | income | Honduras | Pichon-Riviere A, 2020 | Honduras | Smoking | National | Per year | | | 15.5 | | | | | | | |
| | | India | Wu DC, 2020 | | Smoking | Four states: Karnataka, Assam, Uttar Pradesh, and Maharashtra | Per year | | | 27.7 | | | | | | | |
| | | Indonesia | Kristina SA, 2018 | COPD, male | Smoking | National | Per year | | | 789.6 | | | | | | | |
| | | | | COPD, female | Smoking | National | Per year | | | 2.1 | | | | | | | |
| | | Iran | Rezaei S, 2017 | Smoking-attributable | Smoking | National | Per year | | 18 | | 37.8 | | | 145 | 11.1 | | 228.7 |
| | | | Ghobadi M, 2017 | Age 35-59 | Smoking | Kerman | Per year | | | | | 989319* | 1.5 | | | | |
| | | | | Age >60 | Smoking | Kerman | Per year | | | | | 1.6 | 3.5 | | | | |
| | | | Varmarghani M, 2021 | | Smoking | Mashhad | Per 100,000 patients per year | 800921* | | 2 | | | | | | | |
| | | Vietnam | Anh PTH, 2016 | | Smoking | National | Per year | | | | | 401.9 | 105.8 | 102.4 | 345818* | | 507.7 |
| | Upper middle income | Brazil | Pichon-Riviere A, 2016 | Brazil | Smoking | National | Per year | | | 3035.1 | | | | | | | |
| | | | Pichon-Riviere A, 2020 | Brazil | Smoking | National | Per year | | | 4004.2 | | | | | | | |
| | | China | Cai L, 2014 | Burden attributable to smoking among the whole study population | Smoking | Four rural areas of Yunnan Province | Per year | | | | | 118.8 | 1.1 | | | | 120.2 |

| Attribution | Income group | Country | Reference ID | Subgroup | Attribution (detail) | Area | Unit | Medication | Hospitalization | Direct medical costs | Direct non-medical costs | Direct costs | Indirect costs | Mortality costs | Morbidity costs | DALY costs | Total costs |
|---|---|---|---|---|---|---|---|---|---|---|---|---|---|---|---|---|---|
| | | | | Burden attributable to smoking among males | Smoking | Four rural areas of Yunnan Province | Per year | | | | | 116.6 | 1 | | | | 117.6 |
| | | | | Burden attributable to smoking among females | Smoking | Four rural areas of Yunnan Province | Per year | | | | | 2.5 | 62905* | | | | 2.6 |
| | | Colombia | Pichon-Riviere A, 2016 | Columbia | Smoking | National | Per year | | | 376.5 | | | | | | | |
| | | | Pichon-Riviere A, 2020 | Colombia | Smoking | National | Per year | | | 377.4 | | | | | | | |
| | | Costa Rica | | Costa Rica | Smoking | National | Per year | | | 37.6 | | | | | | | |
| | | Ecuador | | Ecuador | Smoking | National | Per year | | | 210.1 | | | | | | | |
| | | Mexico | Pichon-Riviere A, 2016 | Mexico | Smoking | National | Per year | | | 1639.2 | | | | | | | |
| | | | Pichon-Riviere A, 2020 | Mexico | Smoking | National | Per year | | | 1638.2 | | | | | | | |
| | | Paraguay | Bardach A, 2018 | Attributable to smoking | Smoking | National | Per year | | | 79.9 | | | | | | | |
| | | | Pichon-Riviere A, 2020 | Paraguay | Smoking | National | Per year | | | 79.8 | | | | | | | |
| | | Peru | Pichon-Riviere A, 2016 | Peru | Smoking | National | Per year | | | 226.2 | | | | | | | |
| | | | Bardach AE, 2016 | Attributable to smoking | Smoking | National | Per year | | | | | 226.1 | | | | | |
| | | | Pichon-Riviere A, 2020 | Peru | Smoking | National | Per year | | | 227 | | | | | | | |
| | | Russia | Artyukhov IP, 2016 | Male | Smoking | Krasnoyarsk region | Per year | | | | | | | | | | 12.5 |
| | | | | Female | Smoking | Krasnoyarsk region | Per year | | | | | | | | | | 4.5 |

| Attribution | Income group | Country | Reference ID | Subgroup | Attribution (detail) | Area | Unit | Medication | Hospitalization | Direct medical costs | Direct non-medical costs | Direct costs | Indirect costs | Mortality costs | Morbidity costs | DALY costs | Total costs |
|---|---|---|---|---|---|---|---|---|---|---|---|---|---|---|---|---|---|
| | | | Yagudra, 2018 | 2009 | Smoking | National | Per year | | | 501.2 | | | | 378.6 | | | |
| | | | | 2009-2016 | Smoking | National | Per year | | | 2329.2 | | | | 106 | | | |
| | | | Sapunova ID, 2019 | Attributable to smoking | Smoking | National | Per year | | | 100.1 | 500650* | 10.5 | 515.7 | 515.7 | | | 525.7 |
| | | Thailand | Bundhamcharoen K, 2016 | | Smoking | National | Per year | | | 50.5 | 6.2 | | | 354.3 | | | 418.4 |

## 3.3 Chronic bronchitis, national + city or region, by income level

**Table S3.3** Economic burden of chronic bronchitis at national and subnational level

| Attribution | Income group | Country | Reference ID | Subgroup | Attribution (detail) | Area | Unit | Medication | Hospitalization | Direct medical costs | Direct non-medical costs | Direct costs | Indirect costs | Mortality costs | Morbidity costs | DALY costs | Total costs |
|---|---|---|---|---|---|---|---|---|---|---|---|---|---|---|---|---|---|
| Air pollution | Lower middle income | India | Maji KJ, 2017a | Mumbai, 1995 | PM$_{10}$ pollution | Mumbai | Per year | | | | | | | | | 933 | |
| | | | | Mumbai, 2000 | PM$_{10}$ pollution | Mumbai | Per year | | | | | | 11.2 | | | 1004.4 | |
| | | | | Mumbai, 2005 | PM$_{10}$ pollution | Mumbai | Per year | | | | | | 410.8 | | | 965.4 | |
| | | | | Mumbai, 2010 | PM$_{10}$ pollution | Mumbai | Per year | | | | | | | | | 1219.3 | |
| | | | | Mumbai, 2015 | PM$_{10}$ pollution | Mumbai | Per year | | | | | | | | | 1277.5 | |
| | | | | Delhi, 1995 | PM$_{10}$ pollution | Delhi | Per year | | | | | | | | | 949 | |
| | | | | Delhi, 2000 | PM$_{10}$ pollution | Delhi | Per year | | | | | | | | | 1081.7 | |
| | | | | Delhi, 2005 | PM$_{10}$ pollution | Delhi | Per year | | | | | | | | | 1255.6 | |
| | | | | Delhi, 2010 | PM$_{10}$ pollution | Delhi | Per year | | | | | | | | | 1613.8 | |
| | | | | Delhi, 2015 | PM$_{10}$ pollution | Delhi | Per year | | | | | | | | | 1923.3 | |
| | | Iran | Fard RF, 2016 | PM$_{10}$ | PM$_{10}$ | Qom province | Per 2000 cases or | | | | | | | | 64374* | | |

| Attribution | Income group | Country | Reference ID | Subgroup | Attribution (detail) | Area | Unit | Medication | Hospitalization | Direct medical costs | Direct non-medical costs | Direct costs | Indirect costs | Mortality costs | Morbidity costs | DALY costs | Total costs |
|---|---|---|---|---|---|---|---|---|---|---|---|---|---|---|---|---|---|
| | | | | | | | years of life lost/year | | | | | | | | | | |
| | | | | Nitrate | Nitrate | Qom province | Per 2000 cases or years of life lost/year | | | | | | | | 1.6 | | |
| | Upper middle income | Bulgaria | Tarín-Carrasco P, 2019 | Present climatology scenario (1996–2015), Bulgaria | Air pollution | Not reported | Per year | | | | | | | 36837* | | | |
| | | | | Future climate scenario (2071–2100, RCP8.5), Bulgaria | Air pollution | Not reported | Per year | | | | | | | 37189* | | | |
| | | China | Zhu B, 2019 | 2013 | $PM_{2.5}$ | National | Per year | | | | | | | 1.3 | | | |
| | | | | 2014 | $PM_{2.5}$ | National | Per year | | | | | | | 1.2 | | | |
| | | | Maji KJ, 2017c | Chronic bronchits | $PM_{10}$ | National | Per year | | | | | | | 28064.3 | | | |
| | | | Maji KJ, 2018 | Chronic bronchits | $PM_{2.5}$ | 338 cities in 31 provinces | Per year | | 5812.2 | | | | | | | | |
| | | | Yin H, 2015 | 2008 | $PM_{10}$ | Beijing | Per year | | | | | | | | | | 14019.9 |
| | | | | 2009 | $PM_{10}$ | Beijing | Per year | | | | | | | | | | 14563.1 |
| | | | | 2010 | $PM_{10}$ | Beijing | Per year | | | | | | | | | | 16840.6 |

| Attribution | Income group | Country | Reference ID | Subgroup | Attribution (detail) | Area | Unit | Medication | Hospitalization | Direct medical costs | Direct non-medical costs | Direct costs | Indirect costs | Mortality costs | Morbidity costs | DALY costs | Total costs |
|---|---|---|---|---|---|---|---|---|---|---|---|---|---|---|---|---|---|
| | | | | 2011 | $PM_{10}$ | Beijing | Per year | | | | | | | | | | 15718 |
| | | | | 2012 | $PM_{10}$ | Beijing | Per year | | | | | | | | | | 15197 |
| | | | Liao Q, 2020 | 2015 | $PM_{2.5}$ | Gansu Province | Per year | | | | | | | 4551.6 | | | |
| | | | | 2016 | $PM_{2.5}$ | Gansu Province | Per year | | | | | | | 4633.1 | | | |
| | | | | 2017 | $PM_{2.5}$ | Gansu Province | Per year | | | | | | | 4564.2 | | | |
| | | | Wei GR, 2018 | 2014, age 0-14 | $PM_{2.5}$ | Xi'an | Per year | | | | | | | | | | 2391.6 |
| | | | | 2014, age ≥15 | $PM_{2.5}$ | Xi'an | Per year | | | | | | | | | | 2453.9 |
| | | | | 2015, age 0-14 | $PM_{2.5}$ | Xi'an | Per year | | | | | | | | | | 1526.5 |
| | | | | 2015, age ≥15 | $PM_{2.5}$ | Xi'an | Per year | | | | | | | | | | 1701.2 |
| | | | | 2016, age 0-14 | $PM_{2.5}$ | Xi'an | Per year | | | | | | | | | | 2604.3 |
| | | | | 2016, age ≥15 | $PM_{2.5}$ | Xi'an | Per year | | | | | | | | | | 2689.5 |
| | | | Xu X, 2021 | | $PM_{2.5}$ | Ji'nan | Per year | | | | | | | 24.3 | | | |
| | | Romania | Tarín-Carrasco P, 2019 | Present climatology scenario (1996–2015), Romania | Air pollution | Not reported | Per year | | | | | | | 81525* | | | |
| | | | | Future climate scenario (2071–2100, RCP8.5), Romania | Air pollution | Not reported | Per year | | | | | | | 83001* | | | |

## 3.4 Other diseases, national + city or region, by income level

**Table S3.4** Economic burden of other diseases at national and subnational level

| Attribution | Income group | Country | Reference ID | Subgroup | Attribution (detail) | Area | Disease | Unit | Medication | Hospitalization | Direct medical costs | Direct non-medical costs | Direct costs | Indirect costs | Mortality costs | Morbidity costs | DALY costs | Total costs |
|---|---|---|---|---|---|---|---|---|---|---|---|---|---|---|---|---|---|---|
| Air pollution | Upper middle income | China | Wang Z, 2021 | 2013 | Air pollution | Guangdong | AECOPD | Per year | | 15.8 | | | | | | | | |
| | | | | 2014 | Air pollution | Guangdong | AECOPD | Per year | | 13.9 | | | | | | | | |
| | | | | 2015 | Air pollution | Guangdong | AECOPD | Per year | | 10.6 | | | | | | | | |
| | | | | 2016 | Air pollution | Guangdong | AECOPD | Per year | | 10.2 | | | | | | | | |
| | | | | 2017 | Air pollution | Guangdong | AECOPD | Per year | | 11.8 | | | | | | | | |
| COPD associated malnutrition | | | Linthicum MT, 2015 | Overall population (age) | COPD associated malnutrition | National | COPD associated malnutrition | Per year | | | | | | | | | | 17588.6 |
| | | | | 0-14 years (age) | COPD associated malnutrition | National | COPD associated malnutrition | Per year | | | | | | | | | | 6.1 |
| | | | | 15-59 years (age) | COPD associated malnutrition | National | COPD associated malnutrition | Per year | | | | | | | | | | 4540.6 |
| | | | | 60+ years (age) | COPD associated malnutrition | National | COPD associated malnutrition | Per year | | | | | | | | | | 13041.9 |
| No attribution | Lower middle | Mongolia | Dugee O, 2017 | | Not applicable | National | COPD/asthma | Per year | 27857* | 2.2 | 3.1 | | | | | | | |

| Attribution | Income group | Country | Reference ID | Subgroup | Attribution (detail) | Area | Disease | Unit | Medication | Hospitalization | Direct medical costs | Direct non-medical costs | Direct costs | Indirect costs | Mortality costs | Morbidity costs | DALY costs | Total costs |
|---|---|---|---|---|---|---|---|---|---|---|---|---|---|---|---|---|---|---|
| | income | | | | | | | | | | | | | | | | | |
| Not applicable | Upper middle income | Thailand | Patanavanich R, 2018 | Bronchitis emphysema | Not applicable | National | Bronchitis and emphysema | Over period from 2007 to 2014 | | 33 | | | | | | | | |
| | | | | Bronchitis emphysema with TB diagnosis | Not applicable | National | Bronchitis and emphysema | Over period from 2007 to 2014 | | 13.6 | | | | | | | | |
| Smoking | Lower middle income | Indonesia | Kristina SA, 2018 | Bronchitis, male | Smoking | National | Bronchitis and emphysema | Per year | | | 9.7 | | | | | | | |
| | | | | Bronchitis, female | Smoking | National | Bronchitis and emphysema | Per year | | | 16152* | | | | | | | |

## 3-II Table of disease burden

DALY denotes disability-adjusted life year.

## 3.5 COPD, national, by income level

**Table S3.5** Disease burden of COPD at national level

| Attribution | Income group | Country | Reference ID | Subgroup | Attribution (detail) | Unit | Mortality | Morbidity (DALY) | Years of life lost |
|---|---|---|---|---|---|---|---|---|---|
| Air pollution | Upper middle income | China | Maji KJ, 2017c | COPD | $PM_{10}$ | Per year | | 5,061 | |
| Air pollution | Upper middle income | China | Maji KJ, 2018 | COPD | $PM_{2.5}$ | Per year | 105,000 | | |
| Air pollution | Upper middle income | Thailand | Mueller W, 2021 | | $PM_{2.5}$ | During 1996-2016 period | 4,999 | 31,090 | |
| No attribution | Lower middle income | Iran | Rezaei S, 2017 | Overall burden | Not applicable | Per year | 8,286 | | 10,191,325 |
| No attribution | Upper middle income | Argentina | Alcaraz A, 2016 | Overall burden | Not applicable | Per year | 11,856 | | |
| No attribution | Upper middle income | China | Salem A, 2021 | Current scenario, representativeof the current treatment paradigm, in which only some (i.e., 38.50%) of the patients with COPD are treated with long-acting maintenance therapy after hospital charge | Not applicable | Per year | 507,705 | | |
| No attribution | Upper middle income | China | Salem A, 2021 | Future (hypothetical) scenario in which all patients with COPD are treated with long-acting maintenance therapy after hospitalisation due to a severe exacerbation | Not applicable | Per year | 503,672 | | |
| No attribution | Upper middle income | Paraguay | Bardach A, 2018 | Overall burden | Not applicable | Per year | 1,174 | | |
| No attribution | Upper middle income | Peru | Bardach AE, 2016 | Overall burden | Not applicable | Per year | 5,074 | | |
| No attribution | Upper middle income | Russia | Kontsevaia AV, 2019 | | Not applicable | Per year | 14,706 | | 134,383 |
| Smoking | Lower middle income | Bolivia | Pichon-Riviere A, 2020 | Bolivia | Smoking | Per year | 932 | | |
| Smoking | Lower middle income | Honduras | Pichon-Riviere A, 2020 | Honduras | Smoking | Per year | 461 | | |
| Smoking | Lower middle income | Iran | Rezaei S, 2017 | Smoking-attributable | Smoking | Per year | 2,704 | | 2,216,788 |
| Smoking | | Argentina | Alcaraz A, 2016 | Attributable to smoking | Smoking | Per year | 8,846 | | |
| Smoking | | Argentina | | Argentina | Smoking | Per year | 9,259 | | |

| Attribution | Income group | Country | Reference ID | Subgroup | Attribution (detail) | Unit | Mortality | Morbidity (DALY) | Years of life lost |
|---|---|---|---|---|---|---|---|---|---|
| | Upper middle income | Brazil | Pichon-Riviere A, 2020 | Brazil | Smoking | Per year | 31,120 | | |
| | | Colombia | | Colombia | Smoking | Per year | 8,028 | | |
| | | Costa Rica | | Costa Rica | Smoking | Per year | 432 | | |
| | | Ecuador | | Ecuador | Smoking | Per year | 2,467 | | |
| | | Mexico | | Mexico | Smoking | Per year | 12,635 | | |
| | | Paraguay | Bardach A, 2018 | Attributable to smoking | Smoking | Per year | 907 | | |
| | | | Pichon-Riviere A, 2020 | Paraguay | Smoking | Per year | 907 | | |
| | | Peru | Bardach AE, 2016 | Attributable to smoking | Smoking | Per year | 3,936 | | |
| | | | Pichon-Riviere A, 2020 | Peru | Smoking | Per year | 3,933 | | |
| | | Russia | Yagudra, 2018 | 2009-2016 | Smoking | Per year | 10,831 | | |

## 3.6 COPD, national + city and region, by income level

**Table S3.6** Disease burden of COPD at national and subnational level

| Attribution | Income group | Country | Area | Reference ID | Subgroup | Attribution (detail) | Unit | Mortality | Morbidity (DALY) | Years of life lost |
|---|---|---|---|---|---|---|---|---|---|---|
| Air pollution | Low income | Low income | Regional | Yin H, 2021 | Low-income countries | $PM_{2.5}$ | During 2000-2016 period | 223,672 | | 2,758,198 |
| | Lower middle income | India | Thirty one non-attainment cities | Nair M, 2021 | 2017 | Air pollution | Per year | 1,369 | | |
| | | Iran | Tehran | Bayat R, 2019 | | $PM_{2.5}$ | Per year | 44 | | |
| | | | Tehran | | | $PM_{2.5}$ | Per year | 70 | | |
| | | | Tehran | | | $PM_{2.5}$ | Per year | 190 | | |
| | | | Tehran | | | $PM_{2.5}$ | Per year | 253 | | |
| | | | Tehran | | | $PM_{2.5}$ | Per year | 364 | | |
| | | | 25 major Iranian cities | Hadei M, 2020 | | $PM_{2.5}$ | Not reported | 274 | | |
| | | Lower middle income | Regional | Yin H, 2021 | Lower middle-income countries | $PM_{2.5}$ | During 2000-2016 period | 4,806,734 | | 54,948,644 |
| | Upper middle income | China | Ningbo, Yangtze River Delta | Huang J, 2018 | Overall burden | Ozone | From 2011-2015 | 18,472 | | 42,001 |
| | | | Ningbo, Yangtze River Delta | | Attributable to ozone | Ozone | From 2011-2015 | 18,472 | | 17,671 |
| | | | Pearl River Delta | Lu X, 2017 | 2004 | $PM_{2.5}$ | Per year | 4,770 | | |
| | | | Pearl River Delta | | 2005 | $PM_{2.5}$ | Per year | 4,260 | | |

| Attribution | Income group | Country | Area | Reference ID | Subgroup | Attribution (detail) | Unit | Mortality | Morbidity (DALY) | Years of life lost |
|---|---|---|---|---|---|---|---|---|---|---|
| | | | Pearl River Delta | | 2006 | $PM_{2.5}$ | Per year | 4,470 | | |
| | | | Pearl River Delta | | 2007 | $PM_{2.5}$ | Per year | 4,890 | | |
| | | | Pearl River Delta | | 2008 | $PM_{2.5}$ | Per year | 5,080 | | |
| | | | Pearl River Delta | | 2009 | $PM_{2.5}$ | Per year | 4,570 | | |
| | | | Pearl River Delta | | 2010 | $PM_{2.5}$ | Per year | 5,260 | | |
| | | | Pearl River Delta | | 2011 | $PM_{2.5}$ | Per year | 5,160 | | |
| | | | Pearl River Delta | | 2012 | $PM_{2.5}$ | Per year | 5,890 | | |
| | | | Pearl River Delta | | 2013 | $PM_{2.5}$ | Per year | 5,060 | | |
| | | | National | Maji KJ, 2017c | COPD | $PM_{10}$ | Per year | | 5,061 | |
| | | | 338 cities in 31 provinces | Maji KJ, 2018 | COPD | $PM_{2.5}$ | Per year | 105,000 | | |
| | | | Thirty seven major cities in 20 provincial administrative regions | Huang J, 2021 | | Ambient nitrogen dioxide | During 2013-2017 period | | | 212,128 |
| | | | Forty eight large cities | Li J, 2021 | Attributable to sulfur | Sulfur dioxide | During 2013-2017 period | | | 147,381 |
| | | Thailand | National | Mueller W, 2021 | | $PM_{2.5}$ | During 1996-2016 period | 4,999 | 31,090 | |
| | | Upper middle income | Regional | Yin H, 2021 | Upper middle-income countries | $PM_{2.5}$ | During 2000-2016 period | 4,844,413 | | 49,621,601 |
| No attribution | Lower middle income | Iran | National | Rezaei S, 2017 | Overall burden | Not applicable | Per year | 8,286 | | 10,191,325 |
| | Upper middle income | Argentina | National | Alcaraz A, 2016 | Overall burden | Not applicable | Per year | 11,856 | | |
| | | Brazil | São Carlos | da Cruz DM, 2015 | 2008 | Not applicable | Per year | 11 | | |
| | | | São Carlos | | 2009 | Not applicable | Per year | 10 | | |
| | | | São Carlos | | 2010 | Not applicable | Per year | 7 | | |
| | | | São Carlos | | 2011 | Not applicable | Per year | 13 | | |
| | | | São Carlos | | 2012 | Not applicable | Per year | 8 | | |
| | | | São Carlos | | 2008-2012 | Not applicable | Over 5 years | 40 | | |

| Attribution | Income group | Country | Area | Reference ID | Subgroup | Attribution (detail) | Unit | Mortality | Morbidity (DALY) | Years of life lost |
|---|---|---|---|---|---|---|---|---|---|---|
| | | China | National | Salem A, 2021 | Current scenario, representativeof the current treatment paradigm, in which only some (i.e., 38.50%) of the patients with COPD are treated with long-acting maintenance therapy after hospital charge | Not applicable | Per year | 507,705 | | |
| | | | National | | Future (hypothetical) scenario in which all patients with COPD are treated with long-acting maintenance therapy after hospitalisation due to a severe exacerbation | Not applicable | Per year | 503,672 | | |
| | | Paraguay | National | Bardach A, 2018 | Overall burden | Not applicable | Per year | 1,174 | | |
| | | Peru | National | Bardach AE, 2016 | Overall burden | Not applicable | Per year | 5,074 | | |
| | | Russia | Krasnoyarsk region | Artyukhov IP, 2015 | | Not applicable | Per year | | | 780 |
| | | | National | Kontsevaia AV, 2019 | | Not applicable | Per year | 14,706 | | 134,383 |
| | | | Astrakhan region | Orlova EA, 2021 | 2015 | Not applicable | Per year | 58 | | |
| | | | Astrakhan region | | 2016 | Not applicable | Per year | 56 | | |
| | | | Astrakhan region | | 2017 | Not applicable | Per year | 54 | | |
| | | | Astrakhan region | | 2018 | Not applicable | Per year | 63 | | |
| | | | Astrakhan region | | 2019 | Not applicable | Per year | 73 | | |
| Smoking | Low and middle income | Middle East & North Africa | Regional | Nagi M, 2021 | | Smoking | Per year | 49,252 | | 624,015 |
| | Lower middle income | Bolivia | National | Pichon-Riviere A, 2020 | Bolivia | Smoking | Per year | 932 | | |
| | | Honduras | National | | Honduras | Smoking | Per year | 461 | | |

| Attribution | Income group | Country | Area | Reference ID | Subgroup | Attribution (detail) | Unit | Mortality | Morbidity (DALY) | Years of life lost |
|---|---|---|---|---|---|---|---|---|---|---|
| | | India | Four states: Karnataka, Assam, Uttar Pradesh, and Maharashtra | Wu DC, 2020 | | Smoking | Per year | 152,870 | | |
| | | Iran | National | Rezaei S, 2017 | Smoking-attributable | Smoking | Per year | 2,704 | | 2,216,788 |
| | | Iran | Kerman | Ghobadi M, 2017 | Age 35-59 | Smoking | Per year | 26,442 | | 88,518 |
| | | Iran | Kerman | Ghobadi M, 2017 | Age >60 | Smoking | Per year | 178,012 | | 384,744 |
| | Upper middle income | Argentina | National | Alcaraz A, 2016 | Attributable to smoking | Smoking | Per year | 8,846 | | |
| | Upper middle income | Argentina | National | Pichon-Riviere A, 2020 | Argentina | Smoking | Per year | 9,259 | | |
| | Upper middle income | Brazil | National | Pichon-Riviere A, 2020 | Brazil | Smoking | Per year | 31,120 | | |
| | Upper middle income | Colombia | National | Pichon-Riviere A, 2020 | Colombia | Smoking | Per year | 8,028 | | |
| | Upper middle income | Costa Rica | National | Pichon-Riviere A, 2020 | Costa Rica | Smoking | Per year | 432 | | |
| | Upper middle income | Ecuador | National | Pichon-Riviere A, 2020 | Ecuador | Smoking | Per year | 2,467 | | |
| | Upper middle income | Mexico | National | Pichon-Riviere A, 2020 | Mexico | Smoking | Per year | 12,635 | | |
| | Upper middle income | Paraguay | National | Bardach A, 2018 | Attributable to smoking | Smoking | Per year | 907 | | |
| | Upper middle income | Paraguay | National | Pichon-Riviere A, 2020 | Paraguay | Smoking | Per year | 907 | | |
| | Upper middle income | Peru | National | Bardach AE, 2016 | Attributable to smoking | Smoking | Per year | 3,936 | | |
| | Upper middle income | Peru | National | Pichon-Riviere A, 2020 | Peru | Smoking | Per year | 3,933 | | |
| | Upper middle income | Russia | Krasnoyarsk region | Artyukhov IP, 2016 | Male | Smoking | Per year | 119 | 1,338 | |
| | Upper middle income | Russia | Krasnoyarsk region | Artyukhov IP, 2016 | Female | Smoking | Per year | 36 | 476 | |
| | Upper middle income | Russia | National | Yagudra, 2018 | 2009-2016 | Smoking | Per year | 10,831 | | |

## 3.7 Chronic bronchitis, national + city and region, by income level

**Table S3.7** Disease burden of chronic bronchitis at national and subnational level

| Attribution | Income group | Country | Area | Reference ID | Subgroup | Attribution (detail) | Unit | Mortality | Morbidity (DALY) | Years of life lost |
|---|---|---|---|---|---|---|---|---|---|---|
| Air pollution | | India | Mumbai | Maji KJ, 2017a | Mumbai, 1995 | $PM_{10}$ pollution | Per year | | 172,244 | |
| Air pollution | | India | Mumbai | Maji KJ, 2017a | Mumbai, 2000 | $PM_{10}$ pollution | Per year | | 185,425 | |

| Attribution | Income group | Country | Area | Reference ID | Subgroup | Attribution (detail) | Unit | Mortality | Morbidity (DALY) | Years of life lost |
|---|---|---|---|---|---|---|---|---|---|---|
| | Lower middle income | | Mumbai | | Mumbai, 2005 | $PM_{10}$ pollution | Per year | | 178,223 | |
| | | | Mumbai | | Mumbai, 2010 | $PM_{10}$ pollution | Per year | | 225,100 | |
| | | | Mumbai | | Mumbai, 2015 | $PM_{10}$ pollution | Per year | | 235,833 | |
| | | | Delhi | | Delhi, 1995 | $PM_{10}$ pollution | Per year | | 175,200 | |
| | | | Delhi | | Delhi, 2000 | $PM_{10}$ pollution | Per year | | 199,693 | |
| | | | Delhi | | Delhi, 2005 | $PM_{10}$ pollution | Per year | | 231,787 | |
| | | | Delhi | | Delhi, 2010 | $PM_{10}$ pollution | Per year | | 297,926 | |
| | | | Delhi | | Delhi, 2015 | $PM_{10}$ pollution | Per year | | 355,053 | |
| | | Iran | Qom province | Fard RF, 2016 | $PM_{10}$ | $PM_{10}$ | Per 2000 cases or years of life lost/year | | 1 | |
| | | | Qom province | | Nitrate | Nitrate | Per 2000 cases or years of life lost/year | | 13 | |
| | Upper middle income | China | National | Maji KJ, 2017c | Chronic bronchits | $PM_{10}$ | Per year | | 4,350,249 | |

## 3.8 Other diseases, national + city and region, by income level

**Table S3.8** Disease burden of other diseases at national and subnational level

| Attribution | Income group | Country | Area | Reference ID | Subgroup | Attribution (detail) | Disease | Unit | Mortality | Morbidity (DALY) | Years of life lost |
|---|---|---|---|---|---|---|---|---|---|---|---|
| COPD associated malnutrition | Upper middle income | China | National | Linthicum MT, 2015 | Overall population (age) | COPD associated malnutrition | COPD associated malnutrition | Per year | 190,953 | 1,315,276 | |
| | | | National | | 0-14 years (age) | COPD associated malnutrition | COPD associated malnutrition | Per year | 12 | 458 | |
| | | | National | | 15-59 years (age) | COPD associated malnutrition | COPD associated malnutrition | Per year | 7,516 | 339,548 | |
| | | | National | | 60+ years (age) | COPD associated malnutrition | COPD associated malnutrition | Per year | 183,425 | 975,270 | |
