# Supplementary material for: Economic burden of chronic obstructive pulmonary disease and post-tuberculosis sequelae in low- and middle-income countries: a database compiled from a systematic review and meta-analysis
Source: BMJ Public Health. 2024 Jul 30;2(1):e000441. doi: 10.1136/bmjph-2023-000441 (PMC11816951; doi:10.1136/bmjph-2023-000441)
Supplement: online supplemental file 4 [file bmjph-2-1-s004.pdf]

The economic burden of chronic obstructive pulmonary disease and post-tuberculosis sequelae in low- and middle-income countries: a database compiled from a systematic review and meta-analysis

Yuling Lin[1], Alexandra Walker[2,3], Marguerite Batta[2,3], Sierra Ottilie-Kovelman[2,4], Anna Duchenko[2,3], Curdin Brugger[2,3], Olivia Keiser[1], Robert S. Wallis[5], Klaus Reither[2,3], Fabrizio Tediosi[2,3] , Marina Antillon[2,3*]

1 Institute of Global Health, University of Geneva, 1205 Geneva, Switzerland
2 Swiss Tropical and Public Health Institute (Swiss TPH), Kreuzstrasse 2, 4123 Allschwil, Switzerland
3 University of Basel, Peterspl. 1, 4001 Basel, Switzerland
4 Yale School of Public Health, New Haven, Connecticut 06510, United States
5 The Aurum Institute, Johannesburg, South Africa
*Corresponding author

Supplemental Figures

Contents

4-I. Hospitalization costs 1
4.1 COPD by continent 1
4.2 COPD by calculation unit 1
4.3 AECOPD by country income group 2
4.4 AECOPD by continent 3
4.5 AECOPD by country 4
4.6 AECOPD by calculation unit 5
4-II Medication costs 6
4.7 COPD by country income group 6
4.8 COPD by country 7
4.9 AECOPD by country income group 8
4.10 AECOPD by country 9
4-III Outpatient costs 10
4.11 COPD by country income group 10
4.12 COPD by country 11

This supplementary material documents forest plots of meta-analysis and W denotes weight.

4-I. Hospitalization costs

4.1 COPD by continent

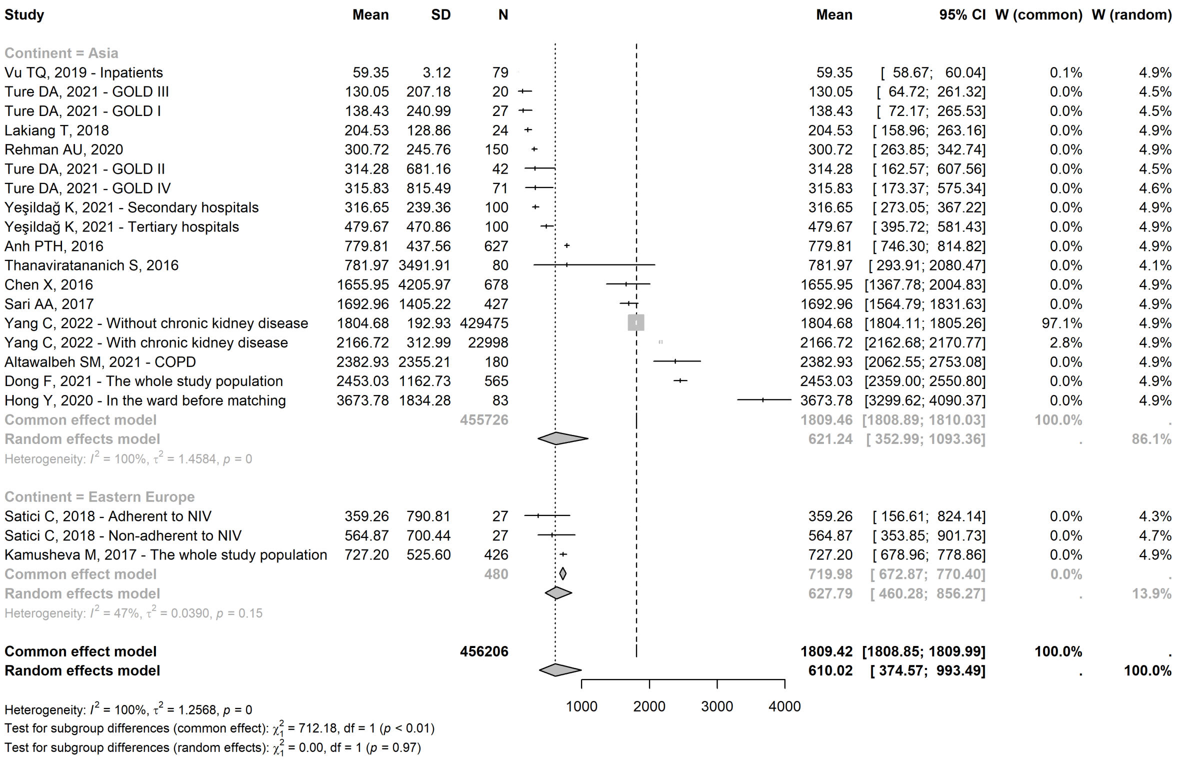

Figure S4.1 Meta-analysis of hospitalization costs in 2021 USD of COPD by continent

4.2 COPD by calculation unit

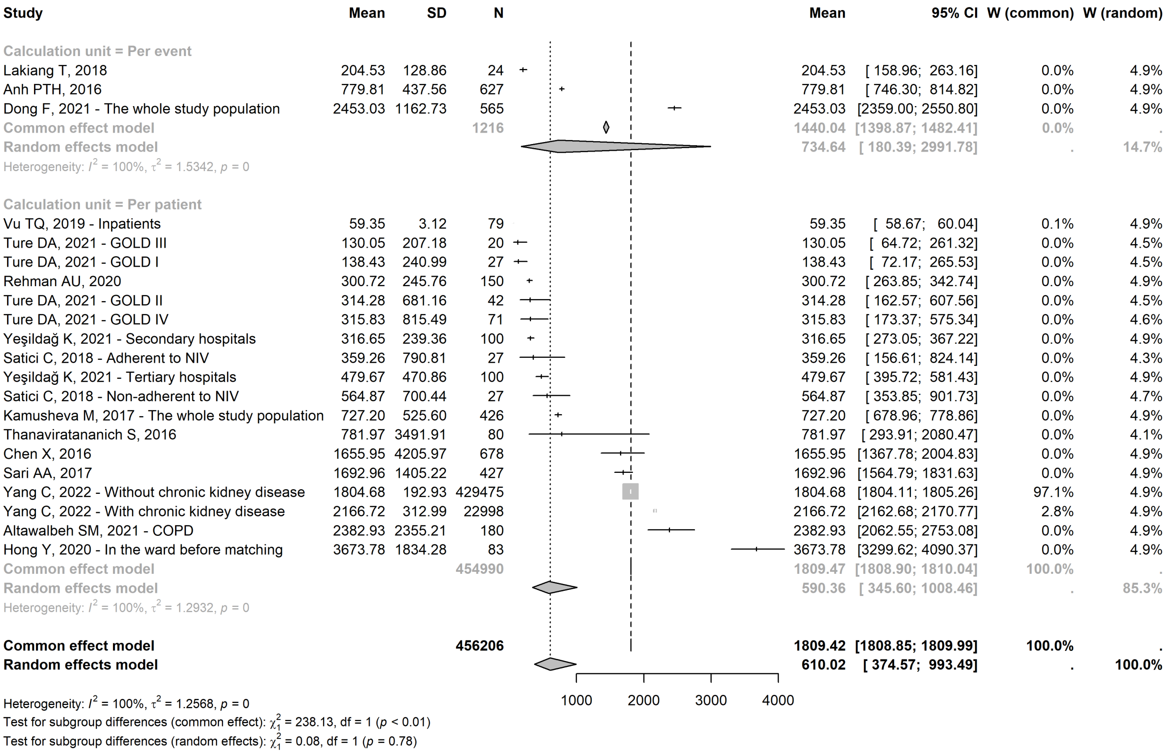

Figure S4.2 Meta-analysis of hospitalization costs in 2021 USD of COPD by calculation unit

4.3 AECOPD by country income group

Study	Mean	SD	N	Mean	95% CI	W (common)	W (random)
Income group = Upper middle income							
Deniz S, 2016 - The whole study population	190.94	516.78	5237	190.94	[177.44; 205.46]	0.2%	1.6%
Pavlovic R, 2020 - Patients with only one exacerbation over the course of one year	325.94	206.12	110	325.94	[289.61; 366.83]	0.1%	1.6%
Yilmaz C, 2021 - Index hospitalitation without readmission	333.97	246.17	76	333.97	[282.97; 394.16]	0.0%	1.6%
Tanriverdi H, 2013	367.48	206.46	236	367.48	[342.06; 394.79]	0.2%	1.6%
Pavlovic R, 2020 - Patients with frequent exacerbations	431.87	263.78	64	431.87	[371.85; 501.58]	0.0%	1.6%
Yilmaz C, 2021 - The index hospitalization	455.62	521.03	34	455.62	[310.22; 669.18]	0.0%	1.5%
Örnek T, 2014 - AECOPD without coal worker pneumoconiosis	659.77	581.11	51	659.77	[518.10; 840.18]	0.0%	1.5%
Yilmaz C, 2021 - Readmission	682.28	855.57	34	682.28	[447.62; 1039.97]	0.0%	1.5%
Inchai J, 2020 - Without comorbidities	861.42	852.64	179	861.42	[745.15; 995.84]	0.0%	1.6%
Varol Y, 2013	903.79	842.22	376	903.79	[822.55; 993.06]	0.1%	1.6%
Pothirat C, 2015 - Requiring mechanical ventilator managed by pulmonologist	1024.22	485.73	137	1024.22	[946.03; 1108.88]	0.2%	1.6%
Chen R, 2020 - Low adherence group (before index), oral therapy	1076.15	3629.58	7536	1076.15	[997.25; 1161.30]	0.2%	1.6%
Chen R, 2020 - Low adherence group (before index), combined	1078.33	2626.25	2516	1078.33	[980.44; 1185.99]	0.1%	1.6%
Li F, 2018 - Low-cost group	1174.76	232.43	94	1174.76	[1128.70; 1222.70]	0.6%	1.6%
Chen R, 2020 - Low adherence group (before index), inhale bronchodilator	1231.60	4730.73	395	1231.60	[843.26; 1798.79]	0.0%	1.5%
Inchai J, 2020 - With comorbidities	1371.71	1570.44	560	1371.71	[1247.62; 1508.15]	0.1%	1.6%
Örnek T, 2014 - AECOPD with coal worker pneumoconiosis	1437.90	1930.43	51	1437.90	[994.74; 2078.49]	0.0%	1.5%
Chen R, 2020 - High adherence group (before index), combined	1459.87	3078.45	903	1459.87	[1272.28; 1675.12]	0.1%	1.6%
Cui Y, 2022 - AECOPD without coronary artery disease	1496.20	953.98	3242	1496.20	[1463.72; 1529.40]	2.1%	1.6%
E W, 2021 - AECOPD without pneumoconiosis	1534.55	526.51	62	1534.55	[1408.93; 1671.37]	0.1%	1.6%
Cui Y, 2021b - Eosinophilic (after propensity score matching)	1551.43	681.56	650	1551.43	[1499.91; 1604.72]	0.9%	1.6%
Cui Y, 2022 - AECOPD with coronary artery disease	1570.56	1053.90	664	1570.56	[1492.41; 1652.80]	0.4%	1.6%
Chen R, 2020 - High adherence group (before index), inhale bronchodilator	1577.27	2315.36	225	1577.27	[1301.98; 1910.77]	0.0%	1.6%
Mao X, 2021 - Without diabetes	1626.44	784.28	4762	1626.44	[1604.31; 1648.87]	5.4%	1.6%
You L, 2021 - Without pulmonary heart disease	1630.70	792.63	3277	1630.70	[1603.79; 1658.07]	3.7%	1.6%
Cui Y, 2021b - Non-eosinophilic (after propensity score matching)	1699.82	785.15	650	1699.82	[1640.52; 1761.26]	0.8%	1.6%
You L, 2021 - With pulmonary heart disease	1701.38	783.68	1109	1701.38	[1655.88; 1748.14]	1.4%	1.6%
Zeng Q, 2021 - Total study population, eosinophilic group	1830.14	910.14	312	1830.14	[1731.89; 1933.97]	0.3%	1.6%
Mao X, 2021 - With diabetes	1856.97	895.48	572	1856.97	[1785.02; 1931.83]	0.7%	1.6%
Zeng Q, 2021 - Total study population, non-eosinophilic group	2205.85	1139.04	391	2205.85	[2095.79; 2321.69]	0.4%	1.6%
E W, 2021 - AECOPD with pneumoconiosis	2242.18	691.94	65	2242.18	[2080.12; 2416.86]	0.2%	1.6%
Pothirat C, 2015 - Requiring mechanical ventilator managed by internist	2626.49	1429.80	71	2626.49	[2314.11; 2981.04]	0.1%	1.6%
Liang L, 2020b - 2008	2687.31	3797.15	23194	2687.31	[2638.88; 2736.62]	3.1%	1.6%
Li F, 2018 - High-cost group	2818.36	2022.91	94	2818.36	[2437.70; 3258.46]	0.0%	1.6%
Liang L, 2020b - 2009	3113.40	5137.80	25341	3113.40	[3050.78; 3177.30]	2.5%	1.6%
Liang L, 2020b - 2012	3198.46	4919.29	36539	3198.46	[3148.42; 3249.30]	4.1%	1.6%
Gong C, 2020 - Serum procalcitonin levels <0.1 ng/ml	3206.37	1977.61	55	3206.37	[2724.10; 3774.02]	0.0%	1.6%
Liang L, 2020b - 2013	3227.94	4616.18	40315	3227.94	[3183.19; 3273.32]	5.2%	1.6%
Liang L, 2020b - 2011	3235.03	5650.74	29870	3235.03	[3171.58; 3299.75]	2.6%	1.6%
Liang L, 2020b - 2014	3281.67	4544.23	45681	3281.67	[3240.26; 3323.61]	6.3%	1.6%
Wang S, 2021 - Peripheral blood eosinophils ≥2%	3315.03	1320.79	146	3315.03	[3107.56; 3536.35]	0.2%	1.6%
Liang L, 2020a - 2013	3315.70	4666.98	28642	3315.70	[3262.09; 3370.19]	3.8%	1.6%
Liang L, 2020a - 2009	3328.50	5289.05	21190	3328.50	[3258.04; 3400.48]	2.2%	1.6%
Liang L, 2020a - 2012	3341.14	5010.10	27301	3341.14	[3282.24; 3401.10]	3.2%	1.6%
Liang L, 2020b - 2017	3347.79	3619.22	35476	3347.79	[3310.34; 3385.66]	8.0%	1.6%
Liang L, 2020a - 2014	3357.98	4580.06	30411	3357.98	[3306.90; 3409.85]	4.3%	1.6%
Liang L, 2020b - 2010	3366.05	6706.28	29492	3366.05	[3290.38; 3443.46]	2.0%	1.6%
Liang L, 2020b - 2015	3371.04	4065.71	34491	3371.04	[3328.40; 3414.22]	6.3%	1.6%
Liang L, 2020a - 2017	3388.81	3630.02	23778	3388.81	[3342.98; 3435.26]	5.5%	1.6%
Liang L, 2020a - 2011	3394.87	5776.03	23863	3394.87	[3322.37; 3468.95]	2.2%	1.6%
Liang L, 2020b - 2016	3406.12	3822.62	37403	3406.12	[3367.60; 3445.08]	7.9%	1.6%
Liang L, 2020a - 2016	3464.44	3843.28	24588	3464.44	[3416.73; 3512.81]	5.3%	1.6%
Liang L, 2020a - 2015	3465.11	4104.20	23257	3465.11	[3412.76; 3518.26]	4.4%	1.6%
Liang L, 2020a - 2010	3574.43	6905.13	23652	3574.43	[3487.50; 3663.52]	1.7%	1.6%
Chen R, 2020 - High adherence group (before index), oral therapy	4069.82	6684.12	133	4069.82	[3078.61; 5380.16]	0.0%	1.5%
Li M, 2018	4170.91	7559.48	1943	4170.91	[3847.97; 4520.95]	0.2%	1.6%
Wang S, 2021 - Peripheral blood eosinophils <2%	4191.29	2393.62	409	4191.29	[3965.62; 4429.80]	0.3%	1.6%
Gong C, 2020 - Serum procalcitonin levels ≥0.1 ng/ml	4491.80	3655.67	55	4491.80	[3622.51; 5569.69]	0.0%	1.5%
Fan L, 2014 - NPPV success group	9970.16	10431.98	260	9970.16	[8779.46; 11322.35]	0.1%	1.6%
Fan L, 2014 - NPPV failure group	15442.31	14120.67	55	15442.31	[12127.17; 19663.69]	0.0%	1.5%
Common effect model			602275	2875.86	[2866.70; 2885.05]	99.8%	.
Random effects model				1919.66	[1559.45; 2363.08]	.	93.8%
Heterogeneity: I^2 = 100%, τ^2 = 0.6696, p = 0							
Income group = Lower middle income							
Torabipour A, 2016 - Length of stay ≤ 9 days	302.85	440.51	126	302.85	[234.92; 390.42]	0.0%	1.5%
Koul PA, 2019	670.28	304.88	129	670.28	[619.68; 725.02]	0.2%	1.6%
Ngo CQ, 2019 - The whole study population	853.80	601.86	57	853.80	[711.02; 1025.25]	0.0%	1.6%
Torabipour A, 2016 - Length of stay > 9 days	1941.64	2344.13	39	1941.64	[1329.27; 2836.12]	0.0%	1.5%
Common effect model			351	677.56	[632.86; 725.42]	0.2%	.
Random effects model				755.06	[361.87; 1575.46]	.	6.2%
Heterogeneity: I^2 = 96%, τ^2 = 0.5475, p < 0.01							
**Common effect model**			**602626**	**2866.81**	**[2857.69; 2875.96]**	**100.0%**	.
**Random effects model**				**1812.40**	**[1474.19; 2228.19]**	.	**100.0%**

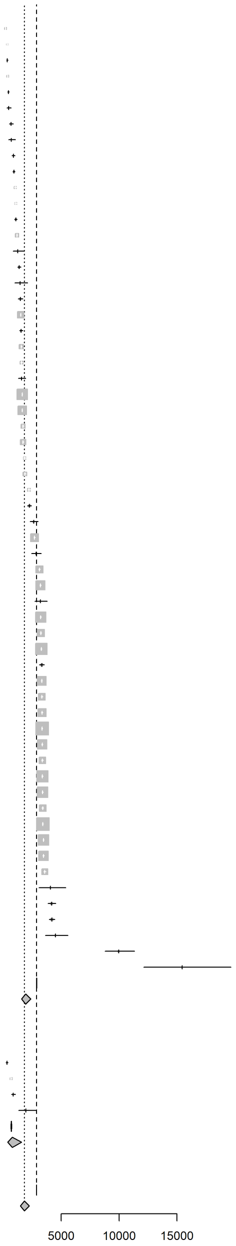

Heterogeneity: I^2 = 100%, τ^2 = 0.7051, p = 0
Test for subgroup differences (common effect): χ^2_1 = 1719.35, df = 1 (p = 0)
Test for subgroup differences (random effects): χ^2_1 = 5.73, df = 1 (p = 0.02)

Figure S4.3 Meta-analysis of hospitalization costs in 2021 USD of AECOPD by income group

4.4 AECOPD by continent

Study	Mean	SD	N	Mean	95% CI	W (common)	W (random)
Continent = Eastern Europe							
Deniz S, 2016 - The whole study population	190.94	516.78	5237	190.94	[177.44; 205.46]	0.2%	1.6%
Pavlovic R, 2020 - Patients with only one exacerbation over the course of one year	325.94	206.12	110	325.94	[289.61; 366.83]	0.1%	1.6%
Pavlovic R, 2020 - Patients with frequent exacerbations	431.87	263.78	64	431.87	[371.85; 501.58]	0.0%	1.6%
Varol Y, 2013	903.79	842.22	376	903.79	[822.55; 993.06]	0.1%	1.6%
Common effect model			5787	348.68	[331.98; 366.23]	0.4%	.
Random effects model				394.62	[208.77; 745.89]	.	6.3%
Heterogeneity: I^2 = 100%, τ^2 = 0.4188, p < 0.01							
Continent = Asia							
Torabipour A, 2016 - Length of stay ≤ 9 days	302.85	440.51	126	302.85	[234.92; 390.42]	0.0%	1.5%
Yilmaz C, 2021 - Index hospitalitation without readmission	333.97	246.17	76	333.97	[282.97; 394.16]	0.0%	1.6%
Tanriverdi H, 2013	367.48	206.46	236	367.48	[342.06; 394.79]	0.2%	1.6%
Yilmaz C, 2021 - The index hospitalization	455.62	521.03	34	455.62	[310.22; 669.18]	0.0%	1.5%
Örnek T, 2014 - AECOPD without coal worker pneumoconiosis	659.77	581.11	51	659.77	[518.10; 840.18]	0.0%	1.5%
Koul PA, 2019	670.28	304.88	129	670.28	[619.68; 725.02]	0.2%	1.6%
Yilmaz C, 2021 - Readmission	682.28	855.57	34	682.28	[447.62; 1039.97]	0.0%	1.5%
Ngo CQ, 2019 - The whole study population	853.80	601.86	57	853.80	[711.02; 1025.25]	0.0%	1.6%
Inchai J, 2020 - Without comorbidities	861.42	852.64	179	861.42	[745.15; 995.84]	0.0%	1.6%
Pothirat C, 2015 - Requiring mechanical ventilator managed by pulmonologist	1024.22	485.73	137	1024.22	[946.03; 1108.88]	0.2%	1.6%
Chen R, 2020 - Low adherence group (before index), oral therapy	1076.15	3629.58	7536	1076.15	[997.25; 1161.30]	0.2%	1.6%
Chen R, 2020 - Low adherence group (before index), combined	1078.33	2626.25	2516	1078.33	[980.44; 1185.99]	0.1%	1.6%
Li F, 2018 - Low-cost group	1174.76	232.43	94	1174.76	[1128.70; 1222.70]	0.6%	1.6%
Chen R, 2020 - Low adherence group (before index), inhale bronchodilator	1231.60	4730.73	395	1231.60	[843.26; 1798.79]	0.0%	1.5%
Inchai J, 2020 - With comorbidities	1371.71	1570.44	560	1371.71	[1247.62; 1508.15]	0.1%	1.6%
Örnek T, 2014 - AECOPD with coal worker pneumoconiosis	1437.90	1930.43	51	1437.90	[994.74; 2078.49]	0.0%	1.5%
Chen R, 2020 - High adherence group (before index), combined	1459.87	3078.45	903	1459.87	[1272.28; 1675.12]	0.1%	1.6%
Cui Y, 2022 - AECOPD without coronary artery disease	1496.20	953.98	3242	1496.20	[1463.72; 1529.40]	2.1%	1.6%
E W, 2021 - AECOPD without pneumoconiosis	1534.55	526.51	62	1534.55	[1408.93; 1671.37]	0.1%	1.6%
Cui Y, 2021b - Eosinophilic (after propensity score matching)	1551.43	681.56	650	1551.43	[1499.91; 1604.72]	0.9%	1.6%
Cui Y, 2022 - AECOPD with coronary artery disease	1570.56	1053.90	664	1570.56	[1492.41; 1652.80]	0.4%	1.6%
Chen R, 2020 - High adherence group (before index), inhale bronchodilator	1577.27	2315.36	225	1577.27	[1301.98; 1910.77]	0.0%	1.6%
Mao X, 2021 - Without diabetes	1626.44	784.28	4762	1626.44	[1604.31; 1648.87]	5.4%	1.6%
You L, 2021 - Without pulmonary heart disease	1630.70	792.63	3277	1630.70	[1603.79; 1658.07]	3.7%	1.6%
Cui Y, 2021b - Non-eosinophilic (after propensity score matching)	1699.82	785.15	650	1699.82	[1640.52; 1761.26]	0.8%	1.6%
You L, 2021 - With pulmonary heart disease	1701.38	783.68	1109	1701.38	[1655.88; 1748.14]	1.4%	1.6%
Zeng Q, 2021 - Total study population, eosinophilic group	1830.14	910.14	312	1830.14	[1731.89; 1933.97]	0.3%	1.6%
Mao X, 2021 - With diabetes	1856.97	895.48	572	1856.97	[1785.02; 1931.83]	0.7%	1.6%
Torabipour A, 2016 - Length of stay > 9 days	1941.64	2344.13	39	1941.64	[1329.27; 2836.12]	0.0%	1.5%
Zeng Q, 2021 - Total study population, non-eosinophilic group	2205.85	1139.04	391	2205.85	[2095.79; 2321.69]	0.4%	1.6%
E W, 2021 - AECOPD with pneumoconiosis	2242.18	691.94	65	2242.18	[2080.12; 2416.86]	0.2%	1.6%
Pothirat C, 2015 - Requiring mechanical ventilator managed by internist	2626.49	1429.80	71	2626.49	[2314.11; 2981.04]	0.1%	1.6%
Liang L, 2020b - 2008	2687.31	3797.15	23194	2687.31	[2638.88; 2736.62]	3.1%	1.6%
Li F, 2018 - High-cost group	2818.36	2022.91	94	2818.36	[2437.70; 3258.46]	0.0%	1.6%
Liang L, 2020b - 2009	3113.40	5137.80	25341	3113.40	[3050.78; 3177.30]	2.5%	1.6%
Liang L, 2020b - 2012	3198.46	4919.29	36539	3198.46	[3148.42; 3249.30]	4.1%	1.6%
Gong C, 2020 - Serum procalcitonin levels <0.1 ng/ml	3206.37	1977.61	55	3206.37	[2724.10; 3774.02]	0.0%	1.6%
Liang L, 2020b - 2013	3227.94	4616.18	40315	3227.94	[3183.19; 3273.32]	5.2%	1.6%
Liang L, 2020b - 2011	3235.03	5650.74	29870	3235.03	[3171.58; 3299.75]	2.6%	1.6%
Liang L, 2020b - 2014	3281.67	4544.23	45681	3281.67	[3240.26; 3323.61]	6.3%	1.6%
Wang S, 2021 - Peripheral blood eosinophils ≥2%	3315.03	1320.79	146	3315.03	[3107.56; 3536.35]	0.2%	1.6%
Liang L, 2020a - 2013	3315.70	4666.98	28642	3315.70	[3262.09; 3370.19]	3.8%	1.6%
Liang L, 2020a - 2009	3328.50	5289.05	21190	3328.50	[3258.04; 3400.48]	2.2%	1.6%
Liang L, 2020a - 2012	3341.14	5010.10	27301	3341.14	[3282.24; 3401.10]	3.2%	1.6%
Liang L, 2020b - 2017	3347.79	3619.22	35476	3347.79	[3310.34; 3385.66]	8.0%	1.6%
Liang L, 2020a - 2014	3357.98	4580.06	30411	3357.98	[3306.90; 3409.85]	4.3%	1.6%
Liang L, 2020b - 2010	3366.05	6706.28	29492	3366.05	[3290.38; 3443.46]	2.0%	1.6%
Liang L, 2020b - 2015	3371.04	4065.71	34491	3371.04	[3328.40; 3414.22]	6.3%	1.6%
Liang L, 2020a - 2017	3388.81	3630.02	23778	3388.81	[3342.98; 3435.26]	5.5%	1.6%
Liang L, 2020a - 2011	3394.87	5776.03	23863	3394.87	[3322.37; 3468.95]	2.2%	1.6%
Liang L, 2020b - 2016	3406.12	3822.62	37403	3406.12	[3367.60; 3445.08]	7.9%	1.6%
Liang L, 2020a - 2016	3464.44	3843.28	24588	3464.44	[3416.73; 3512.81]	5.3%	1.6%
Liang L, 2020a - 2015	3465.11	4104.20	23257	3465.11	[3412.76; 3518.26]	4.4%	1.6%
Liang L, 2020a - 2010	3574.43	6905.13	23652	3574.43	[3487.50; 3663.52]	1.7%	1.6%
Chen R, 2020 - High adherence group (before index), oral therapy	4069.82	6684.12	133	4069.82	[3078.61; 5380.16]	0.0%	1.5%
Li M, 2018	4170.91	7559.48	1943	4170.91	[3847.97; 4520.95]	0.2%	1.6%
Wang S, 2021 - Peripheral blood eosinophils <2%	4191.29	2393.62	409	4191.29	[3965.62; 4429.80]	0.3%	1.6%
Gong C, 2020 - Serum procalcitonin levels ≥0.1 ng/ml	4491.80	3655.67	55	4491.80	[3622.51; 5569.69]	0.0%	1.5%
Fan L, 2014 - NPPV success group	9970.16	10431.98	260	9970.16	[8779.46; 11322.35]	0.1%	1.6%
Fan L, 2014 - NPPV failure group	15442.31	14120.67	55	15442.31	[12127.17; 19663.69]	0.0%	1.5%
Common effect model			596839	2892.50	[2883.27; 2901.75]	99.6%	.
Random effects model				2008.67	[1660.53; 2429.79]	.	93.7%
Heterogeneity: I^2 = 100%, τ^2 = 0.5601, p = 0							
**Common effect model**			**602626**	**2866.81**	**[2857.69; 2875.96]**	**100.0%**	**.**
**Random effects model**				**1812.40**	**[1474.19; 2228.19]**	**.**	**100.0%**

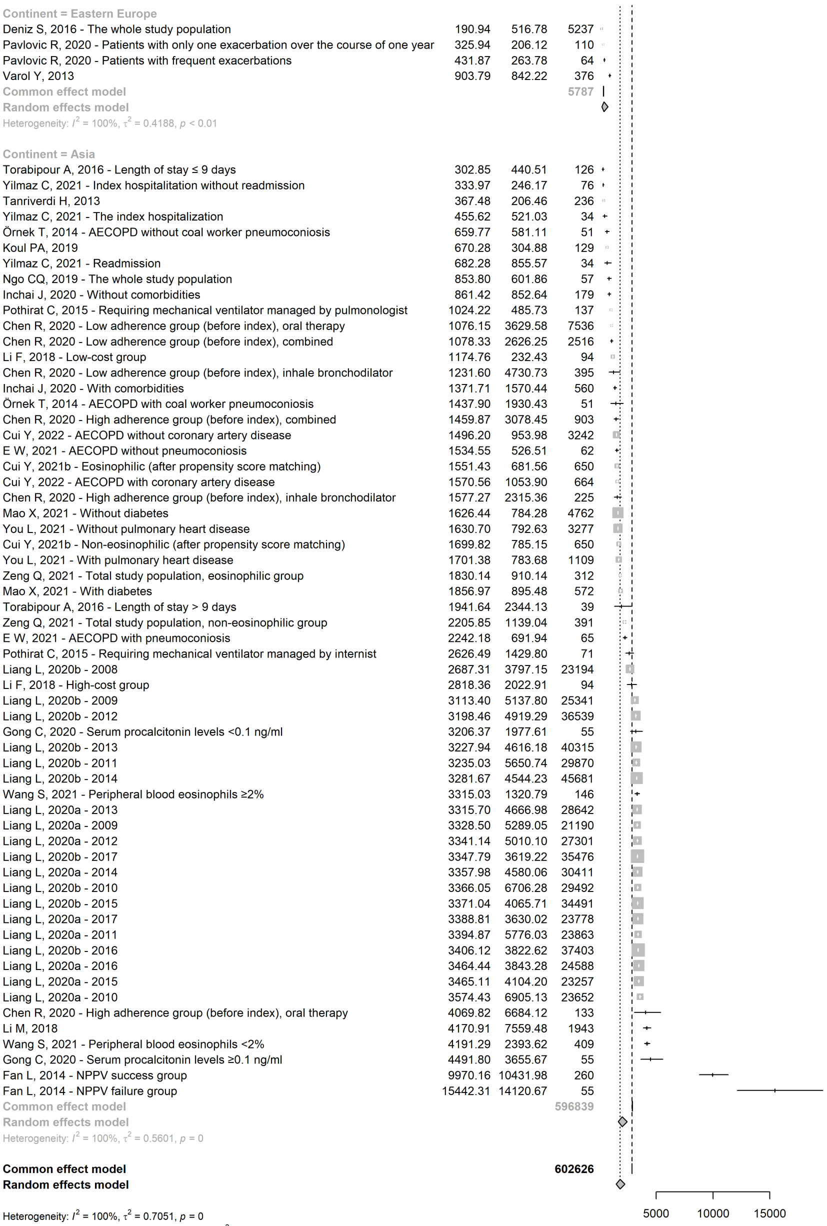

Heterogeneity: I^2 = 100%, τ^2 = 0.7051, p = 0
Test for subgroup differences (common effect): χ^2_1 = 7107.38, df = 1 (p = 0)
Test for subgroup differences (random effects): χ^2_1 = 23.04, df = 1 (p < 0.01)

Figure S4.4 Meta-analysis of hospitalization costs in 2021 USD of AECOPD by continent

4.5 AECOPD by country

Study	Mean	SD	N	Mean	95% CI	W (common)	W (random)
**Country = Turkey**							
Deniz S, 2016 - The whole study population	190.94	516.78	5237	190.94	[177.44; 205.46]	0.2%	1.6%
Yilmaz C, 2021 - Index hospitalitation without readmission	333.97	246.17	76	333.97	[282.97; 394.16]	0.0%	1.6%
Tanriverdi H, 2013	367.48	206.46	236	367.48	[342.06; 394.79]	0.2%	1.6%
Yilmaz C, 2021 - The index hospitalization	455.62	521.03	34	455.62	[310.22; 669.18]	0.0%	1.5%
Örnek T, 2014 - AECOPD without coal worker pneumoconiosis	659.77	581.11	51	659.77	[518.10; 840.18]	0.0%	1.5%
Yilmaz C, 2021 - Readmission	682.28	855.57	34	682.28	[447.62; 1039.97]	0.0%	1.5%
Varol Y, 2013	903.79	842.22	376	903.79	[822.55; 993.06]	0.1%	1.6%
Örnek T, 2014 - AECOPD with coal worker pneumoconiosis	1437.90	1930.43	51	1437.90	[994.74; 2078.49]	0.0%	1.5%
Common effect model			6095	368.20	[353.05; 383.99]	0.6%	.
Random effects model				523.71	[336.66; 814.70]	.	12.3%
Heterogeneity: $I^2 = 99\%$, $\tau^2 = 0.3889$, $p < 0.01$							
**Country = India**							
Koul PA, 2019	670.28	304.88	129	670.28	[619.68; 725.02]	0.2%	1.6%
**Country = Vietnam**							
Ngo CQ, 2019 - The whole study population	853.80	601.86	57	853.80	[711.02; 1025.25]	0.0%	1.6%
**Country = Iran**							
Torabipour A, 2016 - Length of stay ≤ 9 days	302.85	440.51	126	302.85	[234.92; 390.42]	0.0%	1.5%
Torabipour A, 2016 - Length of stay > 9 days	1941.64	2344.13	39	1941.64	[1329.27; 2836.12]	0.0%	1.5%
Common effect model			165	538.75	[436.28; 665.28]	0.0%	.
Random effects model				762.59	[123.46; 4710.49]	.	3.0%
Heterogeneity: $I^2 = 98\%$, $\tau^2 = 1.6991$, $p < 0.01$							
**Country = China**							
Chen R, 2020 - Low adherence group (before index), oral therapy	1076.15	3629.58	7536	1076.15	[997.25; 1161.30]	0.2%	1.6%
Chen R, 2020 - Low adherence group (before index), combined	1078.33	2626.25	2516	1078.33	[980.44; 1185.99]	0.1%	1.6%
Li F, 2018 - Low-cost group	1174.76	232.43	94	1174.76	[1128.70; 1222.70]	0.6%	1.6%
Chen R, 2020 - Low adherence group (before index), inhale bronchodilator	1231.60	4730.73	395	1231.60	[843.26; 1798.79]	0.0%	1.5%
Chen R, 2020 - High adherence group (before index), combined	1459.87	3078.45	903	1459.87	[1272.28; 1675.12]	0.1%	1.6%
Cui Y, 2022 - AECOPD without coronary artery disease	1496.20	953.98	3242	1496.20	[1463.72; 1529.40]	2.1%	1.6%
E W, 2021 - AECOPD without pneumoconiosis	1534.55	526.51	62	1534.55	[1408.93; 1671.37]	0.1%	1.6%
Cui Y, 2021b - Eosinophilic (after propensity score matching)	1551.43	681.56	650	1551.43	[1499.91; 1604.72]	0.9%	1.6%
Cui Y, 2022 - AECOPD with coronary artery disease	1570.56	1053.90	664	1570.56	[1492.41; 1652.80]	0.4%	1.6%
Chen R, 2020 - High adherence group (before index), inhale bronchodilator	1577.27	2315.36	225	1577.27	[1301.98; 1910.77]	0.0%	1.6%
Mao X, 2021 - Without diabetes	1626.44	784.28	4762	1626.44	[1604.31; 1648.87]	5.4%	1.6%
You L, 2021 - Without pulmonary heart disease	1630.70	792.63	3277	1630.70	[1603.79; 1658.07]	3.7%	1.6%
Cui Y, 2021b - Non-eosinophilic (after propensity score matching)	1699.82	785.15	650	1699.82	[1640.52; 1761.26]	0.8%	1.6%
You L, 2021 - With pulmonary heart disease	1701.38	783.68	1109	1701.38	[1655.88; 1748.14]	1.4%	1.6%
Zeng Q, 2021 - Total study population, eosinophilic group	1830.14	910.14	312	1830.14	[1731.89; 1933.97]	0.3%	1.6%
Mao X, 2021 - With diabetes	1856.97	895.48	572	1856.97	[1785.02; 1931.83]	0.7%	1.6%
Zeng Q, 2021 - Total study population, non-eosinophilic group	2205.85	1139.04	391	2205.85	[2095.79; 2321.69]	0.4%	1.6%
E W, 2021 - AECOPD with pneumoconiosis	2242.18	691.94	65	2242.18	[2080.12; 2416.86]	0.2%	1.6%
Liang L, 2020b - 2008	2687.31	3797.15	23194	2687.31	[2638.88; 2736.62]	3.1%	1.6%
Li F, 2018 - High-cost group	2818.36	2022.91	94	2818.36	[2437.70; 3258.46]	0.0%	1.6%
Liang L, 2020b - 2009	3113.40	5137.80	25341	3113.40	[3050.78; 3177.30]	2.5%	1.6%
Liang L, 2020b - 2012	3198.46	4919.29	36539	3198.46	[3148.42; 3249.30]	4.1%	1.6%
Gong C, 2020 - Serum procalcitonin levels <0.1 ng/ml	3206.37	1977.61	55	3206.37	[2724.10; 3774.02]	0.0%	1.6%
Liang L, 2020b - 2013	3227.94	4616.18	40315	3227.94	[3183.19; 3273.32]	5.2%	1.6%
Liang L, 2020b - 2011	3235.03	5650.74	29870	3235.03	[3171.58; 3299.75]	2.6%	1.6%
Liang L, 2020b - 2014	3281.67	4544.23	45681	3281.67	[3240.26; 3323.61]	6.3%	1.6%
Wang S, 2021 - Peripheral blood eosinophils ≥2%	3315.03	1320.79	146	3315.03	[3107.56; 3536.35]	0.2%	1.6%
Liang L, 2020a - 2013	3315.70	4666.98	28642	3315.70	[3262.09; 3370.19]	3.8%	1.6%
Liang L, 2020a - 2009	3328.50	5289.05	21190	3328.50	[3258.04; 3400.48]	2.2%	1.6%
Liang L, 2020a - 2012	3341.14	5010.10	27301	3341.14	[3282.24; 3401.10]	3.2%	1.6%
Liang L, 2020b - 2017	3347.79	3619.22	35476	3347.79	[3310.34; 3385.66]	8.0%	1.6%
Liang L, 2020a - 2014	3357.98	4580.06	30411	3357.98	[3306.90; 3409.85]	4.3%	1.6%
Liang L, 2020b - 2010	3366.05	6706.28	29492	3366.05	[3290.38; 3443.46]	2.0%	1.6%
Liang L, 2020b - 2015	3371.04	4065.71	34491	3371.04	[3328.40; 3414.22]	6.3%	1.6%
Liang L, 2020a - 2017	3388.81	3630.02	23778	3388.81	[3342.98; 3435.26]	5.5%	1.6%
Liang L, 2020a - 2011	3394.87	5776.03	23863	3394.87	[3322.37; 3468.95]	2.2%	1.6%
Liang L, 2020b - 2016	3406.12	3822.62	37403	3406.12	[3367.60; 3445.08]	7.9%	1.6%
Liang L, 2020a - 2016	3464.44	3843.28	24588	3464.44	[3416.73; 3512.81]	5.3%	1.6%
Liang L, 2020a - 2015	3465.11	4104.20	23257	3465.11	[3412.76; 3518.26]	4.4%	1.6%
Liang L, 2020a - 2010	3574.43	6905.13	23652	3574.43	[3487.50; 3663.52]	1.7%	1.6%
Chen R, 2020 - High adherence group (before index), oral therapy	4069.82	6684.12	133	4069.82	[3078.61; 5380.16]	0.0%	1.5%
Li M, 2018	4170.91	7559.48	1943	4170.91	[3847.97; 4520.95]	0.2%	1.6%
Wang S, 2021 - Peripheral blood eosinophils <2%	4191.29	2393.62	409	4191.29	[3965.62; 4429.80]	0.3%	1.6%
Gong C, 2020 - Serum procalcitonin levels ≥0.1 ng/ml	4491.80	3655.67	55	4491.80	[3622.51; 5569.69]	0.0%	1.5%
Fan L, 2014 - NPPV success group	9970.16	10431.98	260	9970.16	[8779.46; 11322.35]	0.1%	1.6%
Fan L, 2014 - NPPV failure group	15442.31	14120.67	55	15442.31	[12127.17; 19663.69]	0.0%	1.5%
Common effect model			595059	2927.05	[2917.68; 2936.46]	98.7%	.
Random effects model				2648.74	[2277.25; 3080.84]	.	72.2%
Heterogeneity: $I^2 = 100\%$, $\tau^2 = 0.2707$, $p = 0$							
**Country = Thailand**							
Inchai J, 2020 - Without comorbidities	861.42	852.64	179	861.42	[745.15; 995.84]	0.0%	1.6%
Pothirat C, 2015 - Requiring mechanical ventilator managed by pulmonologist	1024.22	485.73	137	1024.22	[946.03; 1108.88]	0.2%	1.6%
Inchai J, 2020 - With comorbidities	1371.71	1570.44	560	1371.71	[1247.62; 1508.15]	0.1%	1.6%
Pothirat C, 2015 - Requiring mechanical ventilator managed by internist	2626.49	1429.80	71	2626.49	[2314.11; 2981.04]	0.1%	1.6%
Common effect model			947	1274.42	[1210.67; 1341.53]	0.4%	.
Random effects model				1335.16	[827.31; 2154.78]	.	6.3%
Heterogeneity: $I^2 = 98\%$, $\tau^2 = 0.2351$, $p < 0.01$							
**Country = Serbia**							
Pavlovic R, 2020 - Patients with only one exacerbation over the course of one year	325.94	206.12	110	325.94	[289.61; 366.83]	0.1%	1.6%
Pavlovic R, 2020 - Patients with frequent exacerbations	431.87	263.78	64	431.87	[371.85; 501.58]	0.0%	1.6%
Common effect model			174	363.15	[330.98; 398.44]	0.1%	.
Random effects model				373.73	[283.68; 492.35]	.	3.1%
Heterogeneity: $I^2 = 88\%$, $\tau^2 = 0.0349$, $p < 0.01$							
**Common effect model**			**602626**	**2866.81**	**[2857.69; 2875.96]**	**100.0%**	**.**
**Random effects model**				**1812.40**	**[1474.19; 2228.19]**	**.**	**100.0%**

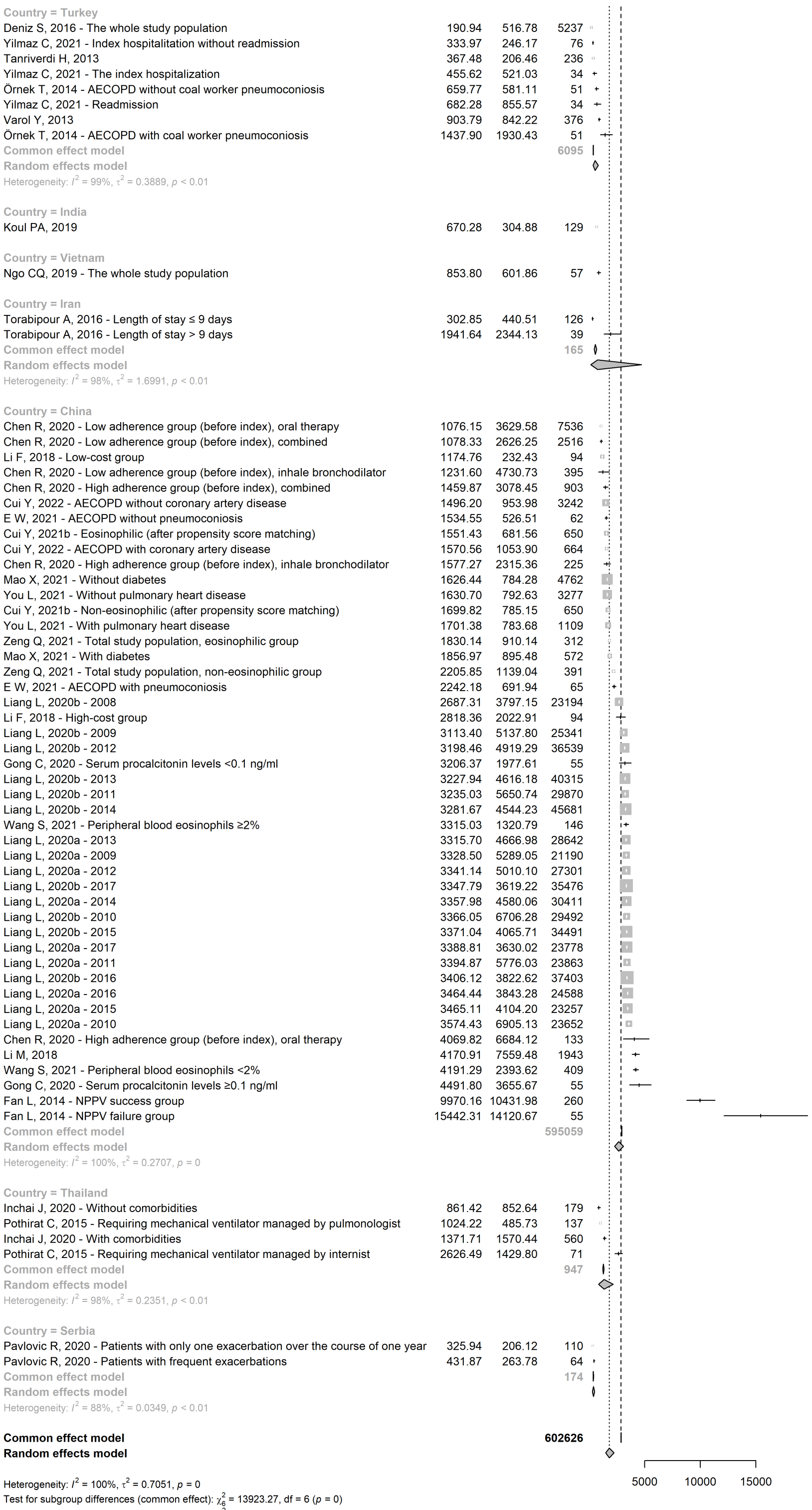

Heterogeneity: $I^2 = 100\%$, $\tau^2 = 0.7051$, $p = 0$
Test for subgroup differences (common effect): $\chi^2_6 = 13923.27$, df = 6 ($p = 0$)
Test for subgroup differences (random effects): $\chi^2_6 = 294.68$, df = 6 ($p < 0.01$)

Figure S4.5 Meta-analysis of hospitalization costs in 2021 USD of AECOPD by country

4.6 AECOPD by calculation unit

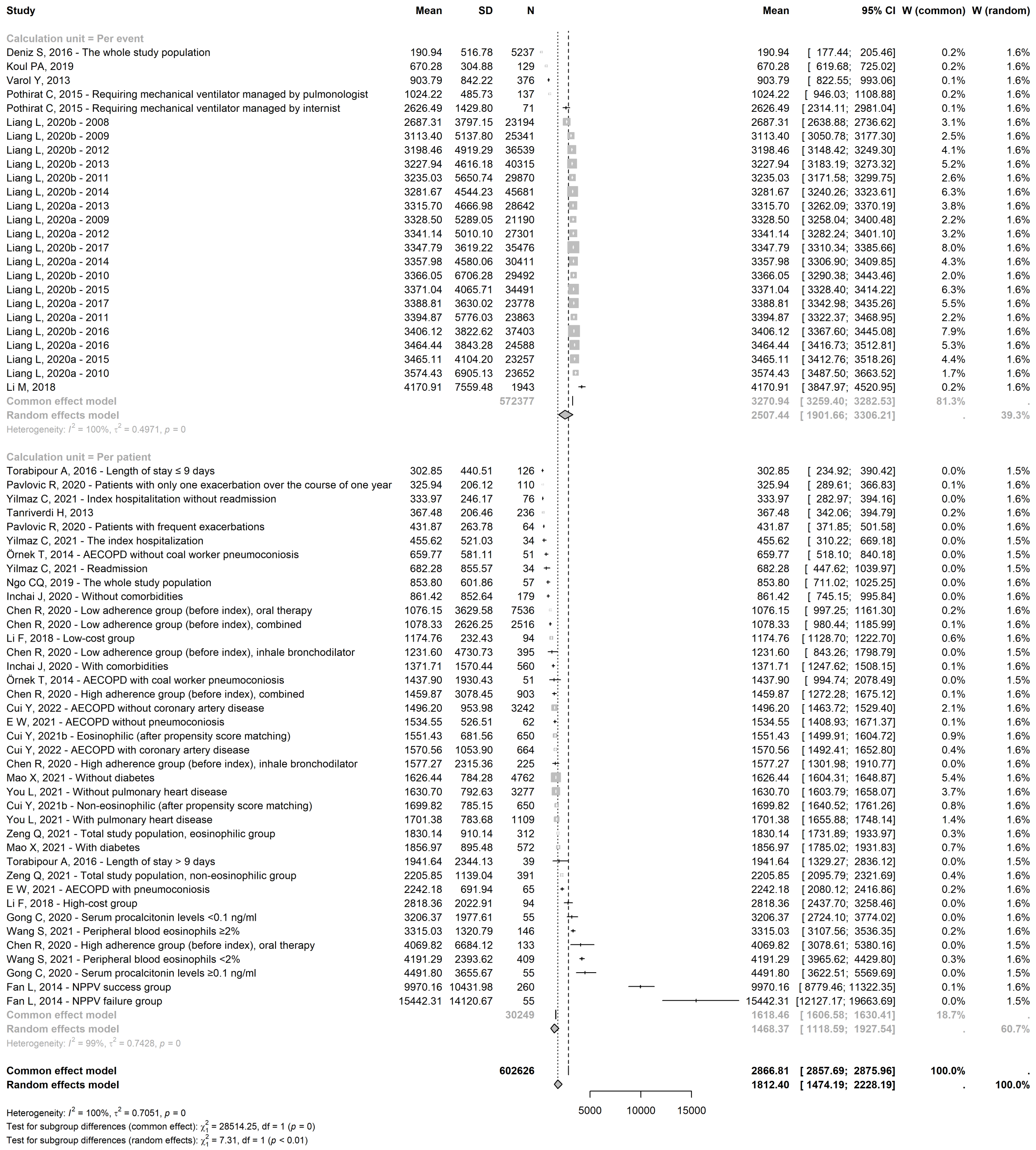

Figure S4.6 Meta-analysis of hospitalization costs in 2021 USD of AECOPD by calculation unit

4-II Medication costs

4.7 COPD by country income group

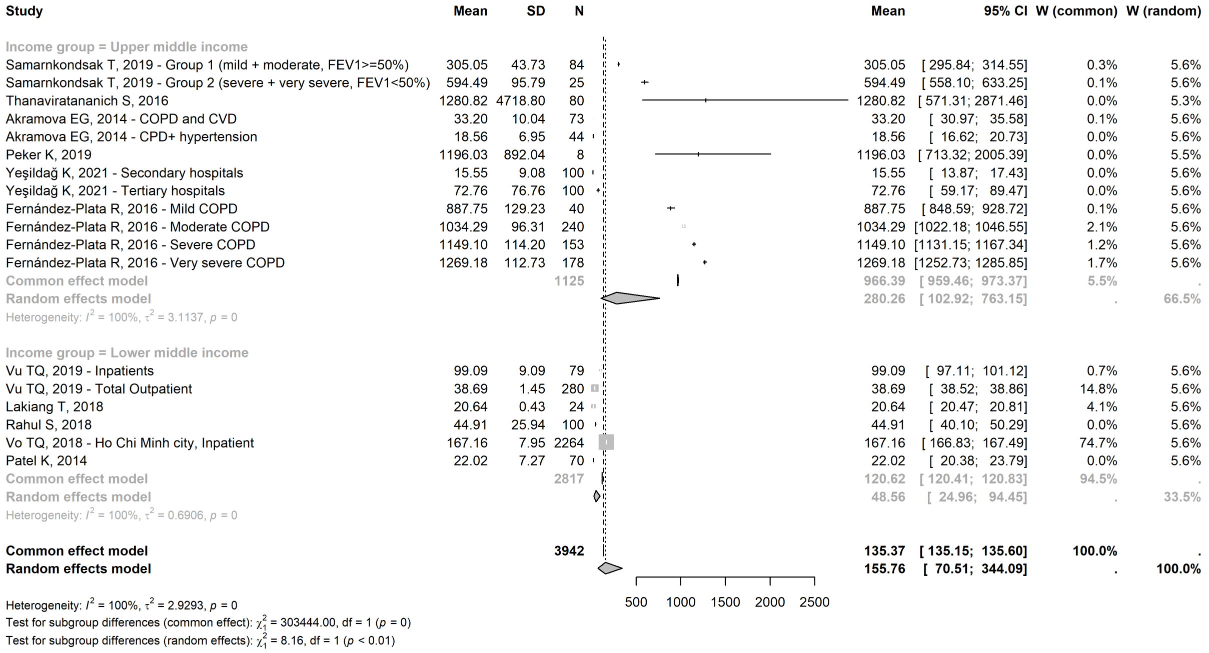

Figure S4.7 Meta-analysis of medication costs in 2021 USD of COPD by income group

4.8 COPD by country

Study	Mean	SD	N	Mean	95% CI	W (common)	W (random)
Country = Thailand							
Samarnkondsak T, 2019 - Group 1 (mild + moderate, FEV1>=50%)	305.05	43.73	84	305.05	[295.84; 314.55]	0.3%	5.6%
Samarnkondsak T, 2019 - Group 2 (severe + very severe, FEV1<50%)	594.49	95.79	25	594.49	[558.10; 633.25]	0.1%	5.6%
Thanaviratananich S, 2016	1280.82	4718.80	80	1280.82	[571.31; 2871.46]	0.0%	5.3%
Common effect model			189	346.96	[337.53; 356.66]	0.4%	.
Random effects model				567.13	[266.29; 1207.82]	.	16.4%
Heterogeneity: I^2 = 99%, τ^2 = 0.4017, p < 0.01							
Country = Vietnam							
Vu TQ, 2019 - Inpatients	99.09	9.09	79	99.09	[97.11; 101.12]	0.7%	5.6%
Vu TQ, 2019 - Total Outpatient	38.69	1.45	280	38.69	[38.52; 38.86]	14.8%	5.6%
Vo TQ, 2018 - Ho Chi Minh city, Inpatient	167.16	7.95	2264	167.16	[166.83; 167.49]	74.7%	5.6%
Common effect model			2623	130.93	[130.70; 131.16]	90.3%	.
Random effects model				86.22	[37.25; 199.54]	.	16.7%
Heterogeneity: I^2 = 100%, τ^2 = 0.5499, p = 0							
Country = Russia							
Akramova EG, 2014 - COPD and CVD	33.20	10.04	73	33.20	[30.97; 35.58]	0.1%	5.6%
Akramova EG, 2014 - CPD+ hypertension	18.56	6.95	44	18.56	[16.62; 20.73]	0.0%	5.6%
Common effect model			117	28.18	[26.57; 29.88]	0.1%	.
Random effects model				24.86	[14.06; 43.96]	.	11.2%
Heterogeneity: I^2 = 99%, τ^2 = 0.1669, p < 0.01							
Country = India							
Lakiang T, 2018	20.64	0.43	24	20.64	[20.47; 20.81]	4.1%	5.6%
Rahul S, 2018	44.91	25.94	100	44.91	[40.10; 50.29]	0.0%	5.6%
Patel K, 2014	22.02	7.27	70	22.02	[20.38; 23.79]	0.0%	5.6%
Common effect model			194	20.74	[20.57; 20.91]	4.2%	.
Random effects model				27.26	[16.77; 44.33]	.	16.7%
Heterogeneity: I^2 = 99%, τ^2 = 0.1829, p < 0.01							
Country = Turkey							
Peker K, 2019	1196.03	892.04	8	1196.03	[713.32; 2005.39]	0.0%	5.5%
Yeşildağ K, 2021 - Secondary hospitals	15.55	9.08	100	15.55	[13.87; 17.43]	0.0%	5.6%
Yeşildağ K, 2021 - Tertiary hospitals	72.76	76.76	100	72.76	[59.17; 89.47]	0.0%	5.6%
Common effect model			208	25.78	[23.37; 28.44]	0.0%	.
Random effects model				109.43	[9.13; 1311.82]	.	16.6%
Heterogeneity: I^2 = 99%, τ^2 = 4.7903, p < 0.01							
Country = Mexico							
Fernández-Plata R, 2016 - Mild COPD	887.75	129.23	40	887.75	[848.59; 928.72]	0.1%	5.6%
Fernández-Plata R, 2016 - Moderate COPD	1034.29	96.31	240	1034.29	[1022.18; 1046.55]	2.1%	5.6%
Fernández-Plata R, 2016 - Severe COPD	1149.10	114.20	153	1149.10	[1131.15; 1167.34]	1.2%	5.6%
Fernández-Plata R, 2016 - Very severe COPD	1269.18	112.73	178	1269.18	[1252.73; 1285.85]	1.7%	5.6%
Common effect model			611	1129.59	[1121.11; 1138.13]	5.1%	.
Random effects model				1076.79	[927.74; 1249.78]	.	22.3%
Heterogeneity: I^2 = 100%, τ^2 = 0.0229, p < 0.01							
**Common effect model**			**3942**	**135.37**	**[135.15; 135.60]**	**100.0%**	**.**
**Random effects model**				**155.76**	**[70.51; 344.09]**	**.**	**100.0%**

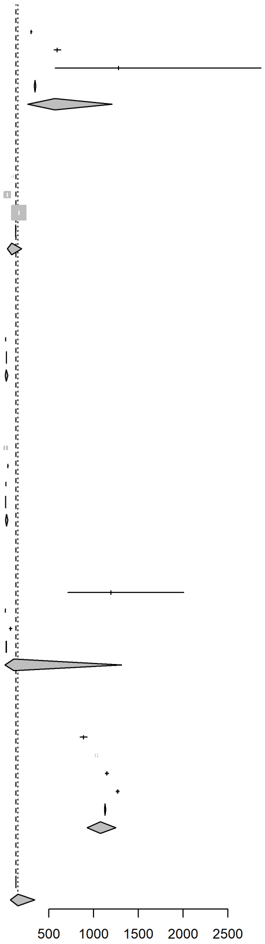

Heterogeneity: I^2 = 100%, τ^2 = 2.9293, p = 0
Test for subgroup differences (common effect): χ^2_5 = 511945.53, df = 5 (p = 0)
Test for subgroup differences (random effects): χ^2_5 = 356.77, df = 5 (p < 0.01)

Figure S4.8 Meta-analysis of medication costs in 2021 USD of COPD by country

4.9 AECOPD by country income group

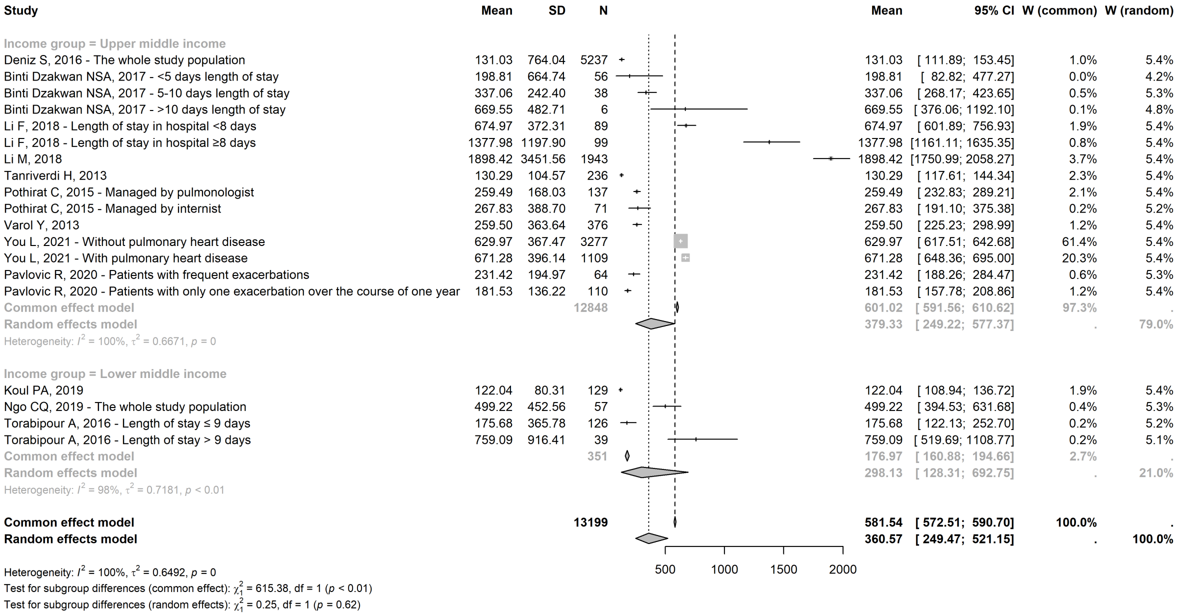

Figure S4.9 Meta-analysis of medication costs in 2021 USD of AECOPD by income group

4.10 AECOPD by country

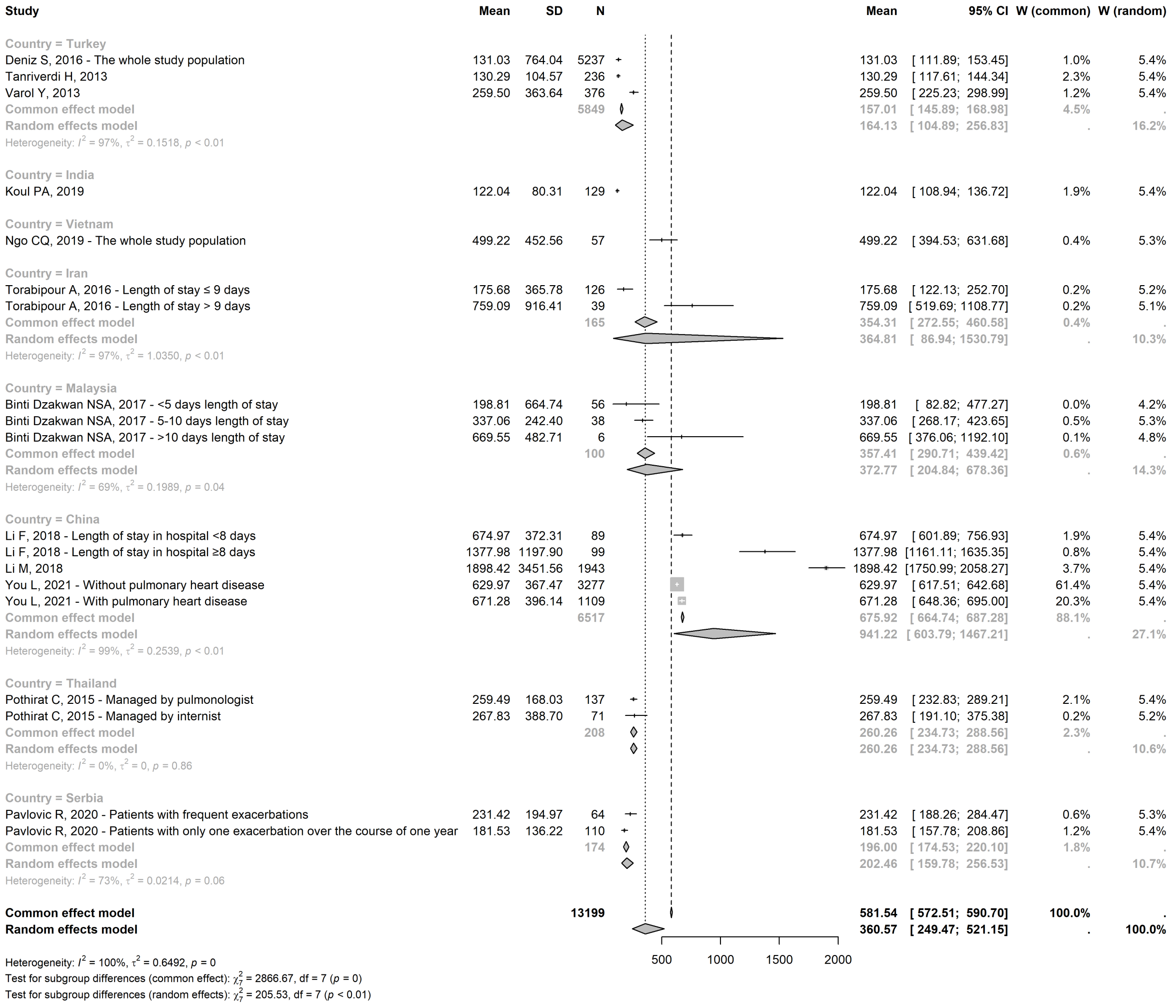

Figure S4.10 Meta-analysis of medication costs in 2021 USD of AECOPD by country

4-III Outpatient costs

4.11 COPD by country income group

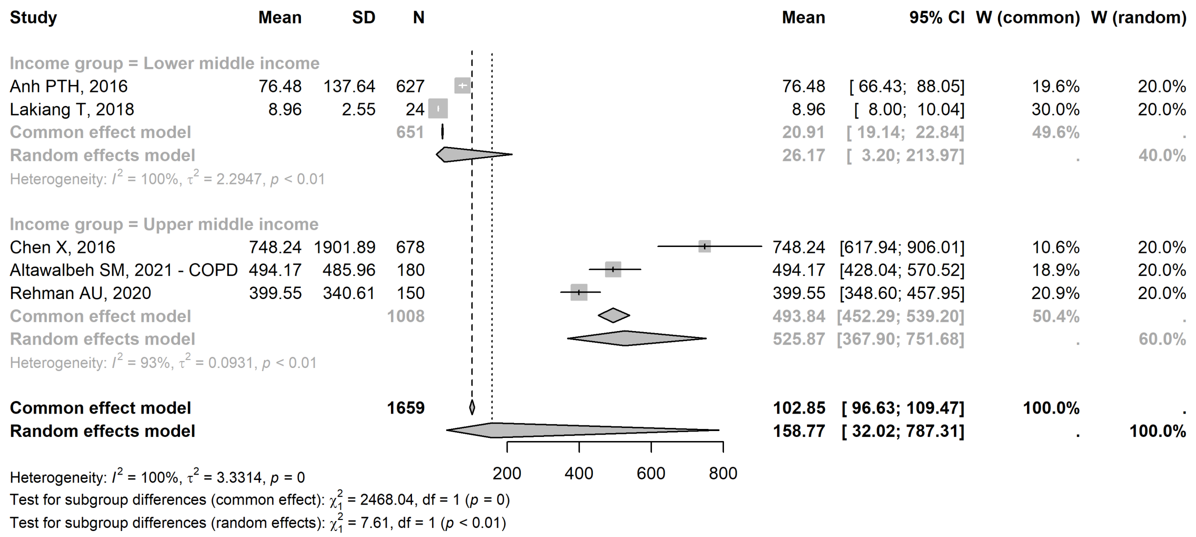

Figure S4.11 Meta-analysis of outpatient costs in 2021 USD of COPD by income group

4.12 COPD by country

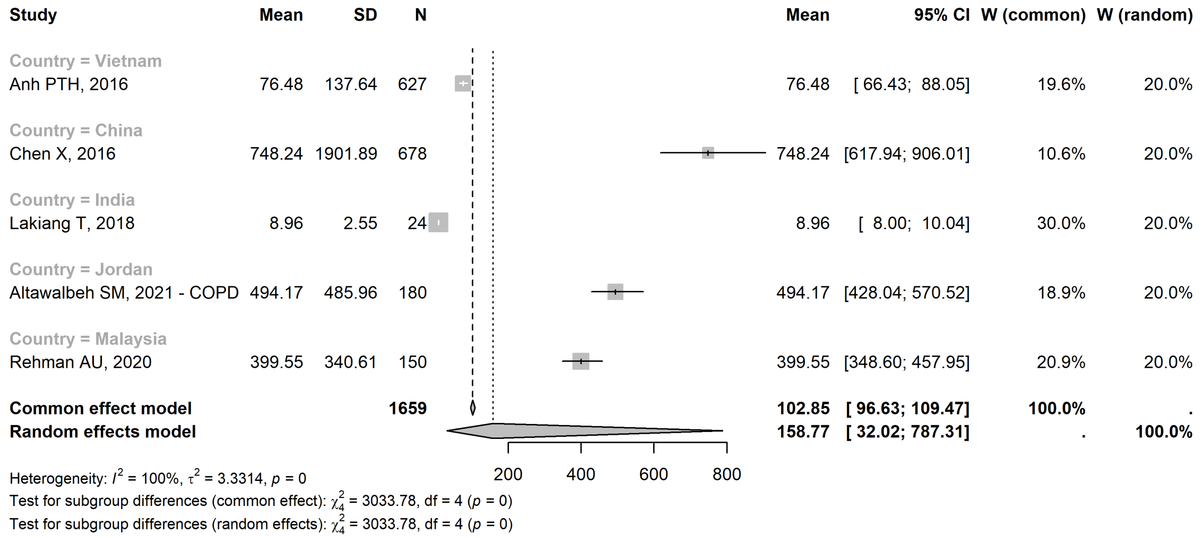

Figure S4.12 Meta-analysis of outpatient costs in 2021 USD of COPD by country
